# Supplementary figures and images for: gen3sis: A general engine for eco-evolutionary simulations of the processes that shape Earth’s biodiversity
Source: PLoS Biol. 2021 Jul 12;19(7):e3001340. doi: 10.1371/journal.pbio.3001340 (PMC8384074; doi:10.1371/journal.pbio.3001340)

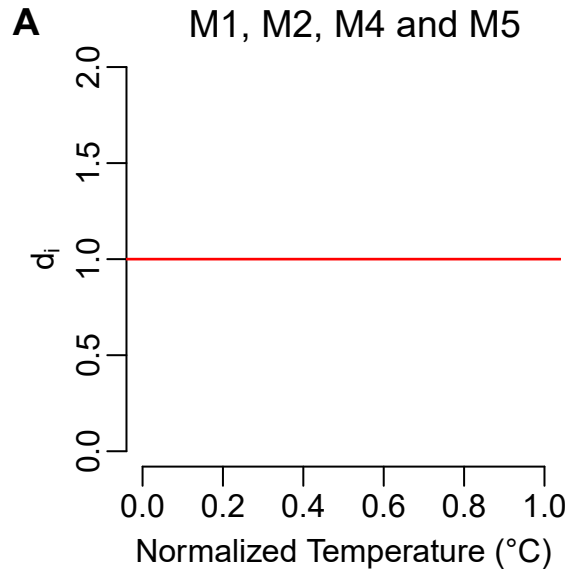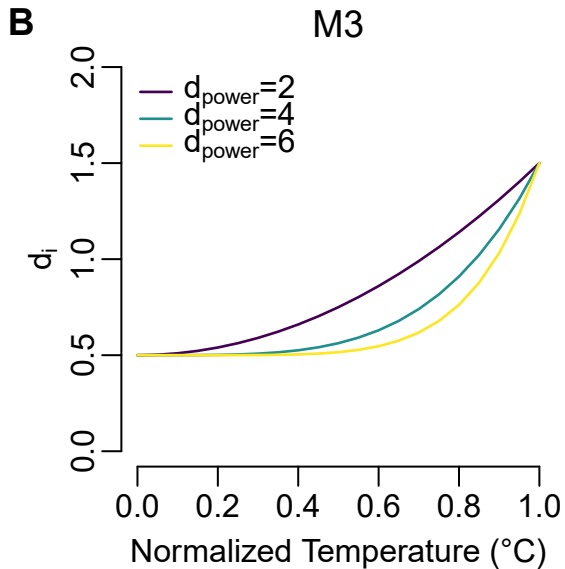

Supplement: S1 Fig — Divergence increase per time step di against the normalized occupied niche of isolated populations for models (A) M1, M2, M4, and M5, which assume temperature-independent divergence, and (B) M3, which assumes temperature-dependent divergence, where divergence relates to the mean of the realized temperature with 3 different dpower values. (PDF) [file pbio.3001340.s005.pdf]

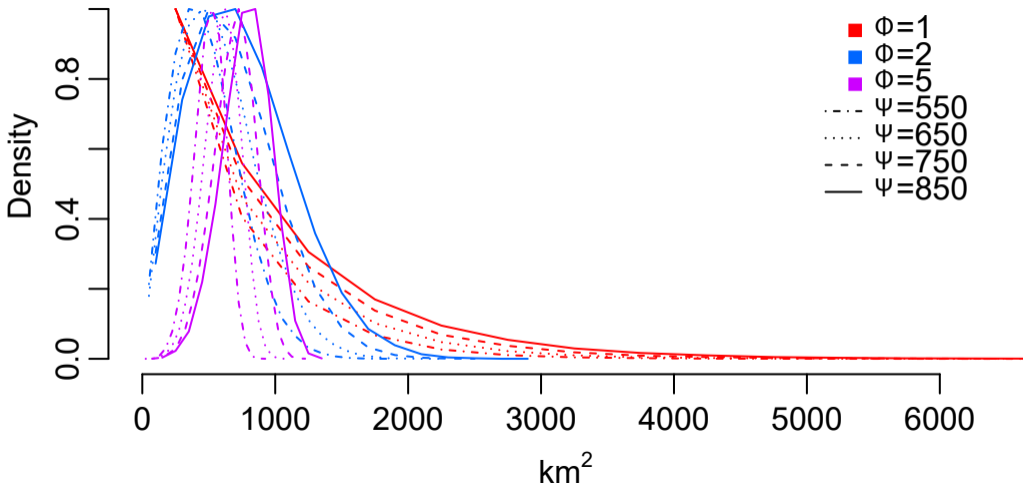

Supplement: S2 Fig — Data presented available in S2 Data at https://zenodo.org/record/5006413. (PDF) [file pbio.3001340.s006.pdf]

**A.1**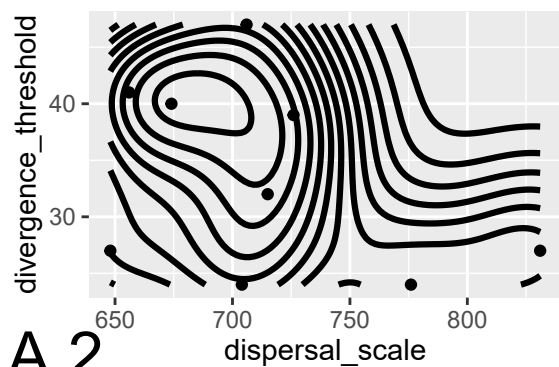**A.2**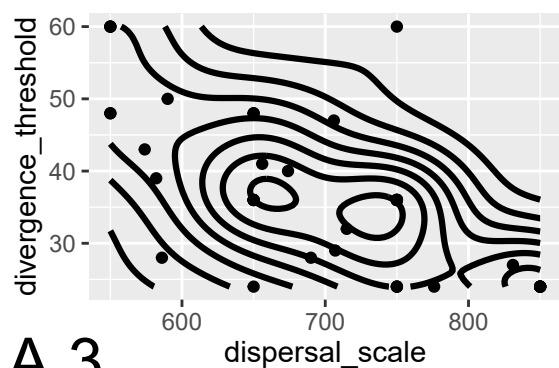**A.3**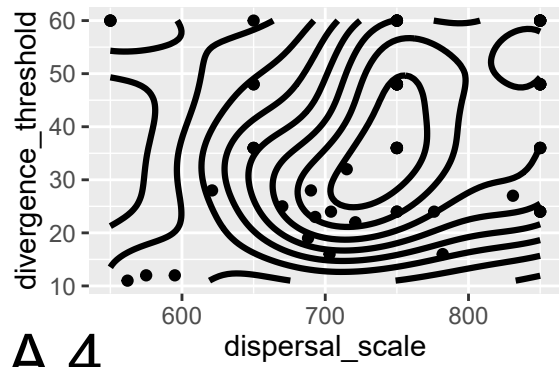**A.4**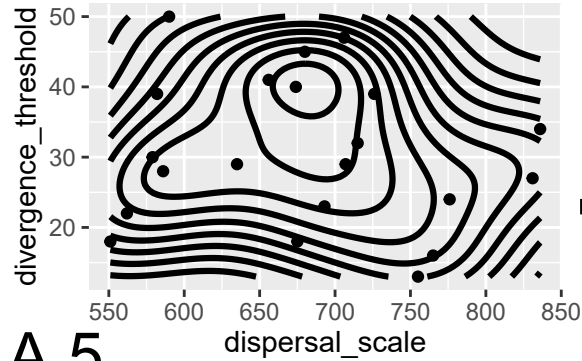**A.5**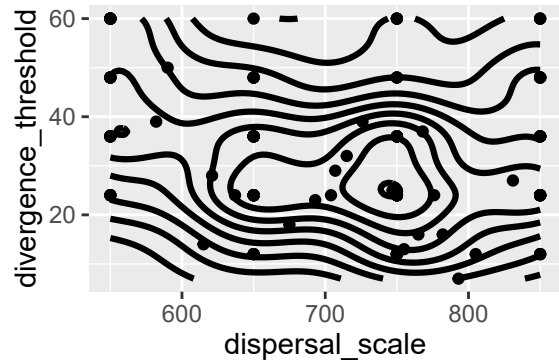**B.1**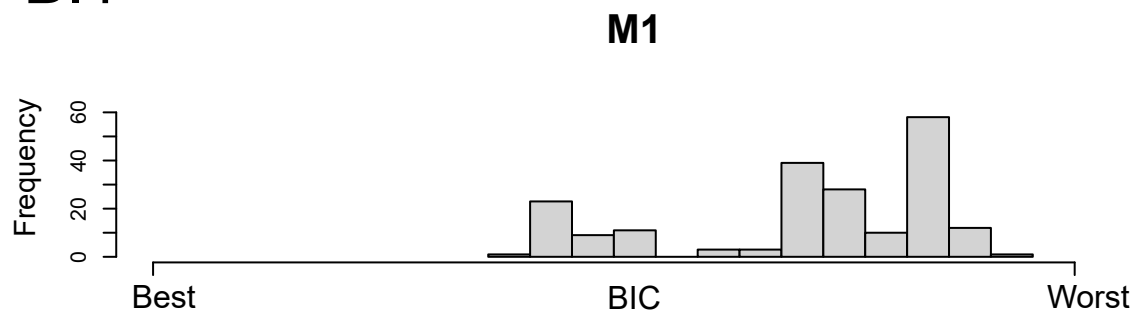**B.2**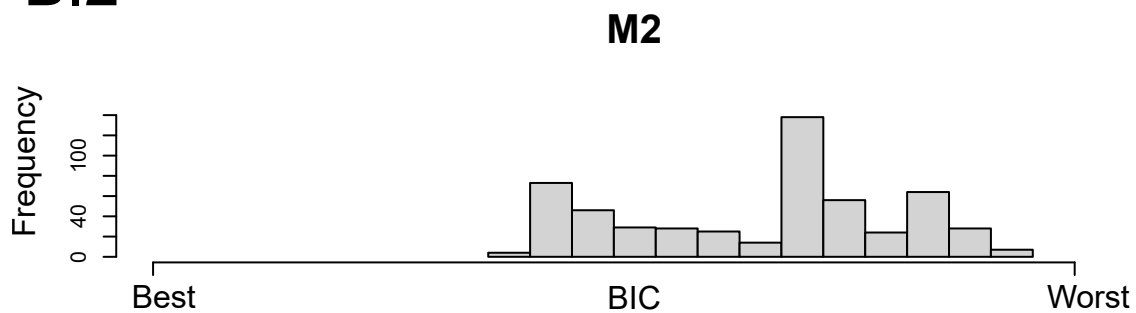**B.3**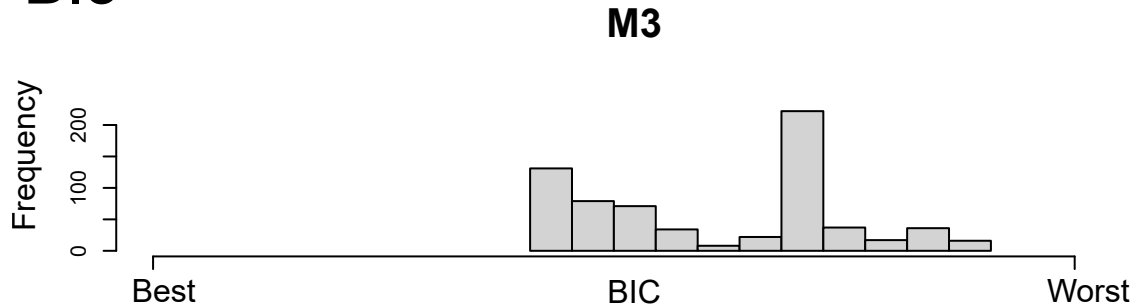**B.4**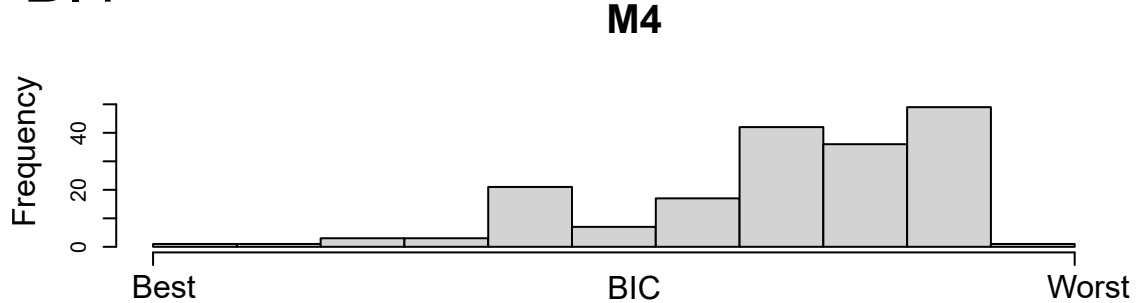**B.5**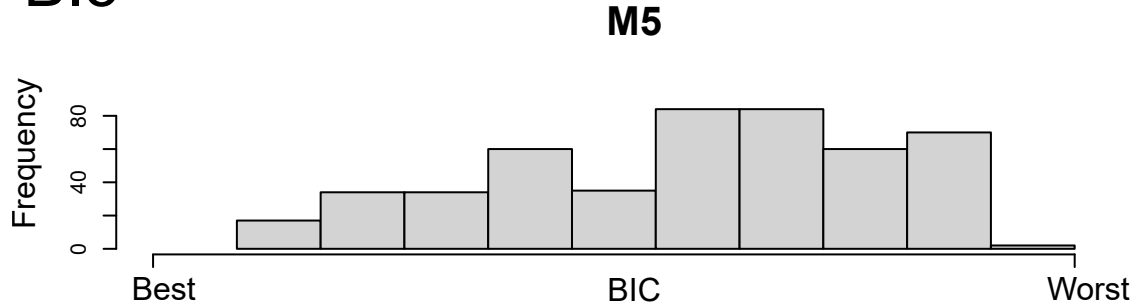

Supplement: S3 Fig — Models (i.e., M1, M2, M3, M4, and M5) (A) Kernel density estimate of the same explored parameters (i.e., divergence threshold and dispersal scale) for selected simulations based on a Pearson correlation of simulated versus best observed (i.e., cor > 0.4) and (B) performance quantified with the BIC. Omitted values from the parameter space were simulations generating an unacceptable best Pearson correlation to the empirical data (r ≤ 0.4), too many species (>35,000) or a weak richness gradient (<20 species between minimal and maximal α-richness). Data presented available in S3 Data at https://zenodo.org/record/5006413. BIC, Bayesian information criteria. (PDF) [file pbio.3001340.s007.pdf]

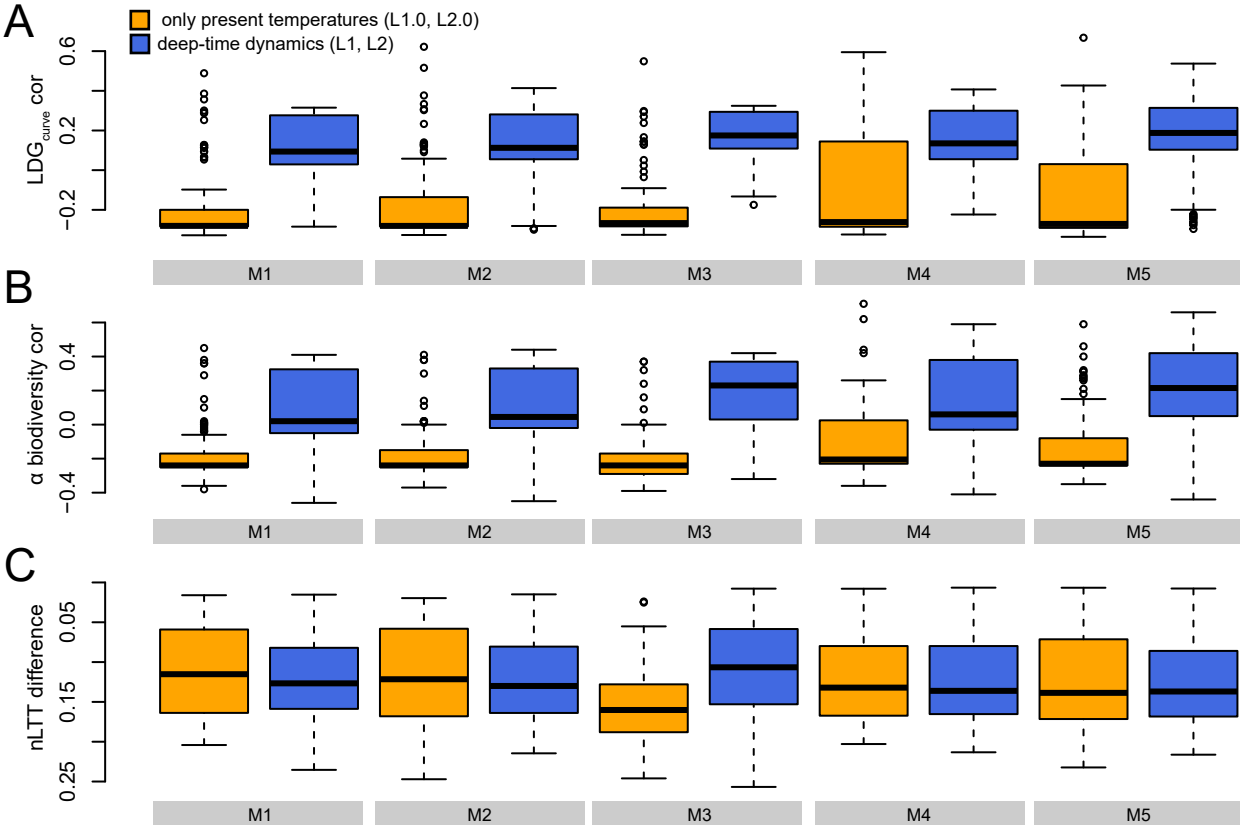

Supplement: S4 Fig — Summary statistics of the model fit to empirical data with and without environmental dynamics for (A) a Pearson correlation of standardized mean species number per latitude (LDGcurve), (B) a Pearson correlation of spatial α-diversity, and (C) the exact difference between lineage through time curves (nLTT). Data presented available in S2 Data at https://zenodo.org/record/5006413. nLTT, normalized lineage though time. (PDF) [file pbio.3001340.s008.pdf]

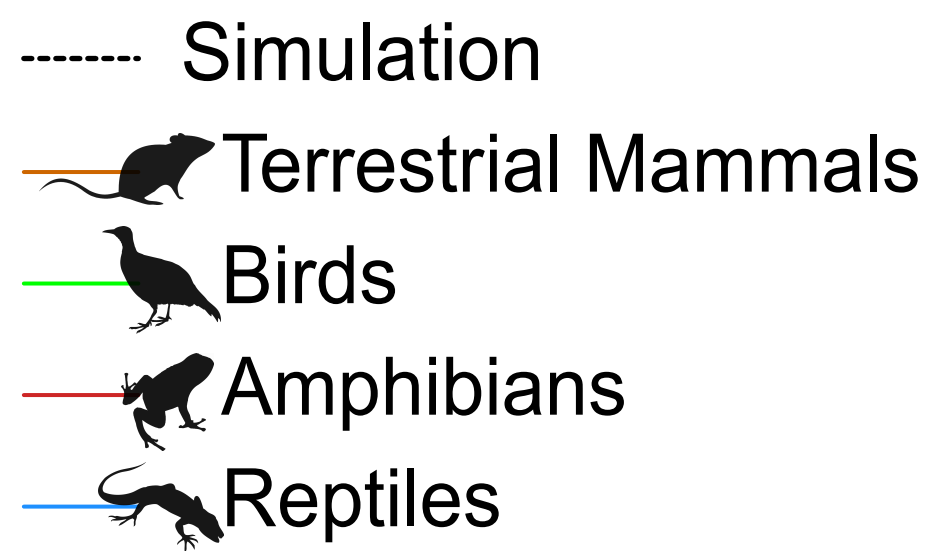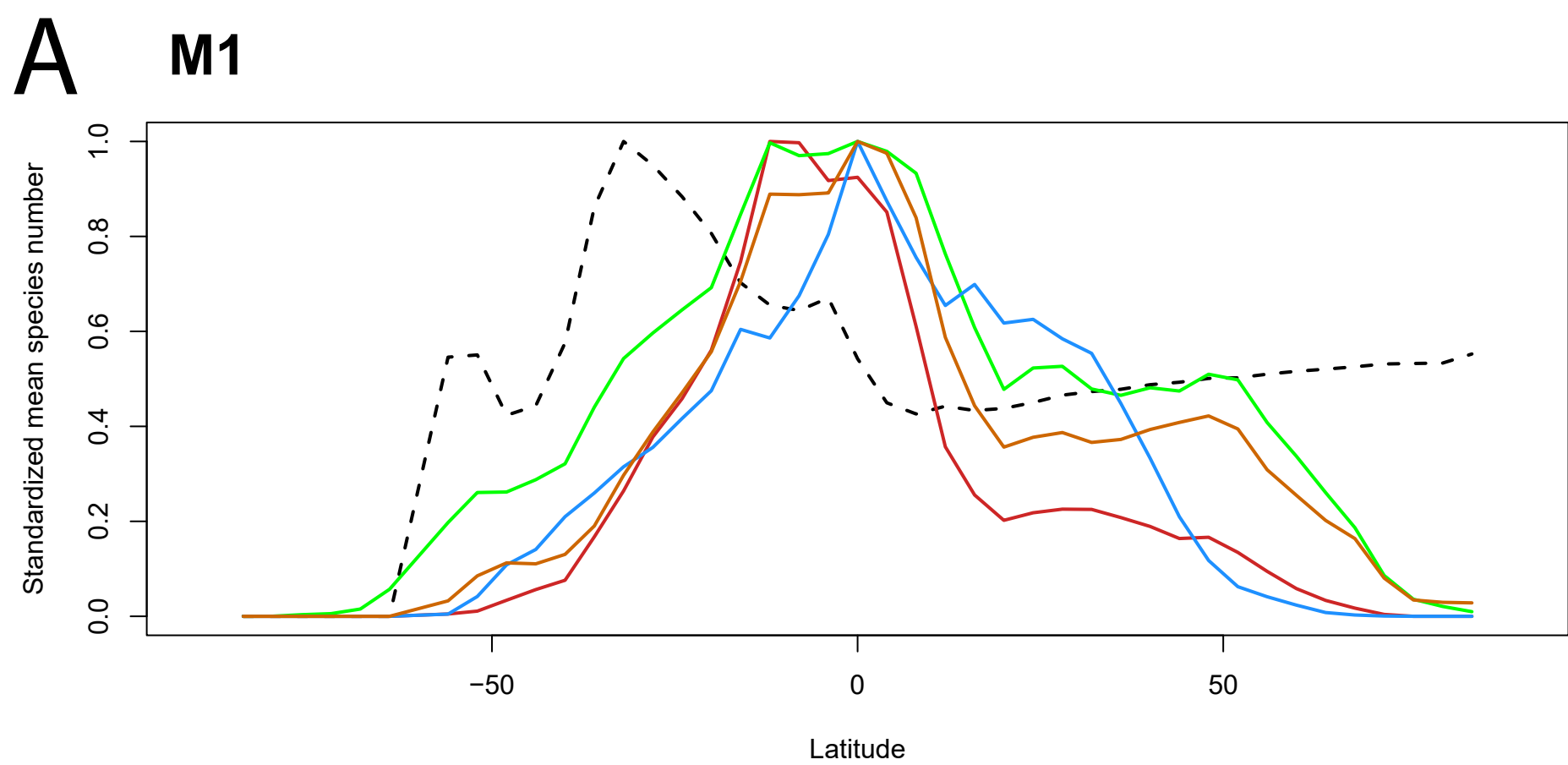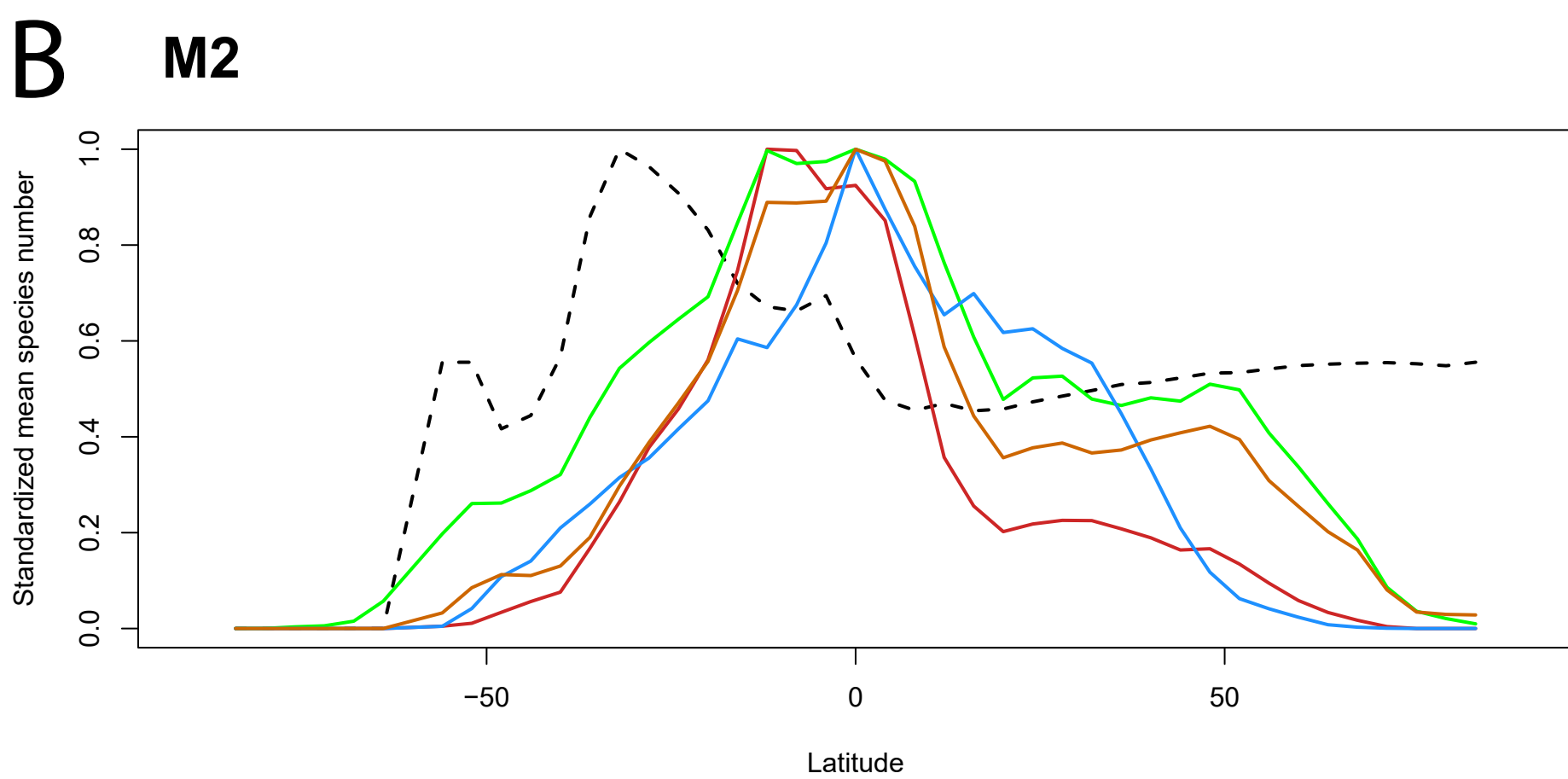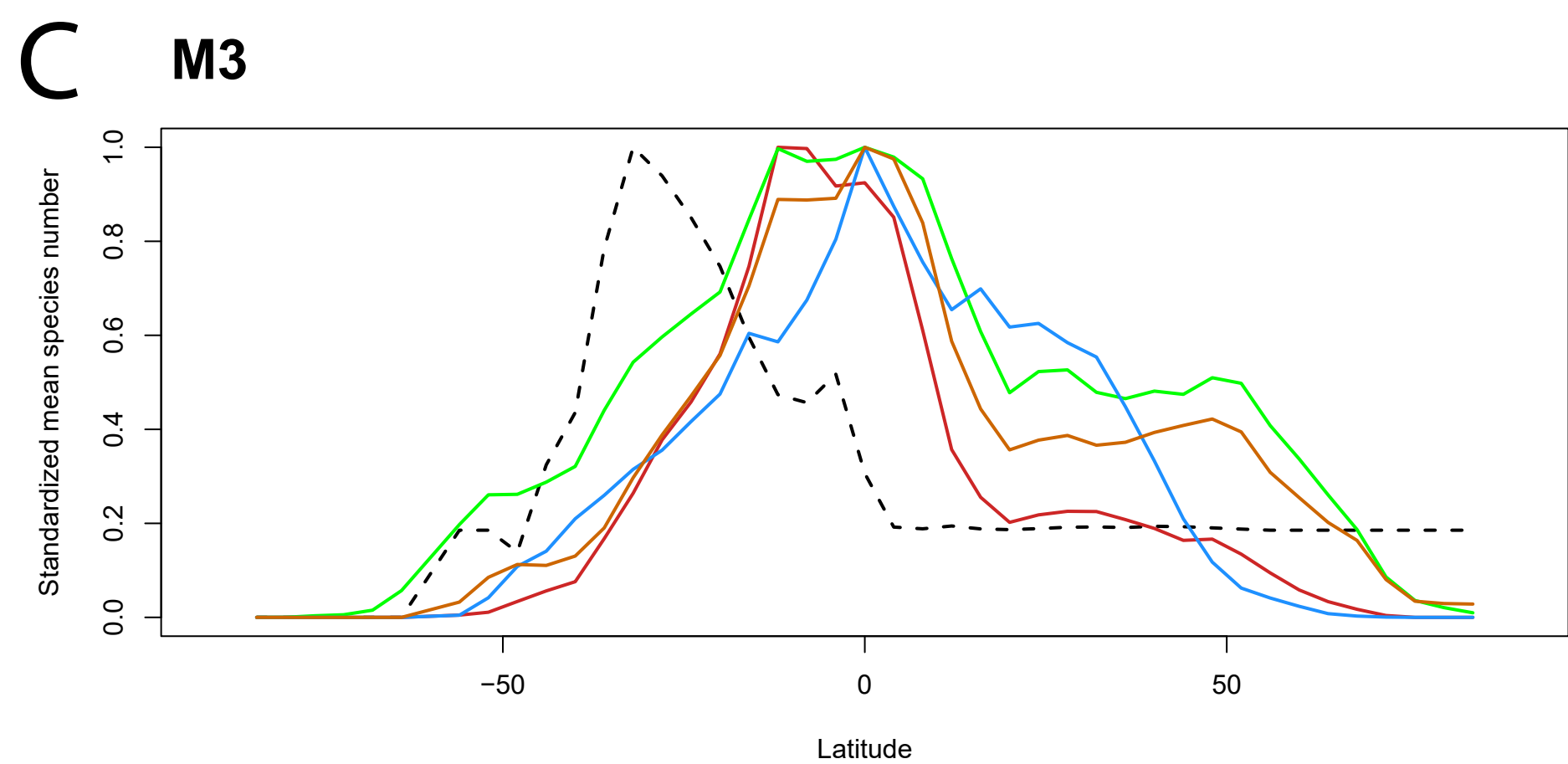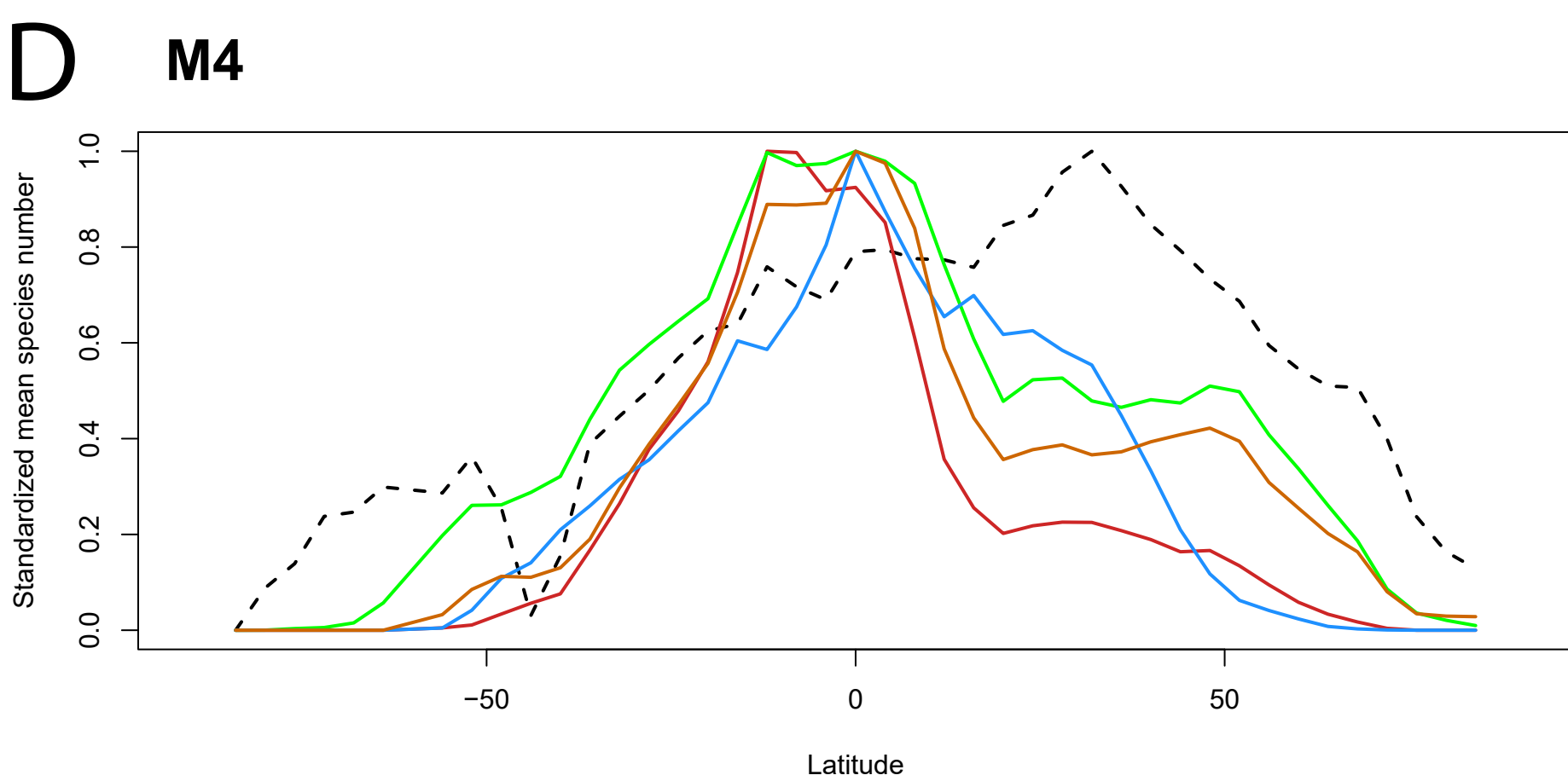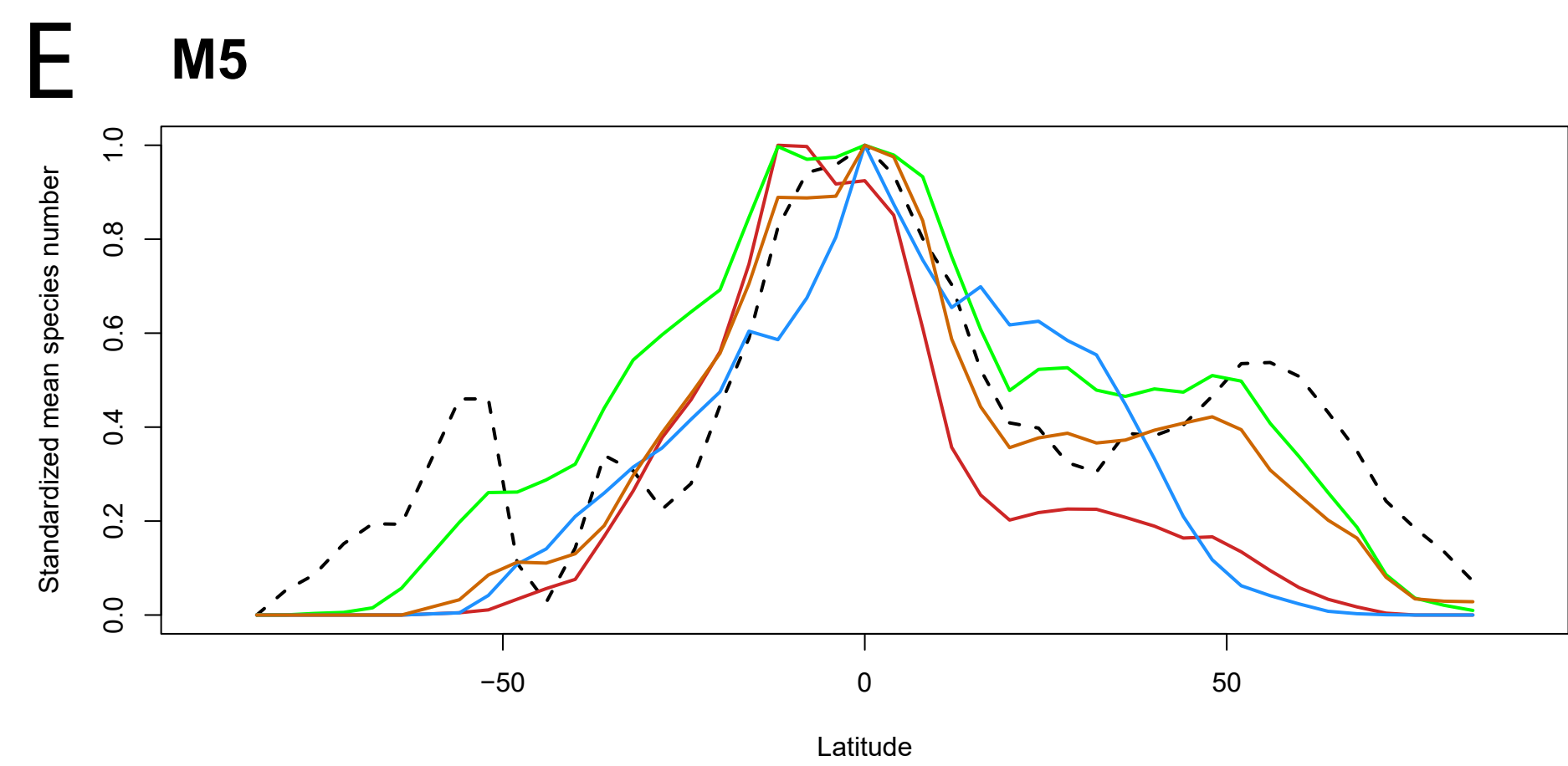

Supplement: S5 Fig — Standardized mean species number per latitude (LDGcurve) for empirical data (i.e., terrestrial mammals, birds, amphibians, and reptiles) and best matching simulation from models (A) M1, (B) M2, (C) M3, (D) M4, and (E) M5. Data presented available in S4 Data at https://zenodo.org/record/5006413. (PDF) [file pbio.3001340.s009.pdf]

A

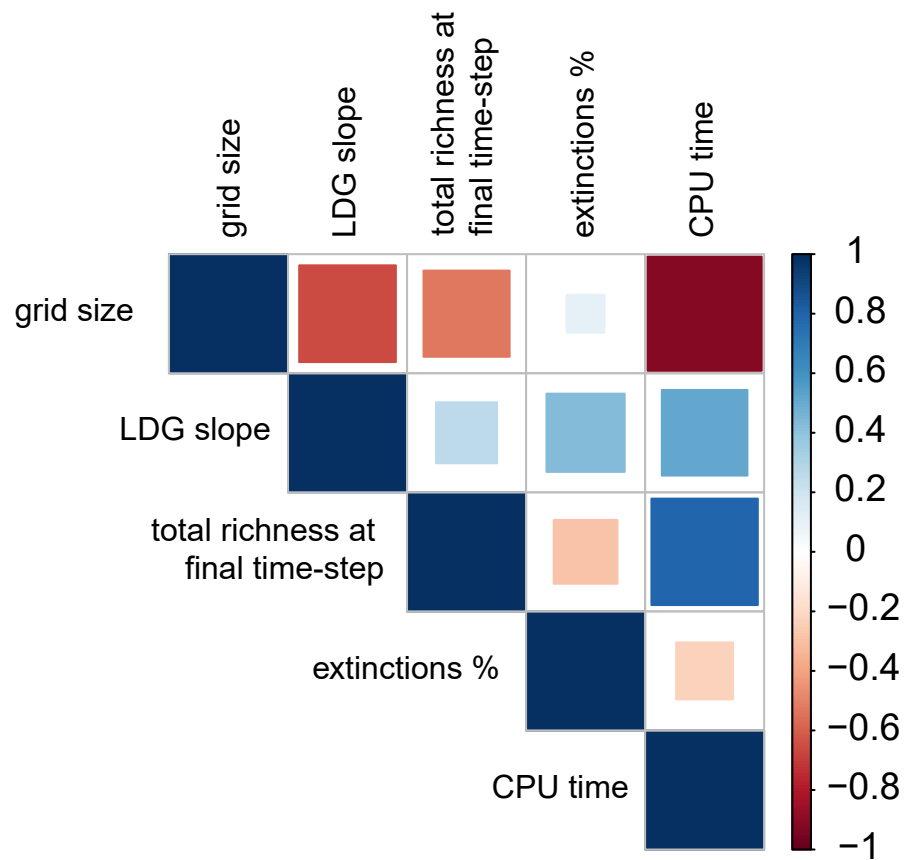

B

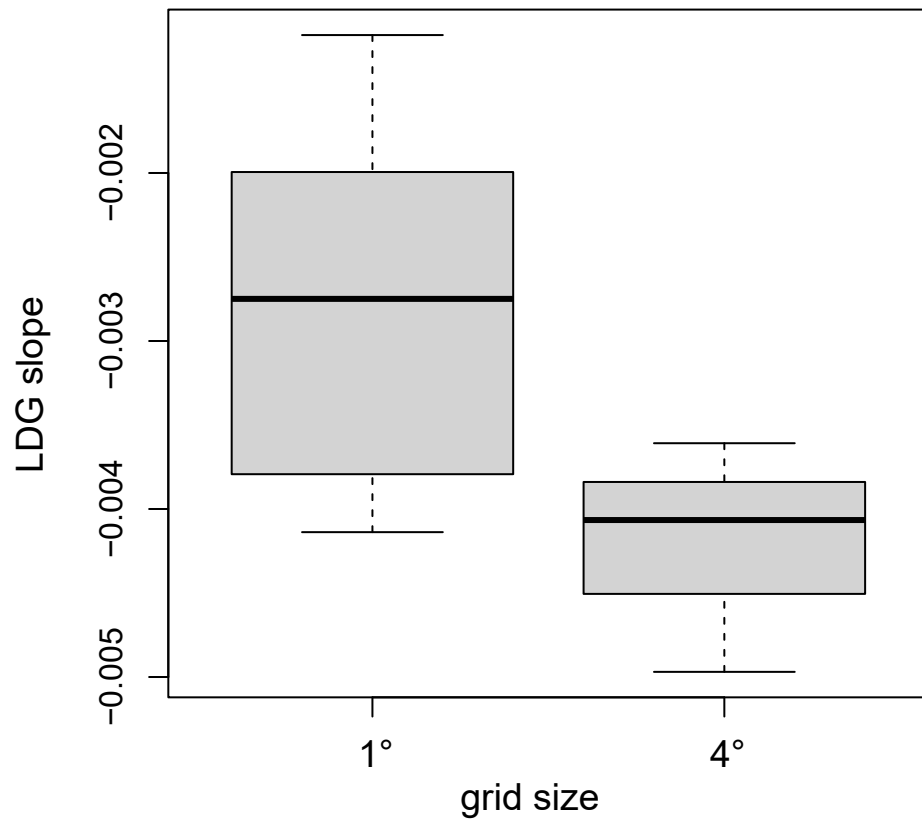

Supplement: S7 Fig — (A) Correlation of grid cell, LDG slope, and other summary statistics. (B) Simulated LDG slope and grid cell size, showing a significant effect of spatial resolution on LDG slope. Data presented available in S5 Data at https://zenodo.org/record/5006413. CPU, central processing unit; LDG, latitudinal diversity gradient. (PDF) [file pbio.3001340.s011.pdf]

# A Simulated

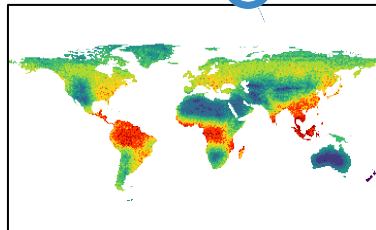

Normalized Richness

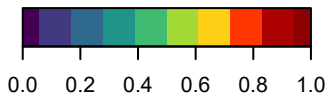

# B Terrestrial Mammals

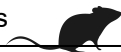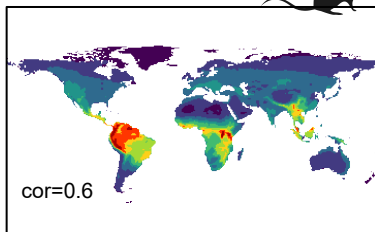

# C Birds

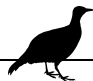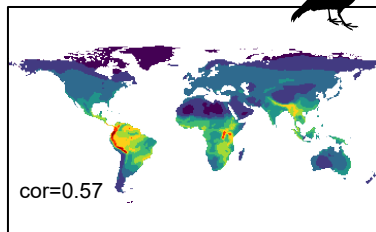

# D Amphibians

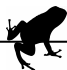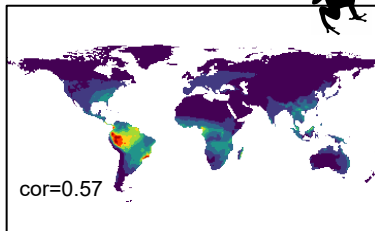

# E Reptiles

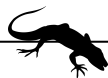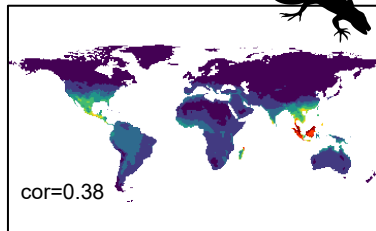

Supplement: S9 Fig — Normalized richness of (A) selected simulation, (B) terrestrial mammals, (C) birds, (D) amphibians, and (E) reptiles, with Pearson correlation values for comparisons between simulated and empirical data. (PDF) [file pbio.3001340.s013.pdf]

without environmental dynamics

with environmental dynamics

M1

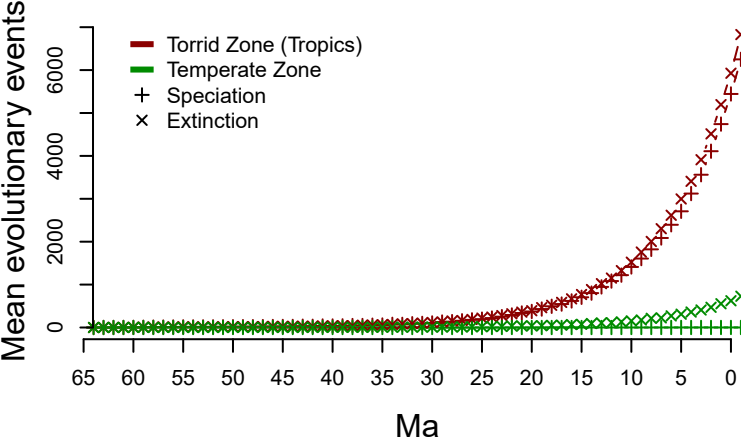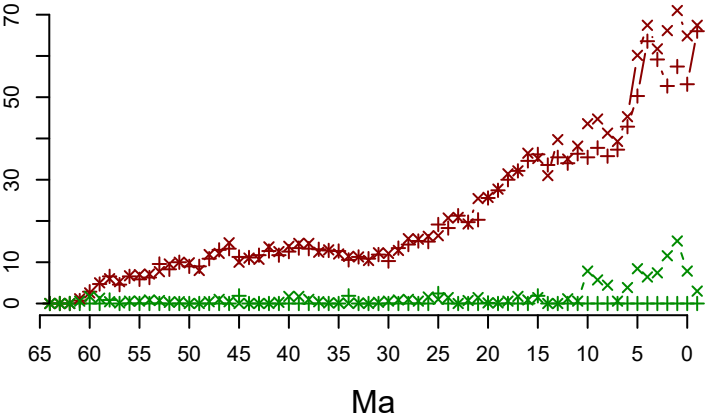

M2

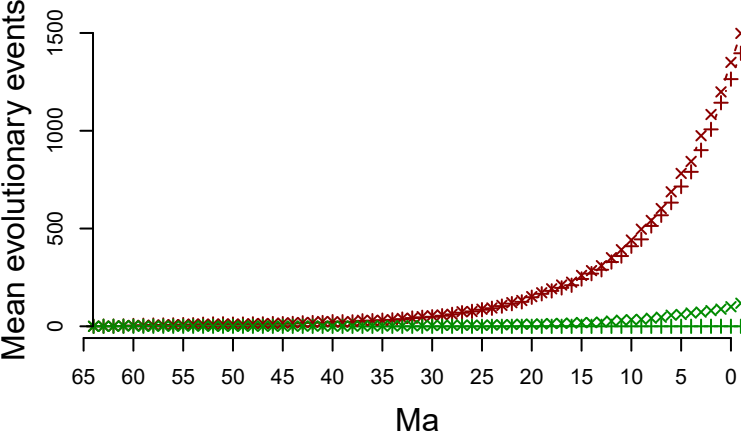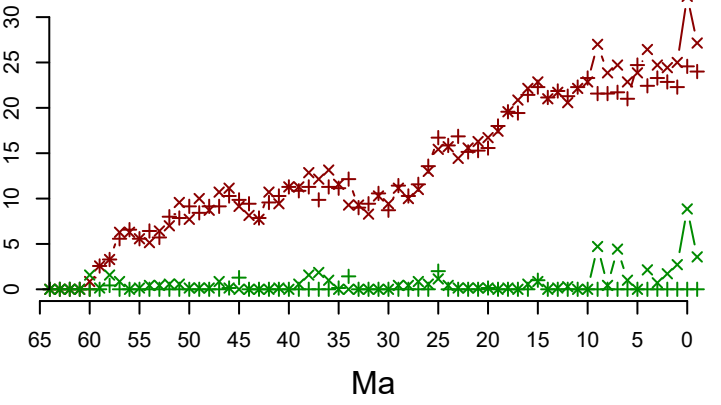

M3

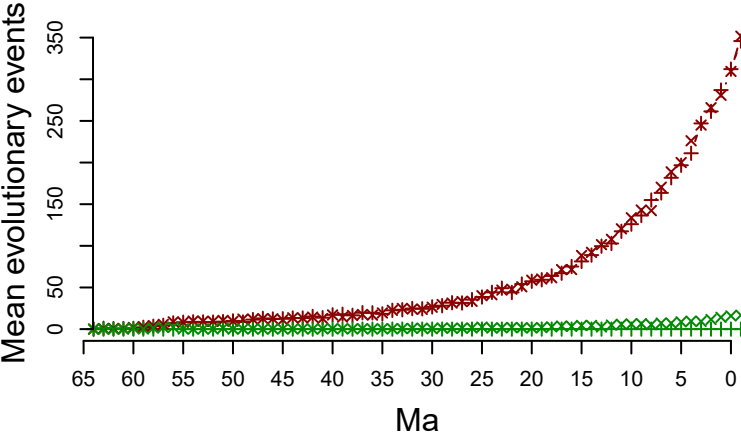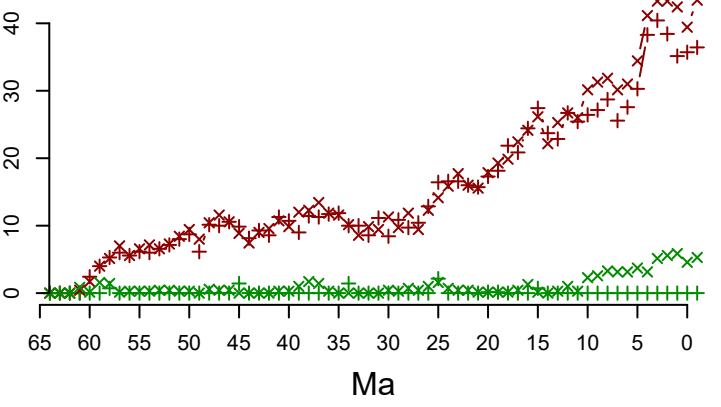

M4

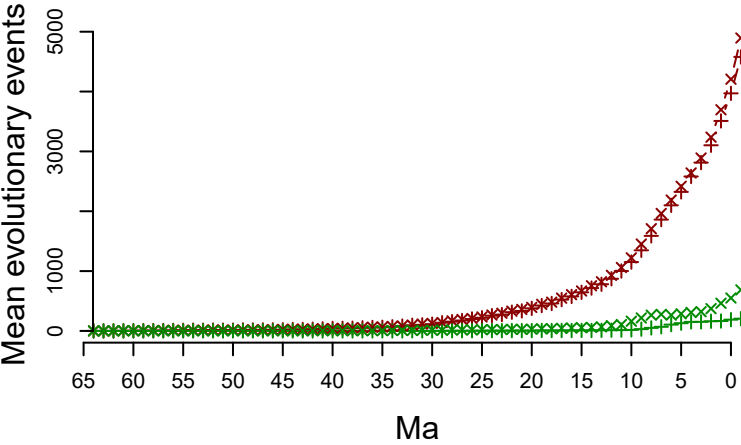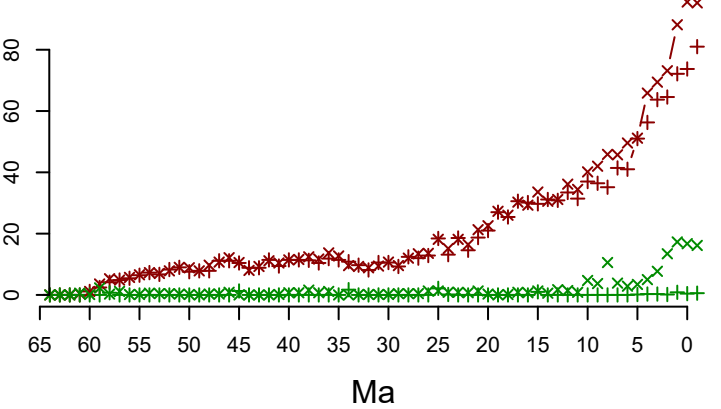

M5

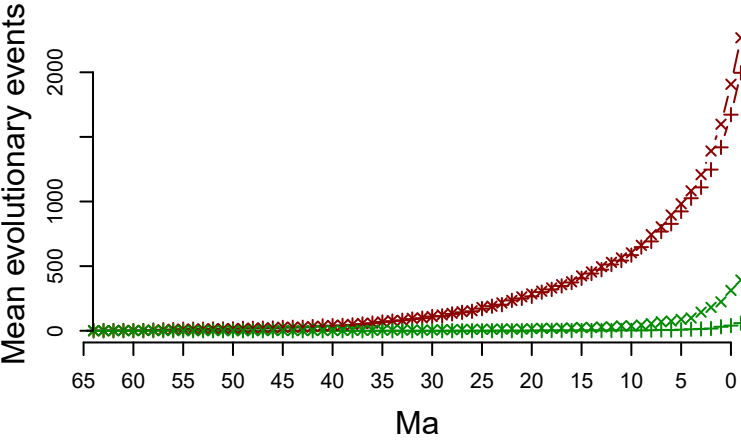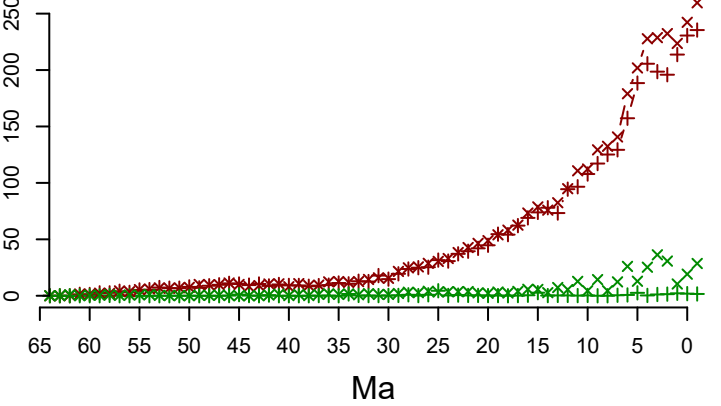

Supplement: S10 Fig — Data presented available in S6 Data at https://zenodo.org/record/5006413. (PDF) [file pbio.3001340.s014.pdf]

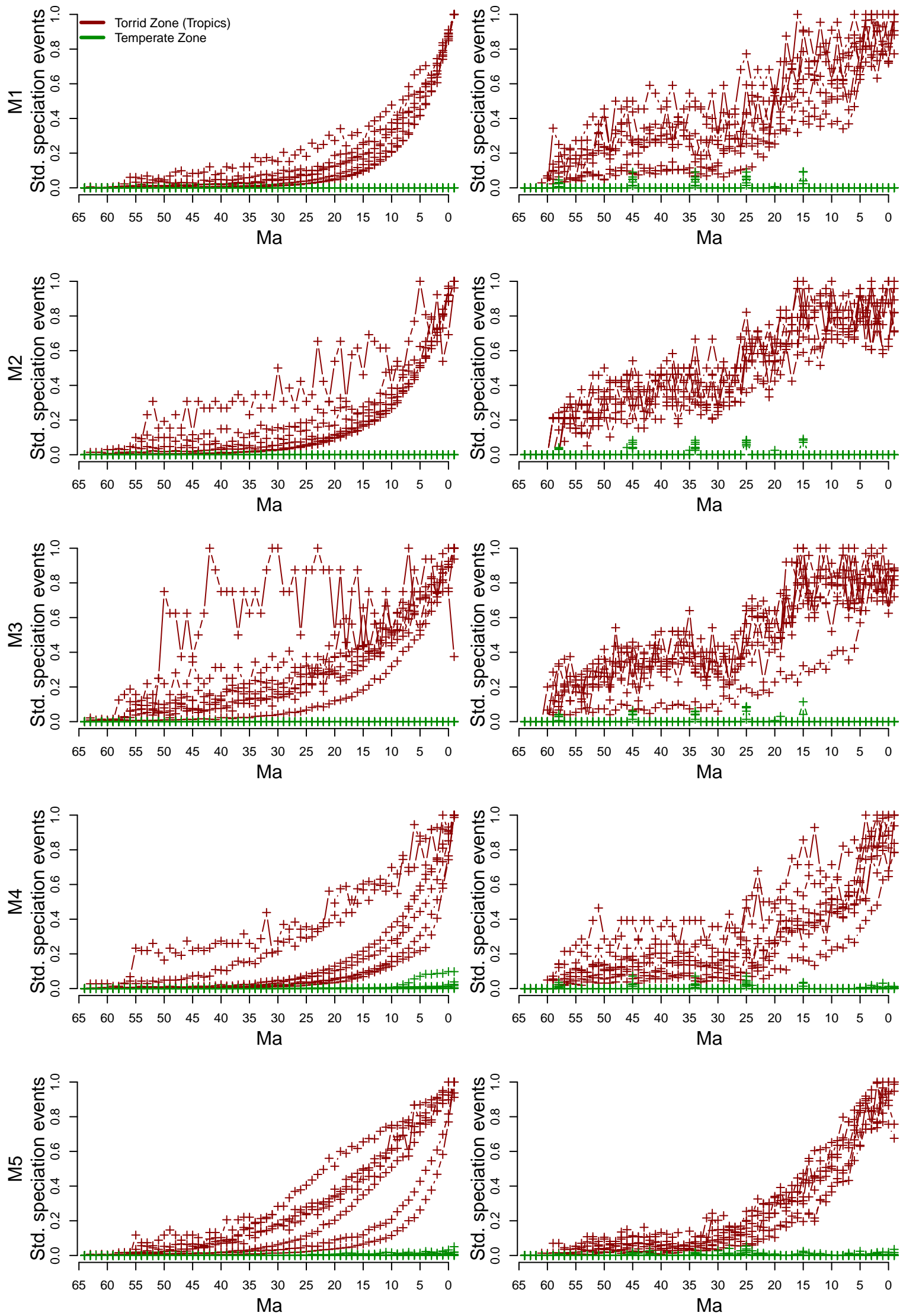

Supplement: S11 Fig — Data presented available in S6 Data at https://zenodo.org/record/5006413. (PDF) [file pbio.3001340.s015.pdf]

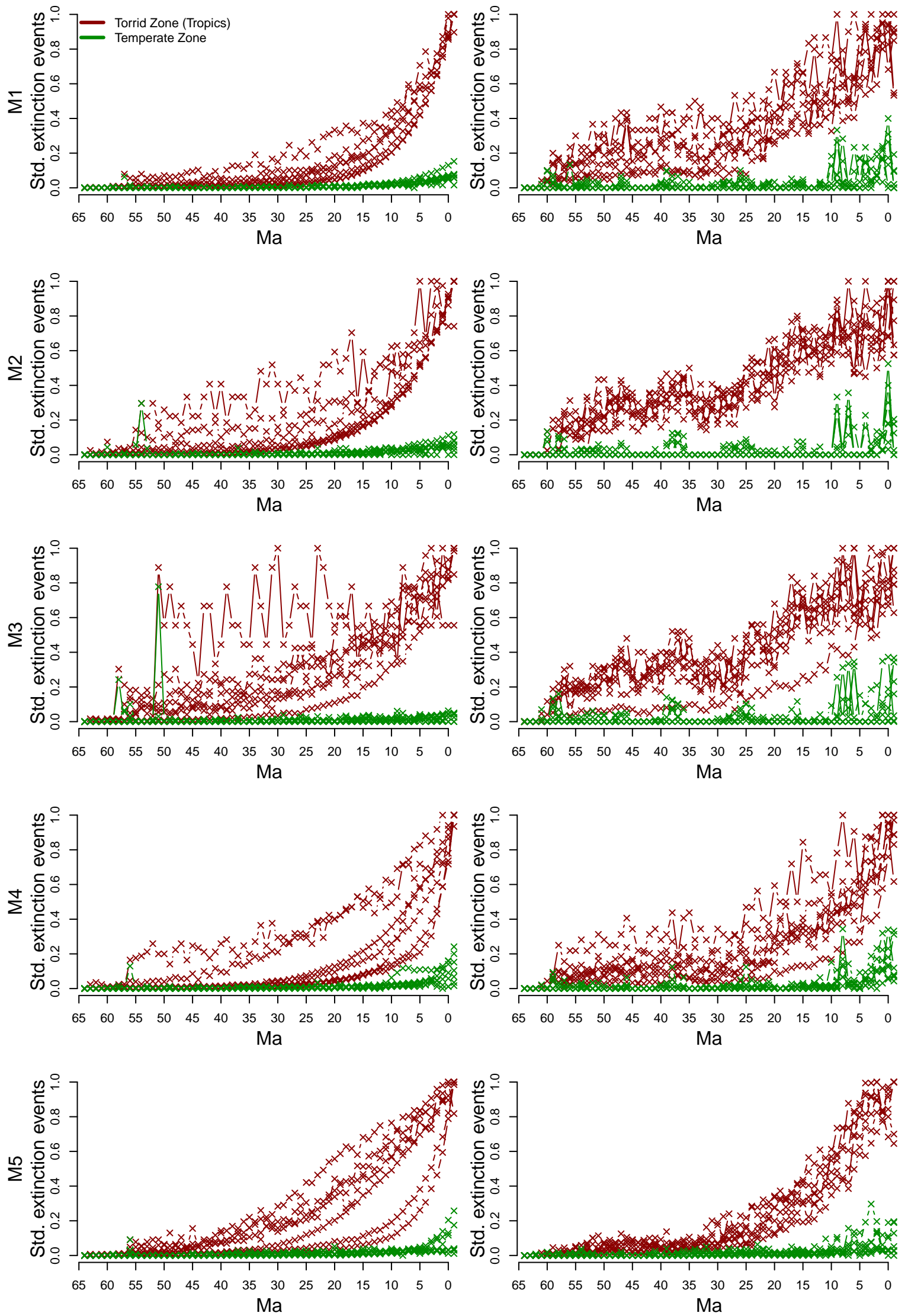

Supplement: S12 Fig — Data presented available in S6 Data at https://zenodo.org/record/5006413. (PDF) [file pbio.3001340.s016.pdf]

L1.0

L2.0

A M1 L1.0

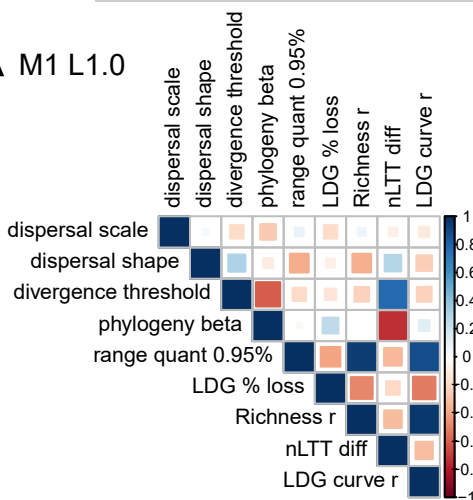

B M1 L2.0

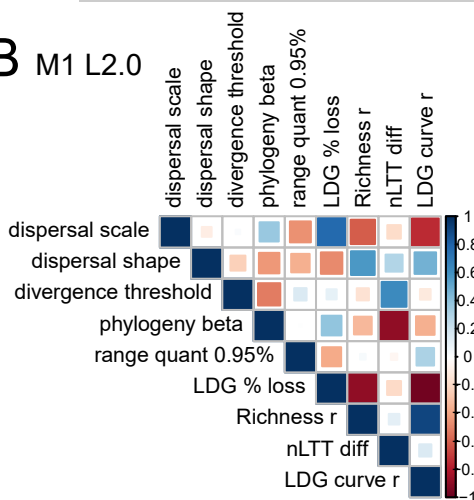

C M2 L1.0

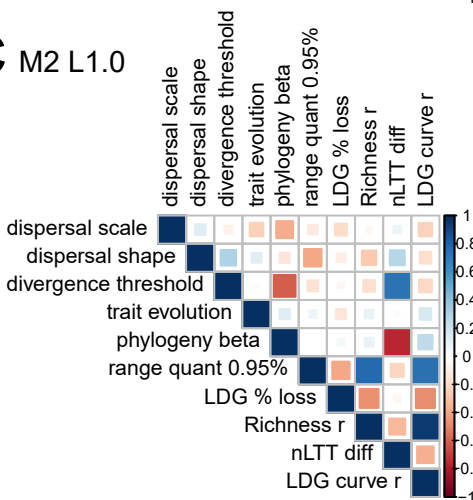

D M2 L2.0

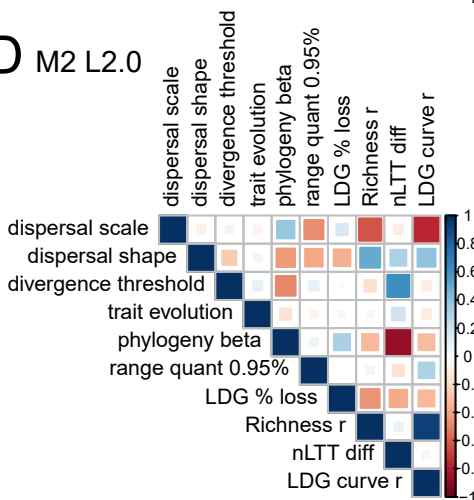

E M3 L1.0

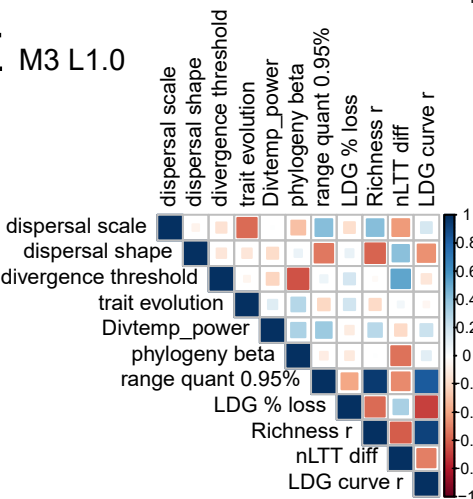

F M3 L2.0

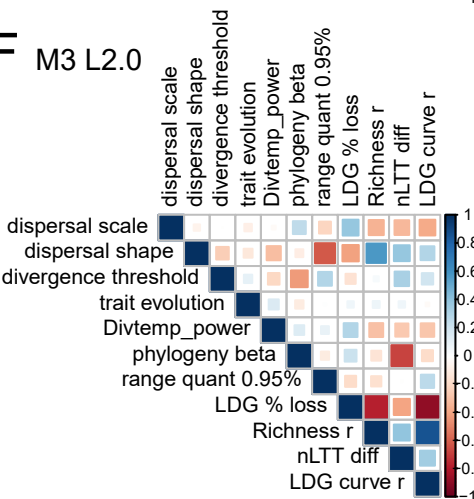

G M4 L1.0

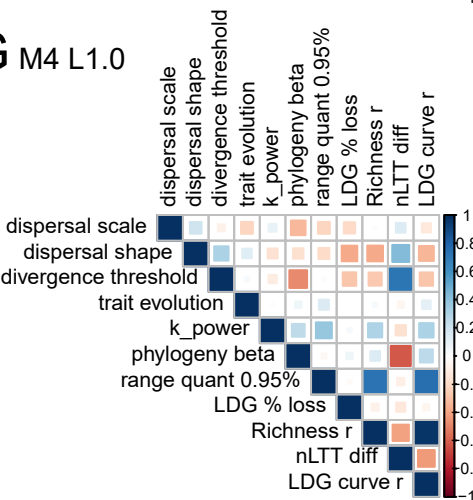

H M4 L2.0

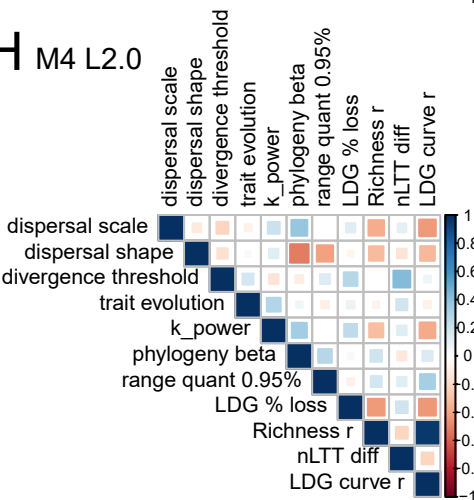

I M5 L1.0

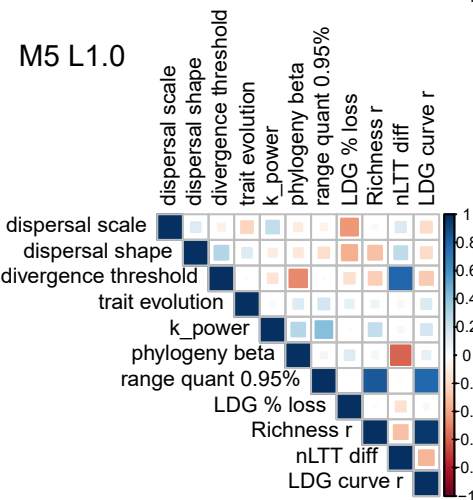

J M5 L2.0

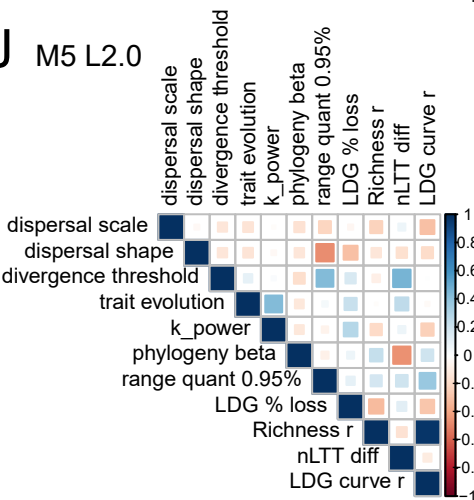

Supplement: S13 Fig — Correlation of model parameters and emerging patterns for all models and landscapes without deep-time environmental dynamics (A) M0 L1.0, (B) M0 L2.0, (C) M1 L1.0, (D) M1 L2.0, (E) M2 L1.0, and (F) M2 L2.0. Emerging patterns: (i) phylogeny beta is the phylogenetic tree imbalance statistic measured as the value that maximizes the likelihood in the β-splitting model; (ii) range quant 0.95% is the value of the 95% quantile of the species range area distribution; (iii) LDG % loss is the slope of the linear regression of species richness; (iv) richness r is the highest Pearson correlation between simulated and empirical α-diversity; (v) nLTT diff is the lowest difference between simulated and empirical nLTT curves; and (vi) LDG curve r is the highest Pearson correlation between simulated and empirical standardized mean species number per latitude. Data presented available in S3 Data at https://zenodo.org/record/5006413. LDG, latitudinal diversity gradient; nLTT, normalized lineage though time. (PDF) [file pbio.3001340.s017.pdf]

## A Landscape

Schematic  
Area Dynamics

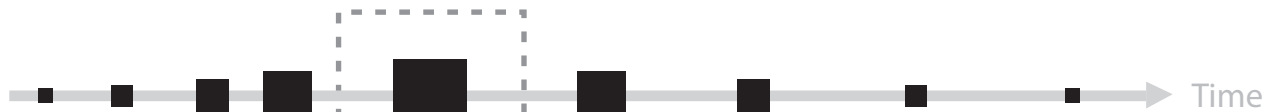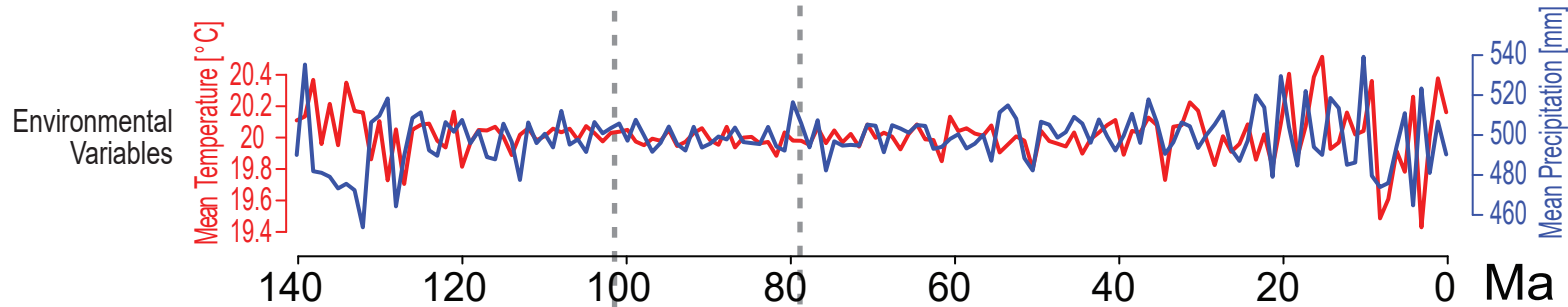

## B Emerging patterns

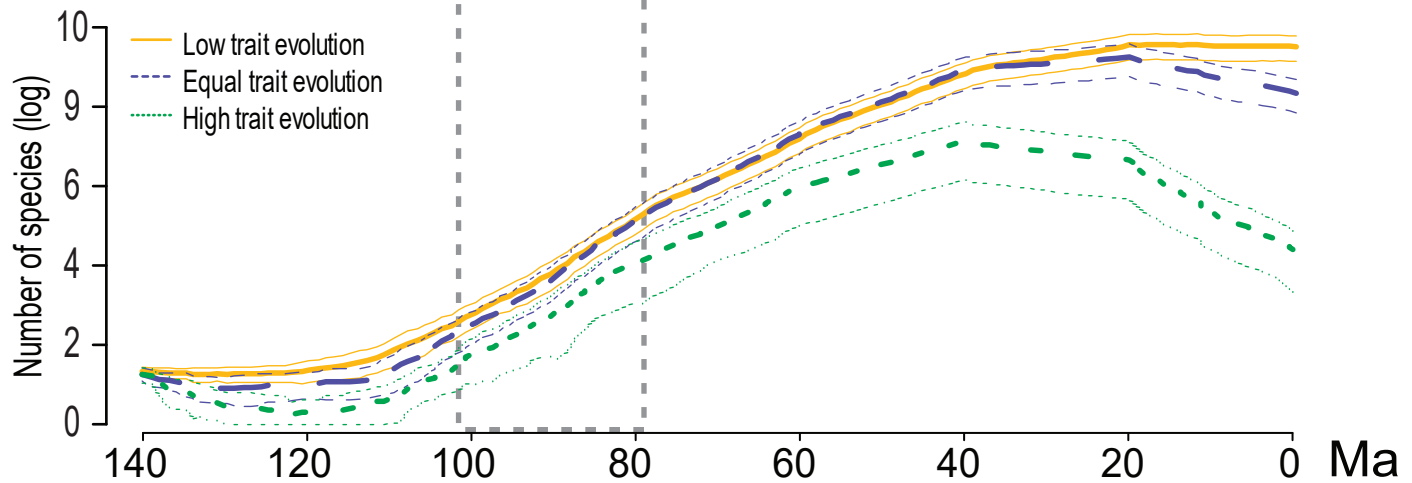

Supplement: S15 Fig — Results of the island case study showing (A) landscape size and environmental dynamics and (B) results of 3 experiments (i.e., lower, equal, and higher trait evolution compared with the temporal environmental variation). The time series in (B) shows γ richness (log10 scale) on theoretical oceanic islands, following the geomorphological dynamics of islands. Thick lines indicate the average of the replicates, whereas thin lines indicate SD envelopes (n = 30 for each trait evolutionary rate scenario). The dashed gray vertical bar crossing the entire plot indicates the period in which the island reaches its maximum size. Data presented available in S7 Data at https://zenodo.org/record/5006413. (PDF) [file pbio.3001340.s019.pdf]
