# Supplementary material for: gen3sis: A general engine for eco-evolutionary simulations of the processes that shape Earth’s biodiversity
Source: PLoS Biol. 2021 Jul 12;19(7):e3001340. doi: 10.1371/journal.pbio.3001340 (PMC8384074; doi:10.1371/journal.pbio.3001340)
Supplement: S6 Fig — Frequencies of Pearson correlation between simulated standardized mean species number per latitude (LDGcurve) against best matching empirical LDGcurve for each dynamic landscape L1 (in blue) and L2 (in pink) for models (A) M1, (B) M2, (C) M3, (D) M4, and (E) M5. Models M4 and M5 are the only ones producing correlations >0.5. Data presented available in S3 Data at https://zenodo.org/record/5006413. (PDF) [file pbio.3001340.s010.pdf]

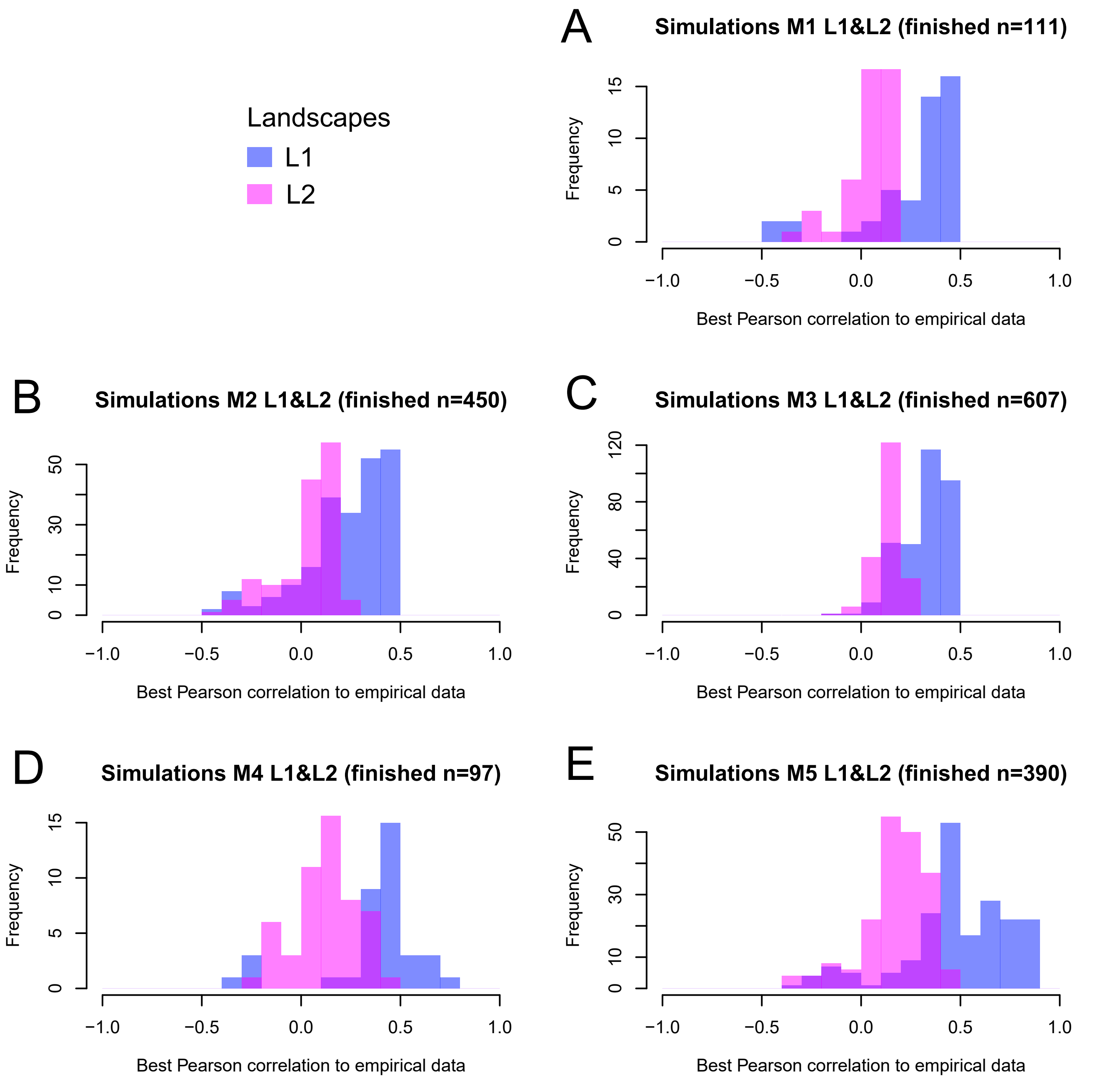

Landscapes
L1
L2
A
Simulations M1 L1&L2 (finished n=111)
Frequency
0
5
10
15
−1.0
−0.5
0.0
0.5
1.0
Best Pearson correlation to empirical data
B
Simulations M2 L1&L2 (finished n=450)
Frequency
0
10
30
50
C
Simulations M3 L1&L2 (finished n=607)
Frequency
0
40
80
120
D
Simulations M4 L1&L2 (finished n=97)
Frequency
0
5
10
15
E
Simulations M5 L1&L2 (finished n=390)
Frequency
0
10
30
50
