# Supplementary material for: gen3sis: A general engine for eco-evolutionary simulations of the processes that shape Earth’s biodiversity
Source: PLoS Biol. 2021 Jul 12;19(7):e3001340. doi: 10.1371/journal.pbio.3001340 (PMC8384074; doi:10.1371/journal.pbio.3001340)
Supplement: S8 Fig — Frequencies for models (A) M1, (B) M2, (C) M3, (D) M4, and (E)M5 with total frequency and frequency discriminated for each landscape, i.e., L1 and L2. Data presented available in S3 Data at https://zenodo.org/record/5006413. LDG, latitudinal diversity gradient. (PDF) [file pbio.3001340.s012.pdf]

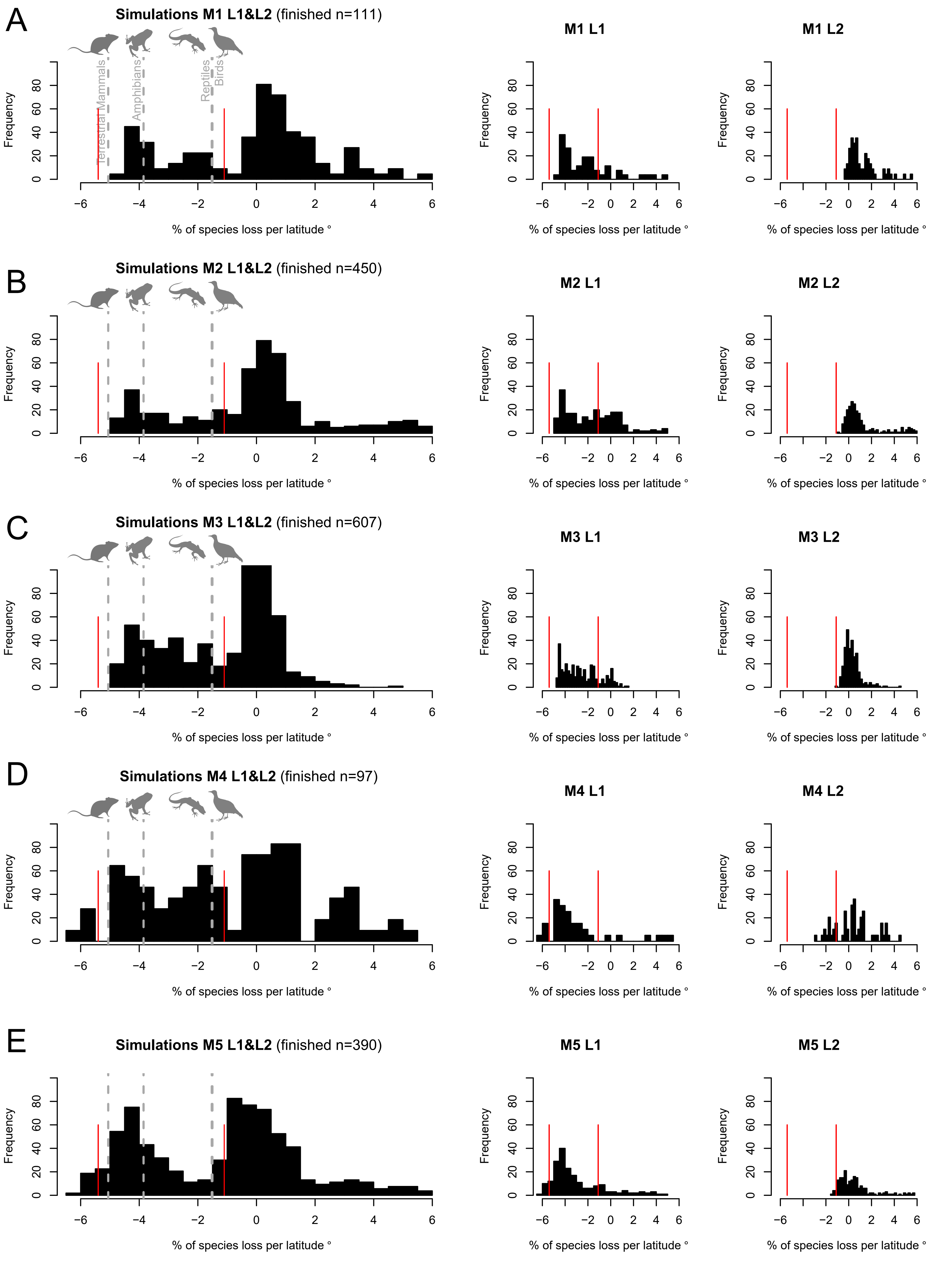

A
Simulations M1 L1&L2 (finished n=111)
Terrestrial Mammals
Amphibians
Reptiles
Birds
M1 L1
M1 L2
B
Simulations M2 L1&L2 (finished n=450)
M2 L1
M2 L2
C
Simulations M3 L1&L2 (finished n=607)
M3 L1
M3 L2
D
Simulations M4 L1&L2 (finished n=97)
M4 L1
M4 L2
E
Simulations M5 L1&L2 (finished n=390)
M5 L1
M5 L2
Frequency
% of species loss per latitude °
