# Supplementary material for: gen3sis: A general engine for eco-evolutionary simulations of the processes that shape Earth’s biodiversity
Source: PLoS Biol. 2021 Jul 12;19(7):e3001340. doi: 10.1371/journal.pbio.3001340 (PMC8384074; doi:10.1371/journal.pbio.3001340)
Supplement: S1 Note — (DOCX) [file pbio.3001340.s020.docx]

## Supporting Information Note S1

## Case Study: The emergence of the LDG in the Cenozoic.

### 1 Paleo-reconstructions

In order to evaluate the effects of landscapes on the conclusions related to large-scale biodiversity patterns, we compiled two landscapes (i.e. L1 and L2) using distinct methodologies and datasets. We reconstructed approximate estimations of surface temperatures and aridity for the Cenozoic (65 Ma until present) for the entire world based on two paleo-elevation models [1, 2] combined with lithologic and multiple other climate indicators at a spatial resolution of 1° and temporal resolution of ~170 kyr, i.e. six time steps per myr, following [3]. Below we present each landscape reconstruction in more detail.

#### 1.1 L1

For the first landscape L1 (Animation S1), paleogeographic digital elevation models providing paleotopography at a resolution of 1° × 1° and 5 myr temporal resolution were obtained from Scotese’s paleoatlas [2]. Paleotopographies were estimated by combining information on the dynamics of sea floor spreading, continental rift, subduction, continental collisions and other isostatic events influencing plate tectonics, together with other indicators of paleotopography and bathymetry [2]. We also used reconstructions of Köppen climatic zones at 5 myr temporal resolution compiled by the same author [3]. The basic Köppen classification for the present time depends on average monthly values of temperature and precipitation, and has five primary climatic zones corresponding to: tropical ever-wet, subtropical arid, warm temperate, cold temperate and polar. Reconstructions of the ancient Köppen zones are based on the geographic distribution of lithologic indicators of climate, including coal, evaporite, bauxite, tillite, glendonite, dropstones and other fossil evidence, such as high-latitude occurrences of palm, mangroves and alligators [4, 5]. A complete description of the sources of these lithologic indicators of climate can be found in [6]. The five principal Köppen climatic zones were drawn over twelve Cenozoic paleotopographic reconstructions according to the distribution of these lithologic indicators of climate. The average temperature of each of the modern Köppen zones was then calculated on the basis of present-day global temperature estimations. Modern temperatures served as the initial estimate of the temperature of each of the Köppen zones, which were then adjusted in order to match global mean temperature change over the Cenozoic [7]. Because the estimated Köppen zones correspond to discrete classes with hard boundaries, to accommodate a smoother latitudinal gradient we applied a focal analysis with a radius of 13° to smooth the temperature transition between each zone and mimic more realistic temperature transitions. The Köppen zones provide an estimate of the average surface temperature but do not account for topographic features. To account for the decrease in temperature with elevation, we computed the current temperature lapse rate (i.e. the rate of decrease in temperature with increasing elevation) for each Köppen zone based on the current digital elevation model and the WorldClim2 (WorldClim: 18 September 2018) annual mean temperature raster [8]. We applied the zone-specific lapse rate and linearly predicted the decrease in temperature with increasing elevation to obtain the final reconstruction of temperature at a temporal resolution of ~170 kyr for 65 Ma. The aridity index was computed based on the subtropical arid Köppen zone. Terrestrial surfaces covered by the subtropical arid zone had an aridity index of one, while all the other zones had a value of zero. We conducted a focal analysis with a radius of 13° to smooth the transition between arid and non-arid zones, approximating to the observed present gradient, followed by linear interpolation to achieve a final ~170 kyr temporal resolution. For the last 2.5 myr we approximated the minimum and maximum temperature peaks [9] to stress the amplitude of LDM oscillations within ~170 kyr time steps.

#### 1.2 L2

For the second landscape set L2 (Animation S2), paleogeographic digital elevation models providing paleotopography at a resolution of 1° × 1° and 1 myr temporal resolution were obtained from entirely different sources [1]. These include a new paleobathymetry model and variations due to mantle plume activity, updated regional plate kinematics, and models for the oceanic lithospheric age, sediment thickness, and reconstructed oceanic plateaus and microcontinents [1]. Moreover, we implemented a different approach to obtain temperature estimations than in landscape L1. We first reviewed the literature for studies estimating paleo-temperature variation along latitude using isotopes of marine surface benthic foraminifera [10-16]. For example, these studies estimated a temperature decrease of ~0.13°C per degree of latitude in the Late Palaeocene to Early Eocene (~55 Ma), ~0.33 C° per degree of latitude in the Oligocene (~34 Ma) and ~0.35°C per degree of latitude in the Pliocene (~ 5 Ma); they also provided estimations of absolute temperature at different latitudes. Specifically, these studies indicate that the gradient in temperature along latitude was flatter (0.13°C per degree of latitude) for periods with a warmer global climate (Eocene climate $\Delta t=+12^{\circ}C$) compared with the pre-industrial period with a cooler global climate (0.51°C per degree of latitude; $\Delta t=-0.3^{\circ}C$). These estimations from 65 to 2.5 Ma made it possible to compute an approximate empirical function linking temperature with latitude for a specific global temperature average. For a global temperature difference ($\Delta t)$ between the present and a given time step, the estimated temperature $t_{c}\left[ ^{\circ}∁ \right]$ of a single cell at a given latitude $l_{i}\left[ ^{\circ} \right]$ and temperature $t_{i}\left[ ^{\circ}C \right]$ was approximated with the function:

$$t_{c}= \left( t_{i}+\left( 0.8+0.004\times\left| l_{i} \right| \right) \right)\times\Delta t$$

Nevertheless, the temperature gradient along latitude is not a simple global linear decrease, and general current circulation reshapes the position of climate transitions [10]. Hence, to perform the mapping we also relied on the Köppen zones [3]. The average and the range of temperature of each of the modern Köppen zones was calculated on the basis of present-day global temperature estimations from the WorldClim2 (WorldClim: 18 September 2018) annual mean temperature raster, excluding cells at higher elevations [8]. We then attributed the temperature value of the past Köppen zones of each time step (i) within the range of present temperature values of those same Köppen zones, (ii) ensuring that the resulting temperature gradient corresponded closely to the latitudinal temperature gradient estimated above from [10-16]. To account for the decrease in temperature with elevation, we computed the current temperature lapse rate (i.e. the rate of decrease in temperature with increasing elevation) for each Köppen zone based on the current digital elevation model and the WorldClim2 (WorldClim: 18 September 2018) annual mean temperature raster [8, 17]. Surface temperatures were then interpolated at a ~170 kyr temporal resolution based on the most recent time series of global surface temperature for the Cenozoic [18]. This time series is based on a highly resolved, astronomically dated, continuous composite of benthic foraminifer isotope records [18]. In order to account for the Quaternary climatic oscillations, for the first 2.5 Ma a Last Glacial Maximum (LGM) surface temperature anomaly was applied to every second time step [19].

### 2. Model implementation

We used gen3sis to implement five hypotheses. In gen3sis, sites can be occupied by populations of different species, whose eco-evolutionary dynamics are driven by user-specified biological processes. Based on multiple processes (Fig 1 A, B), gen3sis computes ecological interactions, dispersal and evolution (Fig 1 G) and returns user-defined outputs (Fig 1 C–F). For instance, gen3sis simulates species’ population range dynamics, traits, diversification and spatial biodiversity patterns in response to geological, biological and environmental drivers. Using a combined trait-based and biological species concept, gen3sis tracks the dynamics of a species’ range, where each occupied site represents one population of the species. The abundance of species populations emerges from the combination of abiotic and biotic properties of the co-occurring species in the site. Species ranges are either grouped into clusters or segregated. Disconnected species population clusters that maintain geographic isolation for a prolonged period will result in different species after a predefined differentiation threshold is reached (modelling Dobzhansky-Muller incompatibilities; Dobzhansky 1982). For a pseudo-code of gen3sis see Note S3.

In gen3sis, the computation of all core processes can be modified through the configuration object. Consequently, gen3sis can account for a wide range of mechanisms given that multiple variables are handled by the engine. For example, (i) a non-constant speciation model could be made by having divergences depend on biotic and abiotic characteristics, and (ii) a more complex ecological model could be built by changing the *ecology* function. *Ecology* functions can account for more complex ecological equations [20], depending on traits such as specific niche optima, growth and metabolic rate. Additionally, It is possible to apply a trade-off in this function in order to upscale trade-off propositions [21]. Moreover, mechanisms can be completely turned off. In the case of ecology, this will lead to an ecologically neutral model. It is possible to expand the model to include ecological speciation as well, i.e. speciation as a consequence of trait divergence.

In this case study, we formulated five alternative hypotheses to explain the emergence of the LDG. Model M1, acting as a null model, had no *ecology* function and consequently no trait evolution process. We then implemented one hypothesis for each other group, i.e. niche evolution (model M2) variations in diversification rate (model M3) and ecological limits without (model M4) and with (model M5) temperature and aridity dependence. Model M2 followed the principle of niche conservatism, so that age of habitat positively influences species accumulation [22-27]. M1 speciation and dispersal mechanisms and M2 ecology and trait evolution mechanisms were the same for the other models if not stated otherwise. Model M3 had a speciation process three times faster in warmer regions, while models M4 and M5 had an energetic carrying capacity on the number of species and their abundances. Model M3 included the assumption that higher temperatures increase the speciation rate. It has been hypothesized that increases in metabolic and mutation rates, as well as a decrease in generation time, are linked with temperature and therefore should result in different speciation rates among species [28, 29]. Model M4 included the assumption that only area limits the total abundance of species. Model M5 included the additional assumption that the total resource amount scales with temperature and aridity and constrains the total abundance of species. It has been hypothesized that larger areas, as well as more solar radiation and greater water availability, all associated with the tropics, support more individuals and therefore more species [30-33]. Thus, the theory of carrying capacity proposes that energy limits the abundances of species and consequently the number of species that can coexist in a given place [33, 34].

Below we present the mechanism of core processes (i.e. speciation, ecology and evolution) implemented for the configuration objects of models M1, M2, M3, M4 and M5. Dispersal mechanisms are fully described in the main text. For a summary see Table A and for further modification examples see Note S2.

#### 2.1 Speciation

Within all models, speciation was set as entirely allopatric and happened for populations with a divergence threshold Ϟ of [6−60]. Thus, a speciation event happened if:

$${d'}_{i}>Ϟ$$

where ${d'}_{i}$ is the divergence value between populations. In all models but M3, the rate of divergence increase between isolated populations was kept constant and increased by ${d'}_{i}=d_{i}+1$ for every 170 kyr of isolation, where $d_{i}$ is the previous divergence value between populations. This represents, for configurations of M1, M2, M4 and M5, a speciation event after 1−10 myr of isolation, respectively for Ϟ of [6−60]. In configuration M3, we implemented simple temperature-dependent speciation [29] in which species in warmer environments accumulated stronger divergence between disconnected populations. The divergence increase of a certain species $d_{i}$ was given by:

$${d'}_{i}=d_{i}+\left( c+\left( \bar{t_{i}} \right)^{d_{power}} \right)$$

where $c$ is a constant, in this case fixed at 0.5, $\bar{t_{i}}$ is the arithmetic mean of the normalized species realized niche, and $d_{power}$ is the divergence temperature power and ranged from 2−6. This represents a rate of divergence of $d_{i}=+1.5$ for isolated populations of a species in the warmest locations and a rate of divergence of $d_{i}=+0.5$ for species populations in the coldest locations for every 170 kyr of isolation (Fig S1). This represents a speciation event three times faster for species in the consistently warmest and coldest sites with varying effect, i.e. linear to exponential.

#### 2.2 Ecology

In all models but M1 (without ecology), species $i$ abundance $a_{i}$ in a given site is provided by the following function:

$$a_{i}=1-|t_{opt_{i}}-t_{env}|$$

where $t_{opt_{i}}\left[ ^{\circ}∁ \right]$ is the optimal temperature of species $i$ and $t_{env}\left[ ^{\circ}C \right]$ is the site temperature. Note that $t_{opt_{i}}$ and $t_{env}$ are normalized to $[0,1]$ and thus$a_{i}$ is between zero and one. Species with $t_{opt_{i}}$ 90% further way from $t_{env}$ are locally extinct, i.e.:

$$a_{i}<0.1\overset{\Rightarrow}{then}a_{i}=0$$

The total abundance in a site ($A$) is defined as the sum of abundances acrossall *n* species $a_{i}$.

$$A=\sum_{i=1}^{n} a_{i}$$

Model M5 additionally has an energetic carrying capacity implemented. The carrying capacity $k$ determines the total maximum abundance allowed in a site, is dictated by site temperature $temp$, aridity $arid$ and the site area $area,$ and is power scaled by $k_{power}$, as determined by the following function:

$$k=area(temp\times(1-arid))^{k_{power}}$$

If a site total abundance is higher than $k$, i.e.$A>k$, low abundances (i.e. one) are removed progressively and randomly across the present species. This means that the stochastic vector *p* is the same for all $n$species with $a_{i}>0$.

$$p=[\frac{1}{n} \frac{1}{n} \cdots\frac{1}{n}]$$

This is repeated until site abundance $A'$ is lower than $k$, i.e $A'\leq k$, possibly leading to local extinctions. Species that are more fit for the environment (i.e. $t_{opt_{i}}\sim t_{env}$) consequently have higher abundances and therefore are less susceptible to local extinctions, i.e. $a_{i}\to1$. Thus species $i$ has a larger buffer for the abundance reductions in case of $A'>k$ because $p_{i}=\frac{1}{n}$ . The final abundances should always be equal to or smaller than the carrying capacity.

$$A<k\overset{\Rightarrow}{then}A=A$$

$$A\geq k\overset{\Rightarrow}{then}A=A'$$

#### 2.3 Trait evolution

In all models but M1 (without trait evolution), the *evolution* function first applies a trait homogenization for each cluster of each species $i$. Specifically, temperature optimum traits ${t_{opt}}_{i}$ were updated to ${{t'}_{opt}}_{i}$ after ecological processes. Each geographically clustered population $n$ was homogenized by an abundance-weighted mean, weighting traits based on a population abundance $p_{i}$, which mimics the influence of sharing and selection of the most favourable trait variations within a geographic cluster.

$${{t'}_{opt}}_{i}={t_{opt}}_{i}\times\frac{p_{i}}{\frac{\sum_{i=1}^{n} p_{i}}{n}}$$

Then, the temperature optimum of each population ${t'}_{opt}$ was set to evolve randomly, following a normal distribution in a Brownian motion fashion. For each time step, the temperature optimum (${t''}_{opt}$) was increased or reduced by a normally distributed value with mean zero and standard deviation ($\sigma^{2})$:

$${t''}_{opt}={t'}_{opt}+N (0,\sigma^{2})$$

We explored $\sigma^{2}$ values of [0.001−0.010], which represent $\sigma^{2}$ values of [0.1°C−1°C] on a temperature scale [-45−55°C].

In our case study, we present a few possible mechanisms associated with five main groups of LDG hypotheses [35], but these are not the only ways of implementing these hypotheses. Further studies could investigate the calculation of maximum abundances per site in the *ecology* function or the use of different functions for the diversification rate.

#### 2.4 Overview M1, M2, M3, M4 and M5

For an overview of the implemented processes and explored parameters used in the case study, see Table A.

**Table A**. Core processes and explored parameters of the NULL model M1, and alternative models M2, M3, M4 and M5. We present the explored parameters used for the simulations. Simulations were initialized with one single ancestor species spread over the entire terrestrial surface of the Earth at 65 Ma, where the temperature optimum of each population matched local site conditions.

|  |  | Core processes | | | |
| --- | --- | --- | --- | --- | --- |
|  |  | Speciation | Dispersal | Evolution | Ecology |
| Models | **M1**  Null model without ecology and trait evolution | Divergence increase  ${d'}_{i}=d_{i}+1$  Speciation event if  ${d'}_{i}>Ϟ$ | Weibull distributed with shape (ɸ) and scale (Ψ).  $W(ɸ,\Psi)$ | No traits defined | No ecological interactions defined |
|  |  | Ϟ=[6,60] | ɸ=[2,5];Ψ=[550,850] |  |  |
|  | **M2**  Temperature and aridity niches evolving, niche conservatism | Same as  in M1 | Same as  in M1 | Trait homogenization ${t'}_{opt}=t_{opt}$  Trait change   ${t''}_{opt}={t'}_{opt}+ N(0,\sigma^{2})$ | Abundance update  $A=\sum_{i=1}^{n} \left( 1-\vert t_{opt_{i}}-t_{env}\vert\right)$ |
|  |  |  |  | $\sigma^{2}$=[0.001,0.01] |  |
|  | **M3**  Faster speciation rates in warmer niches | Divergence increase  ${d'}_{i}=d_{i}+\left( c+\left( \bar{t_{i}} \right)^{d_{power}} \right)$  Speciation event if  ${d'}_{i}>Ϟ$  $d_{power}$=[2,6]; Ϟ=[6, 60] | Same as  in M1 | Same as  in M2 | Same as  in M2 |
|  | **M4**  Environmentally independent carrying capacity for abundance |  | Same as  in M1 | Same as  in M2 | Abundance update  same as in M2  Carrying capacity  $k={area}^{k_{power}}$  If $A>k$,  $A^{'}=k$   $k_{power}$=[1,4] |
|  | **M5**  Energetic carrying capacity for abundance | Same as  in M1 | Same as  in M1 | Same as  in M2 | Abundance update  same as in M2  Carrying capacity  $k=area\times$  $(temp\times(1-arid))^{k_{power}}$  If $A>k$,  $A^{'}=k$   $k_{power}$=[1,4] |

### 3. Empirical data

#### 3.1 Distribution

Following [36], global distribution maps of known extant mammals (5,289 species), birds (10,065 species), reptiles (4,278 species) and amphibians (6,309 species) were generated from polygons obtained from the IUCN Red lists [37, 38] and from BirdLife International for birds [39]. This resulted in a total of 25,941 species with spatial information.

#### 3.2 Phylogenies

Following [40], we used the fossil-calibrated molecular phylogenies for mammals (5,020 species) [41], modified by [42], birds (6,670 species) [43], squamates (4,162 species) [44] and amphibians (3,126 species) [45]. This meant a total of 18,978 species with phylogenetic data.

### 4. Model Analysis

Parameters were initially based on prior knowledge and conservative estimations [46, 47]. We explored dispersal distributions and parameters ranging in realized mean and 95% quantile from less than a single cell (i.e. ~50 km^2^ for a landscape at 4°) to more than the Earth’s diameter (i.e. ~12,742 km^2^). For this, we used multiple shapes ɸ and scales Ψ of a Weibull distribution (Fig S2). This allowed us to explore multiple shapes of distribution, e.g. a shape of one represents an exponential distribution. We initially explored ɸ values between 0.5 and 5 and Ψ values between 100 and 1000. Simulations with low shape values (i.e. ɸ=0.5 and 1), approximating an exponential distribution, had longer tails and many unrealistic dispersal patterns. Trait evolution frequency and intensity ranged from zero to one and speciation thresholds ranged from 1 to 60 (i.e. 170 kyr to 10 myr). Viable parameters related to ecology and speciation were the ones that resulted in simulations that had: (i) at least 20 speciation events; (ii) not all species becoming extinct; (iii) fewer than 50,000 species; or (iv) fewer than 10,000 species cohabiting the same site at any point in time.

We explored model parameters using Sobol sequences, a quasi-random number generator that samples parameters evenly across the parameter space [48]. We used continuous ranges for each parameter, basing upper and lower parameter boundaries on the literature and interactive modelling explorations (Fig 3). We then did a full factorial parameter exploration of the viable parameters for all models (i.e. M1, M2, M3, M4 and M5) and landscapes (i.e. L1, L1.0, L2 and L2.0) at a coarse resolution of 4° (i.e. M1 n=300, M2 n=780, M3 n=1020, M4 n=300, M5 n=780). M1, M2, M3, M4 and M5 had 198 (66%), 536 (69%), 673 (66%), 181(60%) and 480 (62%) complete simulations, respectively, meaning that they did not reach the maximum number of total species and that all species did not go completely extinct. This shows that we were conservative with our previous parameter range exercise. Of these viable simulations, M1, M2, M3, M4 and M5 had 68 (34%), 234 (44%), 418 (62%), 98 (54%) and 300 (62%) simulations, respectively, with negative LDG slopes, representing a decrease in richness with increasing latitude (for LDG curve comparisons of best matching simulations see Fig S5). When considering the acceptance threshold from empirical evidence, these numbers reduced proportionally (Table 3 in the main text). We observed two distinct peaks in the predicted LDG related to the landscapes considered, with the L1 peak occurring close to that in the empirical LDG of terrestrial mammals and amphibians and the L2 peak approaching the empirical LDG of terrestrial reptiles and birds (Fig S8). This stresses the relevance of multiple (and ideally reliable) paleoenvironmental reconstructions in quantifying uncertainties in eco-evolutionary simulations derived from landscape data input.

A correlation analysis between all model parameters and emerging large-scale biodiversity patterns was conducted (Table B, Figures S13 and S14). In all models, dispersal shape was negatively correlated with LDG_%loss_, i.e. M1 (cor=-0.08), M2 (cor=-0.13), M3 (cor=-0.17), M4 (cor=-0.1) and M5 (cor=-0.01). Decreasing shape values resulted in longer tails, thus increasing long-distance dispersal events (Fig S2). These results reinforce findings from previous studies, where dispersal ability was also negatively correlated with LDG [23]. Moreover, with the exception of M3 (cor=0.02), LDG was negatively correlated with divergence threshold, i.e. M1 (cor=-0.07), M2 (cor=-0.11), M4 (cor=-0.08) and M5 (cor=-0.11). The temperature-dependent speciation mechanism in M3 was expected to weaken this relationship compared with in the other models. Temperature-dependent divergence and carrying-capacity power were correlated weakly but positively with LDG in M3 (cor=0.04) and M2 (cor=0.15). Correlations with tree imbalance followed similar trends as LDG, with the distinction that the divergence thresholds of M3 were correlated positively with tree imbalance (cor=0.13) (Table B). Correlations were consistent across landscapes (Figures S13 and S14).

Our results show that an increase in k resulted in a steeper LDG slope, which is in agreement with [33, 34, 49], who proposed that ecological limits represent the main determinant of the LDG. An increasing divergence threshold, leading to lower species richness, flattened the LDG slope so that the tropics accumulated diversity more slowly. For information on the significance of mean parameter contributions to negative and positive LDGs see Table C.

**Table B**. Correlations between M1, M2, M3, M4 and M5 model parameters and emerging patterns, i.e. β value and species loss per latitude (LDG). Dispersal-related parameters are the shape ɸ and scale Ψ of a Weibull distribution. The speciation-related parameter is the divergence threshold Ϟ, and the evolution-related parameter is the trait standard deviation change σ^2^. Specific to M3 and related to speciation is temperature-divergence power *d_power_*. Specific to M4 and M5 and related to the *ecology* function is the carrying-capacity power $k_{power}$.

|  | Emerging patterns | | | | | | | | | |
| --- | --- | --- | --- | --- | --- | --- | --- | --- | --- | --- |
| Parameters | M1 | | M2 | | M3 | | M4 | | M5 | |
|  | β | LDG | β | LDG | β | LDG | β | LDG | β | LDG |
| ɸ | -0.03 | -0.08 | 0.01 | -0.13 | -0.1 | -0.17 | -0.07 | -0.1 | 0.06 | -0.01 |
| Ψ | -0.12 | 0.19 | -0.03 | 0.12 | -0.09 | 0.23 | -0.03 | 0.11 | -0.08 | 0.03 |
| Ϟ | -0.45 | 0.07 | -0.39 | 0.11 | −0.24 | -0.02 | -0.36 | 0.08 | -0.19 | 0.11 |
| σ^2^ |  |  | 0 | 0.01 | 0.04 | 0.02 | -0.18 | -0.16 | -0.02 | 0.03 |
| *d_power_* |  |  |  |  | 0 | 0.01 |  |  |  |  |
| *k_power_* |  |  |  |  |  |  | 0.07 | 0.15 | 0.07 | -0.18 |

**Table C**. Significance of mean parameter contributions to negative and positive LDGs. Shown are the p-values of unpaired two-sample Wilcoxon tests for the models and parameters between simulations with positive and negative LDGs.

|  | Models | | | | |
| --- | --- | --- | --- | --- | --- |
| Parameters | M1 | M2 | M3 | M4 | M5 |
| ɸ ***** | 5.01E-01 | **3.96E-03** | 7.57E-01 | **3.82E-01** | 8.12E-01 |
| Ψ | 2.12E-01 | 2.78E-01 | **4.82E-09** | **5.42E-02** | 3.15E-01 |
| Ϟ | 5.65E-01 | 6.06E-01 | **4.12E-02** | 2.76E-01 | 1.86E-01 |
| σ^2^ |  | 8.74E-01 | 8.73E-01 | **7.05E-02** | 3.99E-01 |
| *d_power_* |  |  | **3.22E-02** |  |  |
| *k_power_****** |  |  |  | 9.68E-01 | **1.30E-05** |

**Table D**. Percentage of simulations with a positive species loss per latitudinal degree (i.e. LDG_%loss_ > 0) and relative contributions of landscapes L1 and L2 for models M1, M2, M3, M4 and M5.

|  | Simulations | Contributions | |
| --- | --- | --- | --- |
|  | LDG_%loss_ > 0 | L1 | L2 |
| M1 | 34% | 89% | 11% |
| M2 | 44% | 79% | 21% |
| M3 | 62% | 73% | 27% |
| M4 | 54% | 58% | 42% |
| M5 | 62% | 68% | 32% |

#### 4.1 Large-scale biodiversity patterns

LDG was calculated as the percentage of species loss per latitudinal degree (LDG_%loss_). This is given by the slope of a linear regression of normalized species richness (i.e. intercept always 0 and highest richness always 1) and latitude, multiplied by 100.

$$LDG\%=\left( \frac{y_{i}}{\left| x_{i} \right|} \right) \times100$$

for *n* data pairs and where $y_{i}$ are richness values and $x_{i}$ are latitude values for $i=1,\ldots,n$. The absolute value of latitude was used in order to consider north and south gradients equally.

The spatial distribution of biodiversity data was calculated by a Pearson correlation of normalized α-biodiversity values using the function ‘cor.test’ from the stats R-package *stats* [50] across simulations and each empirical dataset.

Ranges were calculated based on the area occupied by a species in km^2^. Distributions were evaluated based on a consistent decrease in the number of large-range species, with a tolerance of 5%. Since species range histograms are left-skewed [51-53], we evaluated whether simulated species range histograms decreased from left to right, with a 5% tolerance for each break.

For the phylogenetic tree imbalance evaluation, we only used ultrametric trees converted with the function ‘drop.fossil’ from the R-package *ape* [54]. We then calculated the value that maximizes the likelihood in the β-splitting model [55] using the function ‘maxlik.betasplit’ (*up*=10) from the R-package *apTreeshape* [56].

For the temporal dynamics of species diversification, we matched and computed differences between empirical and simulated normalized lineage though time (nLTT) using the function ‘nltts_diff’ available at the R-package *nLTT* [57].

#### 4.2 Comparison between model output/results and empirical data

The POM approach requires the specification of a range for each pattern under which observation and prediction are acceptably close. This means that the simulation reproduces the empirical observation to an acceptable degree. Unless POM is coupled with an explicit probabilistic model [58], the limits for acceptance must be decided by the modeller based on their understanding of the data [46, 47]. We used the following acceptance criteria: (i) LDG between 5.4% and 1.1%, (ii) α-diversity Pearson correlation > 0.4, (iii) range distribution with a decrease in the number of large-range species, with a tolerance of 5%, (iii) β between -1.4 and -0.3, and (iv) nLTT differences < 0.15.

Empirical values of LDG_%loss_ and model r^2^ and β were: mammals (LDG_%loss_ =5.1%, r^2^=0.89, β=-0.4), birds (LDG_%loss_ =1.5%, r^2^=0.92, β=-1.3), amphibians (LDG_%loss_ =3.9%, r^2^=0.89, β=-0.7) and reptiles (LDG_%loss_ =1.5%, r^2^=0.97, β=-0.8). As expected [51-53], all major tetrapod groups had range distributions with a decrease in the number of large-range species.

M5 had a higher proportion of simulations with a decrease in LDG (Table D). M5 gained support when the LDG and phylogenetic tree imbalance were considered together (i.e. M2=3%, M3=3% and M5=6% of accepted simulations). However, this pattern was strongly associated with the landscape used, with L1 and L2 resulting in 47% and 20%, respectively, of the LDG that corresponded to a decrease in species richness per latitude in M5 (Fig S8). This pattern was consistent for all models, stressing the relevance of alternative and reliable paleo-reconstructions for answering key large-scale biodiversity patterns (Table D).

#### 4.3 Model selection

For the best model among all simulations, we additionally examined the current α-diversity of the multiple empirical distributions (Fig S9). We compared observed and predicted species richness using a simple regression model from which we extracted the coefficient of a Pearson correlation for finished and satisfactory simulations, i.e. r > 0.4. Because models differed in the number of estimated parameters, we also computed the Bayesian information criterion (BIC) of each simulation, following [59]:

$$BIC=\log\left( n \right)k+log(\frac{RSS}{n})n$$

where RSS represents the difference between scaled observed and predicted site values, n the number of samples and k the number of model parameters. For each model, we summed the BIC in histograms along 2D Kernel density estimates of ordinary parameters (Fig S3).

#### 4.4 Macro-evolutionary dynamics

Speciation, extinction and dispersal events emerge as consequences of multiple interacting processes. We therefore quantified speciation and extinction for every 1 myr interval and determined the final contribution of dispersal events for torrid [23° 27' N−23° 27' S], temperate [66° 33' N−23° 27' N] and [23° 27' S−66° 33' S] and polar [0° N−66° 33' N and 66° 33' S−90° S] regions for each model, with and without temporal environmental dynamics. We selected the seven best-matching simulations, regarding the α-diversity Pearson correlation, for each model and category. Mean absolute speciation and extinction rates differed strongly across scenarios of temporal dynamics and slightly across models (Fig S10). Standardized speciation (Fig S11) and extinction events (Fig S12) are shown separately for each simulation. The region of speciation or extinction was determined by the location of the appearance or disappearance of a species. For example, if a speciation event occurred in a species occupying multiple regions, the region of speciation was attributed to the region with the most occurrences. If multiple regions were occupied without a single region with more than 50% of the occurrences, the individual region was defined as multiple.

The proportional contribution of dispersion for the main latitude regions was calculated as the % of immigrants from multiple regions currently present in a region (Tables S5 and S6). Thus, dispersal events were based on the proportion of species originating in a region and dispersing to destination regions without (Table E) and with (Table F) deep-time environmental dynamics.

**Table E**. Dispersal contributions in landscapes without deep-time dynamics. The simulated proportional contribution of species dispersal events in landscapes L1.0 and L2.0 is shown for all models and for each main latitudinal region. The average simulated proportional contribution of species dispersal events from all main latitudinal regions, as well as the standard deviation, is given for the simulations. The percentages (%) of species contributions originating in (column) and dispersed to the destination regions (rows) are shown.

|  |  |  | Zone of origin (from) | | | |
| --- | --- | --- | --- | --- | --- | --- |
|  |  |  | Torrid | Temperate | Polar | Multiple |
| Zone of destination (to) | M1 | Torrid | 58% ± 21% | 26% ± 10% | 15% ± 15% | 1% ± 2% |
|  |  | Temperate | 50% ± 17% | 35% ± 6% | 15% ± 13% | 0% ± 0% |
|  |  | Polar | 30% ± 9% | 36% ± 8% | 34% ± 3% | 0% ± 0% |
|  |  | Multiple | 89% ± 15% | 13% ± 0% | 8% ± 0% | 0% ± 0% |
|  | M2 | Torrid | 62% ± 20% | 18% ± 11% | 18% ± 15% | 4% ± 4% |
|  |  | Temperate | 48% ± 20% | 34% ± 16% | 17% ± 10% | 1% ± 1% |
|  |  | Polar | 30% ± 7% | 40% ± 8% | 31% ± 9% | 0% ± 0% |
|  |  | Multiple | 78% ± 7% | 12% ± 0% | 6% ± 0% | 27% ± 0% |
|  | M3 | Torrid | 53% ± 19% | 25% ± 11% | 24% ± 8% | 4% ± 2% |
|  |  | Temperate | 43% ± 12% | 29% ± 8% | 32% ± 15% | 35% ± 21% |
|  |  | Polar | 30% ± 4% | 39% ± 11% | 41% ± 27% | 0% ± 0% |
|  |  | Multiple | 0% ± 0% | 0% ± 0% | 0% ± 0% | 0% ± 0% |
|  | M4 | Torrid | 58% ± 17% | 29% ± 9% | 12% ± 12% | 3% ± 2% |
|  |  | Temperate | 49% ± 14% | 37% ± 9% | 13% ± 10% | 2% ± 2% |
|  |  | Polar | 39% ± 06% | 32% ± 4% | 29% ± 8% | 0% ± 0% |
|  |  | Multiple | 51% ± 15% | 39% ± 22% | 3% ± 1% | 11% ± 2% |
|  | M5 | Torrid | 58% ± 25% | 27% ± 13% | 17% ± 12% | 1% ± 1% |
|  |  | Temperate | 51% ± 13% | 35% ± 07% | 14% ± 8% | 1% ± 1% |
|  |  | Polar | 32% ± 5% | 36% ± 14% | 37% ± 7% | 0% ± 0% |
|  |  | Multiple | 48% ± 3% | 26% ± 14% | 25% ± 20% | 10% ± 6% |

**Table F**. Dispersal contributions in landscapes with deep-time dynamics. The simulated proportional contribution of species dispersal events in landscapes L1 and L2 is shown for all models and for each main latitudinal region. The average simulated proportional contribution of species dispersal events from all main latitudinal regions, as well as the standard deviation, is given for the simulations. The percentages (%) of species contributions originating in (column) and dispersed to the destination regions (rows) are shown.

|  |  |  | Zone of origin (from) | | | |
| --- | --- | --- | --- | --- | --- | --- |
|  |  |  | Torrid | Temperate | Polar | Multiple |
| Zone of destination (to) | M1 | Torrid | 89% ± 3% | 7% ± 2% | 2% ± 1% | 2% ± 0% |
|  |  | Temperate | 57% ± 19% | 33% ± 17% | 09% ± 5% | 13% ± 10% |
|  |  | Polar | 62% ± 41% | 49% ± 15% | 34% ± 22% | 0% ± 0% |
|  |  | Multiple | 1% ± 0% | 1% ± 0% | 0% ± 0% | 0% ± 0% |
|  | M2 | Torrid | 89% ± 6% | 10% ± 2% | 4% ± 2% | 3% ±% 1% |
|  |  | Temperate | 67% ± 18% | 38% ± 2% | 13% ± 3% | 33% ± 0% |
|  |  | Polar | 10% ± 0% | 10% ± 0% | 0% ± 0% | 0% ± 0% |
|  |  | Multiple | 0% ± 0% | 0% ± 0% | 0% ± 0% | 0% ± 0% |
|  | M3 | Torrid | 91% ± 4% | 7% ± 3% | 3% ± 2% | 1% ± 0% |
|  |  | Temperate | 45% ± 17% | 36% ± 14% | 1% ± 6% | 13% ± 5% |
|  |  | Polar | 38% ± 0% | 38% ± 0% | 25% ± 0% | 0% ± 0% |
|  |  | Multiple | 0% ± 0% | 0% ± 0% | 0% ± 0% | 0% ± 0% |
|  | M4 | Torrid | 92% ± 2% | 5% ± 2% | 2% ± 1% | 2% ± 1% |
|  |  | Temperate | 72% ± 11% | 16% ± 8% | 8% ± 5% | 7% ± 4% |
|  |  | Polar | 72% ± 33% | 19% ± 1% | 58% ± 19% | 0% ± 0% |
|  |  | Multiple | 7% ± 34% | 67% ± % | 0% ± 0% | 22% ± 0% |
|  | M5 | Torrid | 76% ± 12% | 11% ± 4% | 11% ± 7% | 2% ± 2% |
|  |  | Temperate | 54% ± 21% | 27% ± 9% | 16% ± 12% | 5% ± 4% |
|  |  | Polar | 44% ± 23% | 48% ± 22% | 34% ± 19% | 0% ± 0% |
|  |  | Multiple | 46% ± 12% | 29% ± 14% | 31% ± 5% | 3% ± 2% |

#### 4.5 Spatial resolution

As an illustration of the effects of spatial scale on the selected mode, we ran simulations at higher resolution for a subset of the models and parameters (n=12). We increased the paleolandscape spatial resolution from 4° to 1° in M2 in L1 (Fig 4). We observed that, for the subset of simulations analysed, increasing the spatial resolution of the simulations resulted in a slight decrease in the LDG and, as expected, an increase in γ richness and computation time (Fig S7). This expected artefact relating to resolution [60] can also be linked with the disproportionally larger number of sites towards higher latitudes, which also affects population connectivity and therefore speciation rates. Moreover, increasing the simulation resolution from 4° to 1° resulted, as expected, in an increase in the absolute number of species (γ richness) and CPU time (Fig S7). There was a trend of decreasing LDG slope with increasing resolution, possibly explained by the larger amount of spatial heterogeneity included, as well as complex interactions between $k$, $A$ and $a_{i}$, which are $area$ dependent. Moreover, the decrease in the LDG slope could be explained by the assumptions of non-area effects on dispersal and mutations occurring during sampling routines, which deserve further investigation. Future studies investigating variable spatial and temporal resolution in combination with biological complexity are necessary in order to reveal processes.

From a selected simulation of M6 in L1 at 1°(n=12) that predicted realistic biodiversity patterns, e.g. higher species richness in tropical regions (Fig 4, Animation S4), we removed the sites without any species in the empirical data. Simulated and empirical richness were normalized and the Pearson correlation was calculated (Fig S9). Our simulation predicted higher species richness in tropical regions (Fig 4, Animation S4), matching the spatial patterns of empirical species richness for mammals (Pearson r=0.6), birds (r=0.57), amphibians (r=0.57) and reptiles (r=0.38). Simulated phylogeny had a node distribution that was denser closer to the present and had a rather balanced tree shape (β=0.012) [61]. The emerging LDG (i.e. 4.6% of species loss per latitudinal degree) closely matched empirical curves (Fig 4C). An LDG mismatch towards the poles was expected, since we did not consider continental ice sheets in our landscapes. We decided not to account for currently ice-covered sites because past ice coverage reconstructions were not available for the studied interval. Moreover, LDG inflation towards the poles was expected as a result of similar artefacts, as observed when increasing spatial resolution.

### 5. References

1. Straume EO, Gaina C, Medvedev S, Nisancioglu KH. Global Cenozoic Paleobathymetry with a focus on the Northern Hemisphere Oceanic Gateways. Gondwana Research. 2020;86:126-43. doi: 10.1016/j.gr.2020.05.011.

2. Scotese CR, Wright N. PALEOMAP Paleodigital Elevation Models (PaleoDEMS) for the Phanerozoic. 2018. Available from: https://www.earthbyte.org/paleodem-resource-scotese-and-wright-2018.

3. Hagen O, Vaterlaus L, Albouy C, Brown A, Leugger F, Onstein RE, et al. Mountain building, climate cooling and the richness of cold‐adapted plants in the Northern Hemisphere. J Biogeogr. 2019. doi: 10.1111/jbi.13653.

4. Boucot AJ, Xu C, Scotese CR, Morley RJ. Phanerozoic paleoclimate: an atlas of lithologic indicators of climate. 2013.

5. Scotese CR. Some thoughts on global climate change: the transition from icehouse to hothouse. Paleomap project. 2015;21:1 (2).

6. Boucot AJ, Xu C, Scotese CR, Morley RJ. Phanerozoic paleoclimate: an atlas of lithologic indicators of climate. Tulsa, U.S.A.: Society of Economic Paleontologists and Mineralogists (Society for Sedimentary Geology); 2013.

7. Royer DL, Berner RA, Montañez IP, Tabor NJ, Beerling DJ. CO2 as a primary driver of Phanerozoic climate. GSA Today. 2004;14(3). doi: 10.1130/1052-5173(2004)014<4:Caapdo>2.0.Co;2.

8. Fick SE, Hijmans RJ. WorldClim 2: new 1-km spatial resolution climate surfaces for global land areas. International Journal of Climatology. 2017;37(12):4302-15.

9. Bradley RS. Paleoclimatology: reconstructing climates of the Quaternary. 2 ed. San Diego: Elsevier; 1999.

10. Zhang L, Hay WW, Wang C, Gu X. The evolution of latitudinal temperature gradients from the latest Cretaceous through the Present. Earth-Sci Rev. 2019;189:147-58. doi: 10.1016/j.earscirev.2019.01.025.

11. Hollis CJ, Dunkley Jones T, Anagnostou E, Bijl PK, Cramwinckel MJ, Cui Y, et al. The DeepMIP contribution to PMIP4: methodologies for selection, compilation and analysis of latest Paleocene and early Eocene climate proxy data, incorporating version 0.1 of the DeepMIP database. Geoscientific Model Development. 2019;12(7):3149-206. doi: 10.5194/gmd-12-3149-2019.

12. Hutchinson DK, de Boer AM, Coxall HK, Caballero R, Nilsson J, Baatsen M. Climate sensitivity and meridional overturning circulation in the late Eocene using GFDL CM2.1. Climate of the Past. 2018;14(6):789-810. doi: 10.5194/cp-14-789-2018.

13. Evans D, Sagoo N, Renema W, Cotton LJ, Muller W, Todd JA, et al. Eocene greenhouse climate revealed by coupled clumped isotope-Mg/Ca thermometry. Proceedings of the National Academy of Sciences of the United States of America. 2018;115(6):1174-9. Epub 2018/01/24. doi: 10.1073/pnas.1714744115.

14. Cramwinckel MJ, Huber M, Kocken IJ, Agnini C, Bijl PK, Bohaty SM, et al. Synchronous tropical and polar temperature evolution in the Eocene. Nature. 2018;559(7714):382-6. Epub 2018/07/04. doi: 10.1038/s41586-018-0272-2.

15. Sijp WP, von der Heydt AS, Dijkstra HA, Flögel S, Douglas PMJ, Bijl PK. The role of ocean gateways on cooling climate on long time scales. Global Planet Change. 2014;119:1-22. doi: 10.1016/j.gloplacha.2014.04.004.

16. Keating-Bitonti CR, Ivany LC, Affek HP, Douglas P, Samson SD. Warm, not super-hot, temperatures in the early Eocene subtropics. Geology. 2011;39(8):771-4. doi: 10.1130/g32054.1.

17. Basher Z, Bowden DA, Costello MJ. Global Marine Environment Datasets (GMED) World Wide Web2018 [cited 2020 15.10.2020]. Available from: http://gmed.auckland.ac.nz.

18. Westerhold T, Marwan N, Drury AJ, Liebrand D, Agnini C, Anagnostou E, et al. An astronomically dated record of Earth’s climate and its predictability over the last 66 million years. Science. 2020;369(6509):1383-7. doi: 10.1126/science.aba6853.

19. Annan JD, Hargreaves JC. A new global reconstruction of temperature changes at the Last Glacial Maximum. Climate of the Past. 2012;9(1):367-76. doi: 10.5194/cp-9-367-2013.

20. Pontarp M, Wiens JJ. The origin of species richness patterns along environmental gradients: uniting explanations based on time, diversification rate and carrying capacity. J Biogeogr. 2017;44(4):722-35. doi: 10.1111/jbi.12896.

21. Pellissier L, Descombes P, Hagen O, Chalmandrier L, Glauser G, Kergunteuil A, et al. Growth‐competition‐herbivore resistance trade‐offs and the responses of alpine plant communities to climate change. Funct Ecol. 2018;32(7):1693-703. doi: 10.1111/1365-2435.13075.

22. Silvestro D, Castiglione S, Mondanaro A, Serio C, Melchionna M, Piras P, et al. A 450 million years long latitudinal gradient in age-dependent extinction. Ecol Lett. 2020;23(3):439-46. Epub 2019/12/20. doi: 10.1111/ele.13441.

23. Saupe EE, Myers CE, Townsend Peterson A, Soberon J, Singarayer J, Valdes P, et al. Spatio-temporal climate change contributes to latitudinal diversity gradients. Nature Ecology & Evolution. 2019;3(10):1419-29. Epub 2019/09/11. doi: 10.1038/s41559-019-0962-7.

24. Saupe EE, Myers CE, Peterson AT, Soberón J, Singarayer J, Valdes P, et al. Non‐random latitudinal gradients in range size and niche breadth predicted by spatial patterns of climate. Global Ecol Biogeogr. 2019;28(7):928-42. doi: 10.1111/geb.12904.

25. Rangel TF, Edwards NR, Holden PB, Diniz-Filho JAF, Gosling WD, Coelho MTP, et al. Modeling the ecology and evolution of biodiversity: Biogeographical cradles, museums, and graves. Science. 2018;361(6399):eaar5452. doi: 10.1126/science.aar5452.

26. Tittensor DP, Worm B. A neutral-metabolic theory of latitudinal biodiversity. Global Ecol Biogeogr. 2016;25(6):630-41. doi: 10.1111/geb.12451.

27. Smith BT, Seeholzer GF, Harvey MG, Cuervo AM, Brumfield RT. A latitudinal phylogeographic diversity gradient in birds. PLoS Biol. 2017;15(4):e2001073. doi: 10.1371/journal.pbio.2001073.

28. Allen AP, Gillooly JF, Savage VM, Brown JH. Kinetic effects of temperature on rates of genetic divergence and speciation. Proceedings of the National Academy of Sciences of the United States of America. 2006;103(24):9130-5. doi: 10.1073/pnas.0603587103.

29. Rohde K. Latitudinal gradients in species diversity: the search for the primary cause. Oikos. 1992:514-27.

30. MacArthur RH, Wilson EO. An Equilibrium Theory of Insular Zoogeography. Evolution. 1963;17(4):373-87. doi: 10.2307/2407089.

31. Rosenzweig ML. Species diversity in space and time: Cambridge University Press; 1995.

32. Storch D, Bohdalkova E, Okie J. The more-individuals hypothesis revisited: the role of community abundance in species richness regulation and the productivity-diversity relationship. Ecol Lett. 2018;21(6):920-37. Epub 2018/04/17. doi: 10.1111/ele.12941.

33. Hurlbert AH, Stegen JC. When should species richness be energy limited, and how would we know? Ecol Lett. 2014;17(4):401-13. Epub 2014/01/08. doi: 10.1111/ele.12240.

34. Etienne RS, Cabral JS, Hagen O, Hartig F, Hurlbert AH, Pellissier L, et al. A minimal model for the latitudinal diversity gradient suggests a dominant role for ecological limits. The American Naturalist. 2019;194(5):E122-E33. Epub 2019/10/16. doi: 10.1086/705243.

35. Pontarp M, Bunnefeld L, Cabral JS, Etienne RS, Fritz SA, Gillespie R, et al. The latitudinal diversity gradient: Novel understanding through mechanistic eco-evolutionary models. Trends in Ecology and Evolution. 2019;34(3):211-23. doi: 10.1016/j.tree.2018.11.009.

36. Descombes P, Leprieur F, Albouy C, Heine C, Pellissier L. Spatial imprints of plate tectonics on extant richness of terrestrial vertebrates. J Biogeogr. 2017;44(5):1185-97. doi: 10.1111/jbi.12959.

37. Jenkins CN, Pimm SL, Joppa LN. Global patterns of terrestrial vertebrate diversity and conservation. Proceedings of the National Academy of Sciences of the United States of America. 2013;110(28):E2602-10. Epub 2013/06/28. doi: 10.1073/pnas.1302251110.

38. IUCN. IUCN Red List of threatened species. 2020 [cited 2020 1 Januray]. Available from: http://www.iucnredlist.org

39. BirdLife International. Data zone BirdLife internatinal 2020 [cited 2020 1 January]. Available from: http://datazone.birdlife.org.

40. Meseguer AS, Antoine PO, Fouquet A, Delsuc F, Condamine FL, McGill B. The role of the Neotropics as a source of world tetrapod biodiversity. Global Ecol Biogeogr. 2020;29(9):1565-78. doi: 10.1111/geb.13141.

41. Bininda-Emonds OR, Cardillo M, Jones KE, MacPhee RD, Beck RM, Grenyer R, et al. The delayed rise of present-day mammals. Nature. 2007;446(7135):507-12. Epub 2007/03/30. doi: 10.1038/nature05634.

42. Kuhn TS, Mooers AØ, Thomas GH. A simple polytomy resolver for dated phylogenies. Methods in Ecology and Evolution. 2011;2(5):427-36. doi: 10.1111/j.2041-210X.2011.00103.x.

43. Jetz W, Thomas GH, Joy JB, Hartmann K, Mooers AO. The global diversity of birds in space and time. Nature. 2012;491(7424):444-8. Epub 2012/11/06. doi: 10.1038/nature11631.

44. Pyron RA, Burbrink FT. Early origin of viviparity and multiple reversions to oviparity in squamate reptiles. Ecol Lett. 2014;17(1):13-21. Epub 2013/08/21. doi: 10.1111/ele.12168.

45. Pyron RA, Wiens JJ. Large-scale phylogenetic analyses reveal the causes of high tropical amphibian diversity. Proceedings of the Royal Society B: Biological Sciences. 2013;280(1770):20131622. Epub 2013/09/13. doi: 10.1098/rspb.2013.1622.

46. Grimm V, Railsback SF. Pattern-oriented modelling: a 'multi-scope' for predictive systems ecology. Philosophical Transactions of the Royal Society B. 2012;367(1586):298-310. Epub 2011/12/07. doi: 10.1098/rstb.2011.0180.

47. Grimm V, Revilla E, Berger U, Jeltsch F, Mooij WM, Railsback SF, et al. Pattern-oriented modeling of agent-based complex systems: Lessons from ecology. Science. 2005;310(5750):987-91. doi: 10.1126/science.1116681.

48. Kucherenko S, Albrecht D, Saltelli A. Exploring multi-dimensional spaces: A comparison of Latin hypercube and quasi Monte Carlo sampling techniques. arXiv preprint arXiv:150502350. 2015.

49. Storch D, Okie JG, Field R. The carrying capacity for species richness. Global Ecol Biogeogr. 2019;28(10):1519-32. doi: 10.1111/geb.12987.

50. R Core Team. R: A Language and Environment for Statistical Computing. In: Computing RFfS, editor. Vienna, Austria2020.

51. Bar-On YM, Phillips R, Milo R. The biomass distribution on Earth. Proceedings of the National Academy of Sciences of the United States of America. 2018;115(25):6506-11. Epub 2018/05/23. doi: 10.1073/pnas.1711842115.

52. Brown JH. On the relationship between abundance and distribution of species. The American Naturalist. 1984;124(2):255-79. doi: 10.1086/284267.

53. Gaston KJ. Species-range-size distributions: patterns, mechanisms and implications. Trends Ecol Evol. 1996;11(5):197-201. doi: 10.1016/0169-5347(96)10027-6.

54. Paradis E, Claude J, Strimmer K. APE: Analyses of Phylogenetics and Evolution in R language. Bioinformatics. 2004;20(2):289-90. Epub 2004/01/22. doi: 10.1093/bioinformatics/btg412.

55. Aldous DJ. Stochastic models and descriptive statistics for phylogenetic trees, from Yule to today. Statistical Science. 2001;16(1):23-34. doi: 10.1214/ss/998929474.

56. Bortolussi N, Durand E, Blum M, Francois O. apTreeshape: statistical analysis of phylogenetic tree shape. Bioinformatics. 2006;22(3):363-4. Epub 2005/12/03. doi: 10.1093/bioinformatics/bti798.

57. Janzen T, Höhna S, Etienne RS, Paradis E. Approximate Bayesian Computation of diversification rates from molecular phylogenies: introducing a new efficient summary statistic, the nLTT. Methods in Ecology and Evolution. 2015;6(5):566-75. doi: 10.1111/2041-210x.12350.

58. Hartig F, Calabrese JM, Reineking B, Wiegand T, Huth A. Statistical inference for stochastic simulation models--theory and application. Ecol Lett. 2011;14(8):816-27. Epub 2011/06/18. doi: 10.1111/j.1461-0248.2011.01640.x.

59. Leprieur F, Descombes P, Gaboriau T, Cowman PF, Parravicini V, Kulbicki M, et al. Plate tectonics drive tropical reef biodiversity dynamics. Nature Communications. 2016;7:11461. doi: 10.1038/ncomms11461.

60. Rahbek C, Graves GR. Multiscale assessment of patterns of avian species richness. Proceedings of the National Academy of Sciences of the United States of America. 2001;98(8):4534-9. doi: 10.1073/pnas.071034898.

61. Hagen O, Hartmann K, Steel M, Stadler T. Age-dependent speciation can explain the shape of empirical phylogenies. Syst Biol. 2015;64(3):432-40. doi: 10.1093/sysbio/syv001.
