# Supplementary material for: gen3sis: A general engine for eco-evolutionary simulations of the processes that shape Earth’s biodiversity
Source: PLoS Biol. 2021 Jul 12;19(7):e3001340. doi: 10.1371/journal.pbio.3001340 (PMC8384074; doi:10.1371/journal.pbio.3001340)
Supplement: S2 Note — (DOCX) [file pbio.3001340.s021.docx]

## Supporting Information Note S2

## Case Study: Does trait evolution impact biodiversity dynamics?

We present here a hypothetical experiment from an extensively studied system – oceanic islands [1]. This case study is accompanied by a step-by-step design of a simulation experiment, including landscape dynamics and input files (see gen3sis R-package vignette). Oceanic islands are associated with well-known empirical patterns and theoretical expectations of how island area [2] and species richness should vary over geological time frames [3, 4]. Oceanic islands emerge from volcanic activity, progressively increasing in area and habitat heterogeneity [2]. Once the island position no longer overlaps with the volcanic hotspot, it erodes slowly to become a low-lying atoll [2, 3]. The resulting hump-shaped trend in island area and habitat heterogeneity is thus expected to generate a hump-shaped species richness pattern over time [5-8], which has been largely reported for empirical systems [8] and has emerged from a couple of mechanistic models [5, 6, 8]. Hence, by designing an experiment inspired by island geological dynamics, the model must be able to generate the expected diversity patterns.

### 1. Experimental design

We used gen3sis to build an experiment from scratch for island dynamics to test whether we can obtain the expected hump-shaped richness over time solely by simulating area dynamics (i.e. no habitat heterogeneity in terms of environmental gradients). We still expected a hump-shaped richness over time, as previously demonstrated by simulation experiments with only area dynamics [5]. Because we opted to keep emergent speciation and extinction, we needed spatial and temporal climatic dynamics to enable speciation by isolation and extinction by conditions becoming unsuitable and by competition. To evaluate how environmental dynamics, trait evolution and ecology interact to ultimately produce a hump-shaped species richness pattern over time, we explored the rate of trait evolution in relation to the temporal environmental variability. We expected that the relative difference between environmental dynamics and the capacity of lineage adaptability to new tolerances would affect extinction and speciation dynamics [9].

### 2. Theoretical Island

We considered a conceptual isolated oceanic island whose number of sites and heterogeneity vary over time (Supporting Information, Animation S3). The island is initiated with four sites (at 140 Ma), but the island surface progressively increases to its maximum of 81 sites (at 90 Ma) and then shrinks back to four sites at the present time. Erosion happens at double the pace of orogeny (Figure S7 A). Each site is characterized by temperature and precipitation values that change at every time-step randomly, following a normal distribution with a mean temperature of 20 °C and mean precipitation of 500 mm/year, with 0.5 °C and 50 mm/year as the respective standard deviations.

### 3. Model

We explored the formation of the hump-shaped species richness curve over time under different rates of trait evolution by simulating three scenarios of trait evolutionary rates (as the standard deviation around the temperature and precipitation optima of the ancestor value), which compare with the temporal variability in temperature and precipitation: (i) low (0.3°C and 30 mm/year), (ii) equal (0.5°C and 50 mm/year), and (iii) high (0.7°C and 70 mm/year). Populations could only disperse to surrounding sites at each time-step. We used a divergence threshold where speciation events occur after 8 myr of isolation. For the local ecological process, species survive as long as their temperature and precipitation optimum values are within ±1.5 °C and ±150 mm of the site temperature and precipitation, respectively. We initiated the simulations with four ancestral species in each initial site.

### 4. Results and Discussion

This simple model and experiment reproduced the expected hump-shaped temporal trends in species richness in all scenarios, even if only slightly in the scenario of low trait evolution. We also observed a delay in the richness peak compared with the maximum island size, which was also previously reported with other island models [5-7], both at the population level [5, 6] and the assemblage level [7]. A stronger decrease in the last erosion phase would be expected if we had continued the decrease in island area. Stopping at 4 grid cells, as in our experiment, the diversity hump is more noticeable at high trait evolution. Our experiment, however, revealed potential interesting novel information, namely that the amplitude and shape of the hump might depend on the relationship of trait evolution compared with environmental dynamics. Here, the richness peak was higher and flatter when the rate of trait evolution was below the environmental temporal variance than when the rate of trait evolution was equal to or higher than the environmental variability. In previous simulation experiments, flattened richness curves have been reported only for stable island area heterogeneity [3].

We further found that the delay in the richness peak was greatest under a lower rate of trait evolution (Figure S7 B). A late moderate decrease is not incongruent with empirical and theoretical patterns on islands, as islands start empty. This makes the species-area-relationships (SARs) during the erosion phase shallower than in the growth phase of the island [5]. Moreover, in our case study, we did not generate habitat heterogeneity within the island. Adding within-island environmental heterogeneity should further increase the diversity hump (i.e. heterogeneity is a known factor driving SARs), as entire environments, and thus also habitat specialists, disappear with erosion. With or without habitat heterogeneity, the islands first accumulate species up to the island’s species carrying capacity [5-7]. In our case study, this species carrying capacity seems dependent on the relationship between trait evolution and temporal variation in the environment. Here, a higher trait evolution rate causes the island to support fewer species because maladaptation occurs. This scenario can be interpreted as the most realistic given global parameters, because only then can we allow populations to evolve maladaptations, which are common in nature.

The lower species richness found under a scenario of high trait evolution can be explained by the species niche trait evolving outside the existing climatic conditions, causing extinction. The resulting variation in the richness peak and the delay of the humped shape suggests that the relative difference between trait evolution rates and underlying temporal environmental variability may contribute the variability in richness trends among different islands and clades [10]. Such patterns could be a starting point for further studies investigating the role of different macro-eco-evolutionary regimes in the emergence of biodiversity patterns on islands.

### 5. Perspectives

Besides trait evolution and climatic variation, we expected that modifying the ecological tolerances would affect the pattern as well, with increasing tolerances compared with climatic variation also flattening the temporal trend. This is because both increased tolerance and low trait evolution cause more species to survive in the model and thus undergo stronger competition, creating an emergent upper boundary for species richness or species carrying capacity. We also expected that adding habitat heterogeneity within the island would increase magnitude of the diversity hump, as entire environments would only be present at larger area sizes. If the added habitat heterogeneity is structured as on oceanic islands (e.g. a central mountain), we might further expect within-island spatial richness gradients, such as elevational richness gradients. In our current experiment, without environmental gradients, we expected to find no clear spatial diversity gradient, except for a slight increase towards the centre of the landscape due to geometrical constraints and edge effects [11]. Further experiments could make niche evolution more realistic by making it either a species-specific parameter or dependent on a particular species-specific parameter, such as temperature [5, 6] – e.g. higher evolution with increasing temperature or decreasing body mass, due to metabolic constraints [12]. Further studies could be conducted to explore mechanisms and parameters in future simulations with the gen3sis engine.

### 6. References

1. Warren BH, Simberloff D, Ricklefs RE, Aguilee R, Condamine FL, Gravel D, et al. Islands as model systems in ecology and evolution: prospects fifty years after MacArthur-Wilson. Ecol Lett. 2015;18(2):200-17. doi: 10.1111/ele.12398.

2. Huppert KL, Perron JT, Royden LH. Hotspot swells and the lifespan of volcanic ocean islands. Science Advances. 2020;6(1):eaaw6906. doi: 10.1126/sciadv.aaw6906.

3. Whittaker RJ, Fernández-Palacios JM. Island biogeography: ecology, evolution, and conservation: Oxford University Press; 2007.

4. Warren BH, Hagen O, Gerber F, Thebaud C, Paradis E, Conti E. Evaluating alternative explanations for an association of extinction risk and evolutionary uniqueness in multiple insular lineages. Evolution. 2018;72(10):2005-24. Epub 2018/08/29. doi: 10.1111/evo.13582.

5. Cabral JS, Wiegand K, Kreft H. Interactions between ecological, evolutionary and environmental processes unveil complex dynamics of insular plant diversity. J Biogeogr. 2019. doi: 10.1111/jbi.13606.

6. Cabral JS, Whittaker RJ, Wiegand K, Kreft H, Emerson B. Assessing predicted isolation effects from the general dynamic model of island biogeography with an eco‐evolutionary model for plants. J Biogeogr. 2019. doi: 10.1111/jbi.13603.

7. Borregaard MK, Matthews TJ, Whittaker RJ, Field R. The general dynamic model: towards a unified theory of island biogeography? Global Ecol Biogeogr. 2016;25(7):805-16. doi: 10.1111/geb.12348.

8. Borregaard MK, Amorim IR, Borges PA, Cabral JS, Fernandez-Palacios JM, Field R, et al. Oceanic island biogeography through the lens of the general dynamic model: assessment and prospect. Biol Rev Camb Philos Soc. 2017;92(2):830-53. Epub 2016/03/01. doi: 10.1111/brv.12256.

9. Aguilee R, Gascuel F, Lambert A, Ferriere R. Clade diversification dynamics and the biotic and abiotic controls of speciation and extinction rates. Nat Commun. 2018;9(1):3013. Epub 2018/08/03. doi: 10.1038/s41467-018-05419-7.

10. Gillespie RG, Baldwin BG. Island biogeography of remote archipelagoes. The theory of island biogeography revisited. 2010:358-87.

11. Colwell RK, Lees DC. The mid-domain effect: geometric constraints on the geography of species richness. Trends Ecol Evol. 2000;15(2):70-6. doi: <https://doi.org/10.1016/S0169-5347(99)01767-X>.

12. Allen AP, Gillooly JF, Savage VM, Brown JH. Kinetic effects of temperature on rates of genetic divergence and speciation. Proceedings of the National Academy of Sciences. 2006;103(24):9130-5. doi: 10.1073/pnas.0603587103.
