# Supplementary material for: gen3sis: A general engine for eco-evolutionary simulations of the processes that shape Earth’s biodiversity
Source: PLoS Biol. 2021 Jul 12;19(7):e3001340. doi: 10.1371/journal.pbio.3001340 (PMC8384074; doi:10.1371/journal.pbio.3001340)
Supplement: S3 Note — (DOCX) [file pbio.3001340.s022.docx]

## Supporting Information Note S3

## Pseudo-code: gen3sis

**Package:** gen3sis [General Engine for Eco-Evolutionary Simulations]

**Function:** run_simulation

**Parameters:**
- config = [file or config object] configuration file path or configuration object in which the initial conditions, biological functions and their parameter values, as well as technical settings for the model can be set.
- landscape = [desired landscape] landscape directory where the all_geo_hab and distance_matrices reside.
- call_observer = [all, steps or NA] tells when the observer function is called.

**Pseudo-code:**

| **!** prepare_directories **!** **!** check_input_data **!** **!** attribute_ancestor **!** **!** init_simulation **!**  **IF** possible to restore_state  **!** restore_state **!**  **FOR** timesteps   \| **IF** max_number_of_species reached  flag = "max_number_species"  **! STOP** max number of species reached, breaking loop **!**  **!** setup_landscape **!**  **!** restrict_species **!**  **!** setup_distance_matrix **!**  **!** loop_speciation **!**  **!** loop_dispersal **!**  **!** loop_evolution **!**  **!** loop_ecology **!**  **IF** max_number_coexisting_species reached  flag = "max_number_coexisting_species"  **! STOP** max number of co-occuring species reached, breaking loop **!**  **IF** present timestep in call_observer  **!** call_observer **!**  **!** update_summary_statistics **!**  **!** save_state for restart **!** \| \| --- \|   **IF** flag OK  **!** print all OK **!**  **!** save phylogeny **!**  **!** make_summary including flag **!** **!** return summary **!** |
| --- | --- |
