# Supplementary material for: gen3sis: A general engine for eco-evolutionary simulations of the processes that shape Earth’s biodiversity
Source: PLoS Biol. 2021 Jul 12;19(7):e3001340. doi: 10.1371/journal.pbio.3001340 (PMC8384074; doi:10.1371/journal.pbio.3001340)
Supplement: S14 Fig — Correlation of model parameters and 3 emerging patterns for all models and landscapes considering deep-time environmental dynamics (A) M0 L1, (B) M0 L2, (C) M1 L1, (D) M1 L2, (E) M2 L1, and (F) M2 L2. Emerging patterns: (i) phylogeny beta is the phylogenetic tree imbalance statistic measured as the value that maximizes the likelihood in the β-splitting model; (ii) range quant 0.95% is the value of the 95% quantile of the species range area distribution; (iii) LDG % loss is the slope of the linear regression of species richness; (iv) richness r is the highest Pearson correlation between simulated and empirical α-diversity; (v) nLTT diff is the lowest difference between simulated and empirical nLTT curves; and (vi) LDG curve r is the highest Pearson correlation between simulated and empirical standardized mean species number per latitude. Data presented available in S3 Data at https://zenodo.org/record/5006413. LDG, latitudinal diversity gradient; nLTT, normalized lineage though time. (PDF) [file pbio.3001340.s018.pdf]

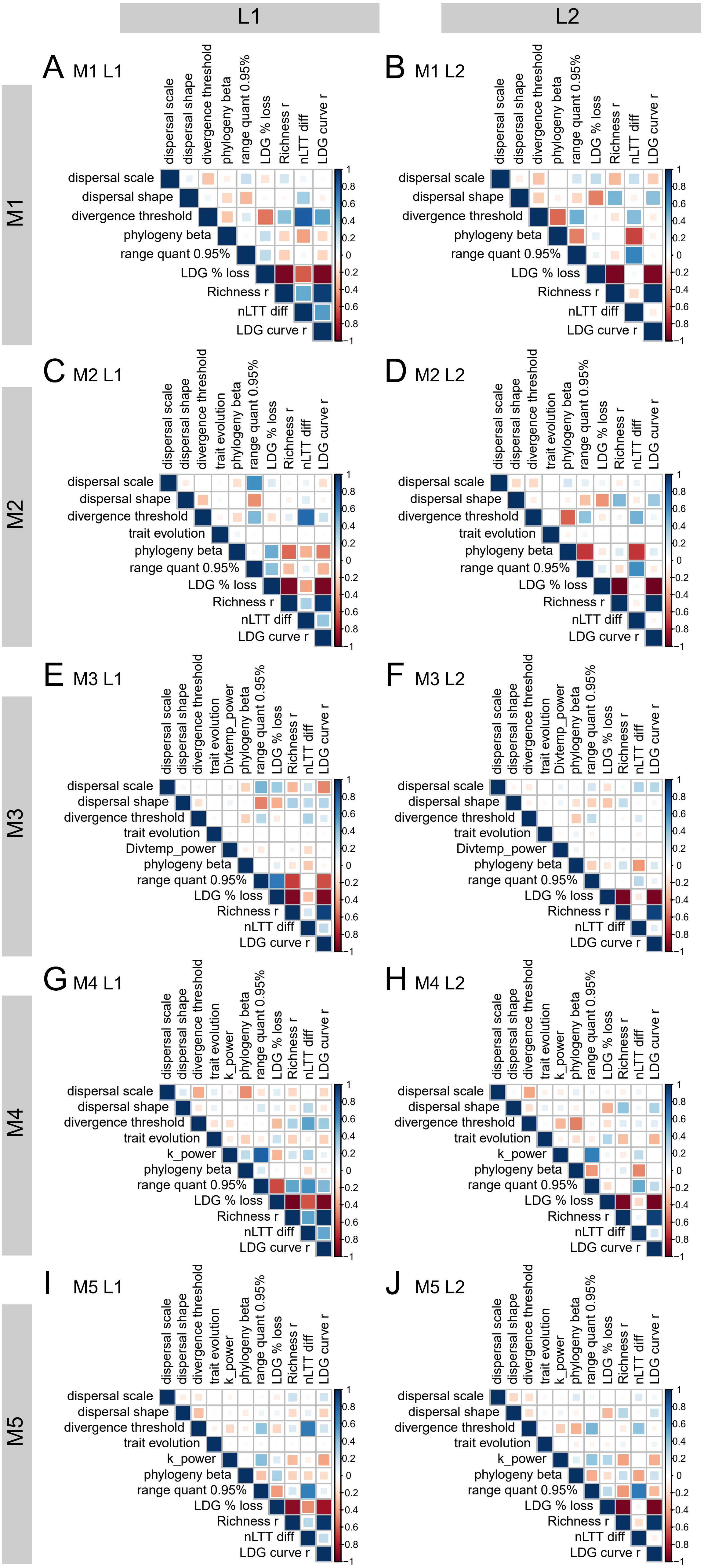
L1
L2
M1
M2
M3
M4
M5
A M1 L1
B M1 L2
C M2 L1
D M2 L2
E M3 L1
F M3 L2
G M4 L1
H M4 L2
I M5 L1
J M5 L2
dispersal scale
dispersal shape
divergence threshold
trait evolution
Divtemp_power
k_power
phylogeny beta
range quant 0.95%
LDG % loss
Richness r
nLTT diff
LDG curve r
1
0.8
0.6
0.4
0.2
0
-0.2
-0.4
-0.6
-0.8
-1
