# Supplementary material for: gen3sis: A general engine for eco-evolutionary simulations of the processes that shape Earth’s biodiversity
Source: PLoS Biol. 2021 Jul 12;19(7):e3001340. doi: 10.1371/journal.pbio.3001340 (PMC8384074; doi:10.1371/journal.pbio.3001340)
Supplement: Attachment — Submitted filename: Response_Reviewers_gen3sis.docx [file pbio.3001340.s023.docx]

*****************************************************
EDITOR COMMENTS:

Thank you very much for submitting your manuscript "gen3sis: The GENeral Engine for Eco-Evolutionary SImulationS on the origins of biodiversity" for consideration as a Methods and Resources paper at PLOS Biology. Your manuscript has been evaluated by the PLOS Biology editors, an Academic Editor with relevant expertise, and by three independent reviewers.
You'll see that while the reviewers recognise the potential merits of your approach, they are all struggling to identify the clear advance or unique utility that gen3sis represents over previous initiatives. While the open availability of your platform is a bonus, the reviewers are currently not fully persuaded by the examples presented or by your claims to be able to meet the stipulated challenges.
The reviews of your manuscript are appended below. Based on their specific comments and following discussion with the academic editor, I regret that we cannot accept the current version of the manuscript for publication. We remain interested in your study and we would be willing to consider resubmission of a comprehensively revised version that thoroughly addresses all the reviewers' comments. Note that one reviewer says "if space is an issue"; at PLOS Biology we have no space restrictions, but the manuscript should remain well structured and accessible. We cannot make any decision about publication until we have seen the revised manuscript and your response to the reviewers' comments. Your revised manuscript would be sent for further evaluation by the reviewers.
We appreciate that these requests represent a great deal of extra work, and we are willing to relax our standard revision time to allow you six months to revise your manuscript. We expect to receive your revised manuscript within 6 months.

**Response:** Thank you for this in-depth review. We appreciate all the reviewer comments and recognize how they helped to strengthen this manuscript. We see that the common struggle in recognizing the utility and uniqueness of our engine is partially due to the novelty of this approach and is definitely a sign that we needed to clarify important points in the manuscript.

One source of confusion seems to be associated with the way we presented our case studies. Our initial idea was to focus on presenting gen3sis as the novel and general “tool” it is. The case studies were initially meant as examples that would show, concisely and intuitively, the flexibility and novelty of gen3sis. Our ambition was not to use one unique case study to answer specific scientific questions. We do, however, see how the lack of detail in our case studies contributed to some of the criticism raised by the reviewers. We see that exploring the case studies in further depth would be advantageous to better showcase the utility and flexibility of gen3sis and to tackle the points raised by the reviewers. To increase the clarity of our manuscript in general and in our case studies in particular, we have strengthened the global case study substantially and, as suggested by reviewer 1, have moved the island case study to the Supporting Information.

Furthermore, we have clarified the entire manuscript by making clear statements about the novelty of gen3sis. We now relate it more clearly to the literature and model simulations used in previous studies. Specifically, we have added a more thorough revision of population-level eco-evolutionary models and have included more examples and details in the main text. For the global case study, we have compiled an entirely new paleo-reconstruction, implemented two new models and tested multiple biodiversity patterns by adding empirical phylogenetic data and species range distributions. We feel that this substantial addition has led to a more intuitive and complete presentation of the case studies, which in turn more convincingly support the value and novelty of our approach.

*****************************************************
REVIEWERS' COMMENTS:

## Reviewer #1:

Hagen and colleagues present a tool to simulate eco-evolutionary processes. The simulation framework can be used to generate null models of biodiversity, and to test macroecological and macroevolutionary processes across spatio-temporal scales. The framework is flexible, allowing users to specify various parameters. The authors suggest the platform represents a much-needed step forward in understanding the processes that shape biodiversity over Earth history. Similar simulations have been used to test potential processes that influence biodiversity, but these frameworks are not readily available for the broader community. Thus, this tool will be of broad interest but is not novel, and there are now numerous studies that have employed similar frameworks (see below).

**Response:** Thank you for your thorough review and constructive input. We claim that the novelty of our approach, compared to previous ones, lies in its flexibility, generality and accessibility. We tried to convey this novelty in the revised version or our manuscript and to discuss previous models in greater detail.

Specifically, in the revision we took particular care to explain the customizable features of the model and how the model can accommodate various mechanisms that have been proposed to be associated with the formation of biodiversity patterns. With this said, we also recognize that similar approaches have been used previously. In the revision, we better explain that previous models have improved our knowledge immensely and have been successful in answering specific questions, but they are less flexible and less suited to hypothesis testing and replicability of virtual experiments compared with the approach proposed here.

To emphasize the novelty of gen3sis and to distinguish it from previous contributions, we have made major changes in the manuscript and rephrased the text so that the specific novelty of this model is clearer (e.g. lines 90-99, 116-119, 128-136, 152-154, 180-183, 198-200, 228-230, 613-627), particularly in relation to other studies (lines 78-98, 497-507, 586-590). We have made related changes in Notes S1 and S2. We also added a literature review of the eco-evolutionary and spatio-temporal mechanistic models operating at the population level (lines 97-98). In addition, we improved our global case study, focusing on the latitudinal diversity gradient (LDG), by presenting three different model configurations corresponding to three mechanisms (New Figure 3 and Table S1).

In general, I think this is a worthwhile contribution. However, there are a number of issues that need to be addressed prior to publication. Chief among them is a lack of clarity regarding the parameters and functionality of the simulation framework. More detail needs to be provided on virtually all aspects of the simulation framework, outlined below.

**Response:** Thank you. We made substantial modifications in the manuscript text (lines 101-103, 116-119, 128-156, 181-183, 198-200, 624-627 and the ‘core functions and objects’ section in general). Now the main manuscript, Notes S1 and S2, and the R-package vignette and documentation address these points more clearly. We also added a new figure (Figure 3) and a supplementary table (Table S1) to make the processes and parameters easier to understand.

The authors also provide two examples of implementation of the simulation framework. These examples seem half-baked, without clear hypotheses, null models, or explanation of how the simulations were performed. The authors need to spend more time discussing both examples, or, if space is an issue, eliminate one example so that the other can be given due diligence. Both of these are essential changes.

**Response:** We fully agree. The initial goal was to provide two short examples to showcase the engine use while still maintaining a clear focus on the main methods. However, we acknowledge that this led to rather limited and superficial case studies, which poorly reflected the potential of the model. The simple case study (#1) led to misinterpretations and did not clearly demonstrate the advantages of the presented method. To strike a better balance between engine presentation and in depth case studies, we decided to move former Case Study 1 to the Supporting Information and expanded the former Case Study 2 considerably, as suggested. We developed three models with three different mechanisms to explain the LDG through different pathways. Furthermore, we compiled an entirely new paleo-reconstruction in order to better account for uncertainties in the paleo-environmental conditions as input to the simulation model. This extended case study still focuses on illustrating the novelty and flexibility of gen3sis, but now also improves the fundamental understanding of the formation of the LDG. Specifically, we found that a carrying capacity linked with energy was the only model variant that could simultaneously produce a realistic LDG, species range size frequencies, and phylogenetic tree balance (lines 43-45).

1.    One of the benefits of a simulation framework is the ability to test competing processes and hypotheses, and to generate null models. The authors do not adequately formulate hypotheses for how the processes in the model affect biodiversity patterns, nor do they implement null models. For example, in the abstract they indicate that: "Via the process of niche evolution in response to changes in climate and the spatial distribution of habitat, our model predicts realistic island biodiversity dynamics, as well as a global latitudinal biodiversity gradient and species richness distribution". However, it is not at all clear what this means, and how niche evolution does or does not affect biodiversity patterns. Is niche evolution necessary to generate realistic biodiversity patterns? How does niche evolution produce the patterns, and which processes in the simulation (e.g., species, dispersal, extinction, carrying capacity) contribute most to the patterns they find? At what rate do niches need to evolve? These questions remain unclarified in the body of the text.

**Response:** We agree, and thus made an in-depth investigation of the mechanisms shaping the LDG. We referred to the literature to generate three different configurations for the simulation models. We now compare the outputs of the three different configurations and thus mechanisms (lines 119-126, 347-366). For example, a long-standing question in ecology and evolutionary biology is the relative importance of mechanisms of diversification rate and carrying capacity in the formation of the LDG (new Figure 3). We now investigate these mechanisms and find that the mechanisms of carrying capacity provide the best support as regards to the empirical patterns. In addition, we now consider a null-model (M0), as suggested. We improved the manuscript in terms of describing both a biological phenomenon and the utility of gen3sis. Thank you.

2.    The case studies were useful for assessing how the simulations can be applied. However, similar to my comment above, it was not at all clear what the reader should take away from these exercises. In other words, I was left wondering what the authors learned from the modelling efforts: which processes were important in generating the realistic biodiversity patterns? Was it the process of speciation? Extinction? Dispersal?

**Response:** We agree that the previous case studies were too simple and did not convey the usefulness and novelty of our approach, and we have restructured them considerably (see above). We recognize that more could be done in the way we describe how gen3sis can tell us more about specific processes. We indicate the superiority of a carrying capacity mechanisms over speciation rate differences (lines 41-45, 471-476) and specially the correlation and significance of dispersal and ecological processes (new Table S2 and S3).

Again, the benefit of a simulation framework is the ability to test, explicitly, the relative contribution of processes to the generation of biodiversity, and to examine what type of patterns result if those processes are not employed (i.e., a null model). If space is a limitation, the authors should focus on only one case study and better explain both the methods and results to readers. In short, I was disappointed in the examples and poor explanation provided by the authors, especially since the authors suggest that these types of simulations can provide 'general rules' for the generation of biodiversity, and yet they do not focus on what they found regarding 'rules' of biodiversity, nor do they employ null models to ensure results are unique to specific processes.

**Response:** We agree with your point and thus focused on only one case study in the main manuscript. We now frame baseline mechanisms in a null-model (M0), which we compare with mechanisms of diversification rate (M1) and carrying capacity (M2) (lines 390-429). We have now conducted model comparisons and a sensitivity analysis of the parameters (lines 431-518, Note S1), and we discuss the mechanism behind the LDG without losing the focus of presenting the engine. A more in-depth test of more LDG models is planned as a follow-up study devoted to the LDG and not on presenting the model.

3.    Line 79: One of the unique aspects provided by the authors' simulation framework is the ability to assess the effect size of certain variables on the generation of realistic biodiversity patterns. This can be done by gradually increasing model complexity, and by implementation of null models. The authors should discuss this benefit in more detail (around line 79) and employ this framework in their example case studies.

**Response:** We agree and have implemented this suggestion (lines 79-88). We now propose a case study (lines 119-126) where we consider three different configurations, starting with a simple implementation corresponding to a null-model (M0) and then adding complexity in the form of a modification of the diversification rate (M1) and carrying capacity (M2).

4.    The authors note that 'practical implementations of simulations have yet to be consolidated or widely used' (line 74). This is not strictly accurate, and the authors should point readers to notable exceptions, such as those below (not an exhaustive list):
Rangel, T. F. et al (2018). Modeling the ecology and evolution of biodiversity: Biogeographical cradles, museums, and graves. Science, 361(6399).
Rangel, T. F. et al (2007). Species richness and evolutionary niche dynamics: a spatial pattern-oriented simulation experiment. The American Naturalist, 170(4), 602-616.
Saupe, E. E. et al (2019). Spatio-temporal climate change contributes to latitudinal diversity gradients. Nature ecology & evolution, 3(10), 1419-1429.
Saupe, E. E. et al (2020). Extinction intensity during Ordovician and Cenozoic glaciations explained by cooling and palaeogeography. Nature Geoscience, 13(1), 65-70.
Saupe, E. E. et al (2019). Non‐random latitudinal gradients in range size and niche breadth predicted by spatial patterns of climate. Global Ecology and Biogeography, 28(7), 928-942.

**Response:** We agree. Thank you! Nonetheless, including our own work (Leprieur et al. 2016, Descombes et al. 2018, Gaboriaud et al. 2019) there are still only a handful of studies on the topic, compared to the hundreds of papers on mechanistic simulation models used in other fields (e.g. climate modelling). Hence, we remain convinced that, in general, simulation models could be more broadly used in macroecology and macroevolution, and we present a user-friendly and flexible approach that could enable broader investigations. We thank you for pointing out these articles, and we made sure to cite all of them in the revised manuscript. This allowed us to better relate our work to previous literature and, at the same time, to better explain the novelty of gen3sis (lines 76-101, 497-503, 586-596).

5.    Line 82 and 90: Gotelli and colleagues and Saupe and colleagues have noted that simulations can be used to examine how continental configuration and paleogeography influence biodiversity dynamics, which also provide examples of simulations on million-year time frames (e.g., Saupe et al. 2020. Nature Geoscience).

**Response:** We added these references in the revised manuscripts. Thank you for pointing out this omission.

6.    The authors should provide clarity on the process of extinction in the simulation framework. When does extinction occur, and can extinction criteria be modified by the user?

**Response:** This is a very good point. We agree that how the process of extinction happens was not clearly explained in the previous version of the manuscript. Extinction of populations within sites occur as a consequence of the ecology function in the ecological module of the proposed approach. Moreover, if all the sites that a species occupies become unsuitable, the species becomes extinct over its entire range. Hence, range-wide extinction (and speciation for any evolutionary matter) is an emergent process. We acknowledge that this is a very important point and thus made extinction processes more evident and clearer in the text (lines 128-136), specifically in the ‘core function’ section (lines 210-214, 245-248, 306-307) and in the legend of Figure 2.

7.    The authors refer to 'barriers' within the manuscript, but it was unclear whether such barriers can be specified, and how they could be specified. I.e., could a separate input file representing rivers, mountain ranges, etc be used to limit dispersal? Or, is climate the only potential barrier? Similarly, the authors indicate that oceanic pixels/cells are inhabitable for simulations focused on the terrestrial realm: this is very surprising, and I would like the authors to justify this choice (see line 363). Is this the case for the simulations in general, or can this feature be 'turned off'?

**Response:** Thank you. We agree that the concepts of barrier and inhabitable site were not clear. Climate or any attribute of the landscape can be used as a barrier (e.g. rivers and mountains), but they need to be specified when defining the connectivity among sites as part of preparing the input files before running the simulation. For instance, in the case study, we considered higher resistance of aquatic cells than terrestrial cells to dispersal when applying the costdistance function (lines 147-148, 178-183, 394-396). Similarly, the cells that are considered unsuitable for the clade are defined when preparing the input landscape. In our case study, since we model terrestrial organisms, all aquatic sites were set to NA. In contrast, the cells that are suitable or unsuitable for species within this clade (hence shifting within one simulation as a result of evolution) are defined by the ecological constraints set in the ecology function (lines 306-307).

8.    The simulation framework allows for trait divergence and trait evolution. This is excellent, but the authors should specify what types of traits can be modelled explicitly in the manuscript.

**Response:** Thank you for emphasizing this. Trait divergence and evolution of multiple traits are possible with gen3sis, the traits just have to be specified for the configuration object and the user has to specify how they function within the core functions. We changed several sections of the manuscript, i.e. configuration, dispersal, evolution and ecology (e.g. lines 198-200, 292-303), to make this aspect clearer.

9.    The links provided in the manuscript to the code are broken.

**Response:** Thank you for spotting this issue. We fixed the SI link and added the link to a development repository so that suggestions and bugs can be reported by the community.

10.    The authors generally describe the functions in the simulation framework, but each could benefit from more detail on options and functionality.

**Response:** We reformulated all core functions and added modification examples to help clarify how they can be used. In particular, for each core function, we now present what is general about the function of ecology, evolution, speciation and dispersal, followed by how those functions can be modified by the users to account for various mechanisms (lines 207-316). With these changes, options are now clearer to the user.

11.    The process of speciation in the simulation framework was not clear. The authors indicate that clusters/populations gradually accumulate incompatibility, but it was not clear at what rate this occurs (line 223). Can the rate be altered? Is the rate affected by the rate of climate change? The description regarding trait divergence that eventually leads to speciation differs from the description of divergence on line 360, which instead refers to numbers of years for speciation to occur (e.g., 2, 4, 6, 8, 10 Ma). Can the authors clarify?

**Response:** Thank you for pointing this out. Population clusters can accumulate constant differentiation over time, and this rate can also change as a function of the landscape or trait properties. We clarified this throughout the manuscript (lines 128-136, 210-214, 285-289). In addition, in the case study, we added a model (M1) where the differentiation rate among clusters changes with the temperature of the cells (lines 405-413, Figure 3, Note S1 and Table S1). The changes are added by time-step, and the conversion to myr accounts for divergences in time-steps and the speciation threshold. For example, a speciation threshold of 6 under a constant increase of +1 in a ~170 kyr landscape resolution represents one speciation event every 1 myr (i.e. 6 × ~170 kyr) in populations that have been always isolated. In our examples, trait divergence does not influence speciation events, as we only model allopatric speciation. In summary, we made the speciation processes clearer in the manuscript and Supporting Information, changes that are supported by the updated case study.

12.    Conceptual island case study example (line 299): as noted before, this example could benefit from additional explanation. It was difficult to understand the authors' hypotheses/expectations, and their methodology. Did the authors perform simulations to see what processes should be tweaked to generate a flat biodiversity gradient on the island? What amount of trait evolution, and which ecological factors, interact to produce richness gradients?

**Response:** We decided to move this case study to the Supporting Information and focus on one case study, as suggested, and on presenting the methods. This major change helps to increase clarity while keeping the paper’s focus on the main methods. Regarding the conceptual island case study, our intention was to provide a simple hypothetical system for a step-by-step creation of a simulation experiment (explained in detail in the vignette of the R-package). For this, we designed a simple experiment with clear theoretical and empirical expectations based on new frameworks of island biogeography. The case study is now better described, explained and discussed, including clear expectations and explanations of how biodiversity gradients over time differ among scenarios and how other processes and factors can impact richness patterns (Note S2 and Figure S7).

Regarding hypotheses/expectations and methodology, we now explain in more detail the objective of this case study (by presenting a hypothetical experiment step by step – see vignette) and the hypotheses and expectations of this particular experiment: that the model should generate a well-known empirical and theoretical relationship, namely the hump-shaped diversity richness over the island’s geomorphological lifespan. Our hypothesis means that species richness should follow area dynamics with a delay, as observed in other assemblage (Borregaard et al. 2016) and population-level (Cabral et al. 2019a,b) models, even in the absence of habitat heterogeneity (Cabral et al. 2019b). We explored this humped shape further over the relationship between trait evolution and temporal environmental variation, as the model uses variation in the environment to cause local extinctions, range dynamics and thus speciation opportunities via isolation without any further a priori hypothesis.

Regarding analysis questions, we did not look at biodiversity gradients within the island. Because there is no environmental gradient, there is no diversity gradient, perhaps with the exception of a slight increase towards the centre of the landscape due to geometrical constraints and edge effects. The role of trait evolution, environmental gradients and any other factor in generating spatial richness gradients within the island is an interesting question that could be addressed in a separate manuscript. For temporal gradients accurately simulating the area dynamics is key are key to achieving a flattened temporal richness gradient, as shown elsewhere (Borregaard et al. 2016; Cabral et al. 2019). Here, the experiment shows that the relative rate of trait evolution compared to climatic dynamics seems to play a role as well, with a flattening of the temporal richness gradient arising from a trait evolution rate that is lower than the climatic standard deviation. Further tweaks (besides trait evolution and climatic variation) could be also attempted in devoted simulations. Nevertheless, we expect that modifying the ecological tolerances would affect the pattern as well, with increasing tolerances in relation to climatic variation also leading to a flattening of the temporal trend. This is because both increased tolerance and low trait evolution cause more species to survive and thus undergo stronger competition, creating an upper boundary or emergent species carrying capacity. Note also that we do not have habitat heterogeneity (environmental gradients) within the island. Adding this would probably further increase the diversity hump, as entire environments disappear with erosion.

~12.1: the authors note (line 305) that dispersal was limited to surrounding sites. Does this mean that populations could only disperse to surrounding cells? If so, does it follow that speciation could only occur if local extinction happened, which then isolated populations?

**Response:** In this case study, species dispersed just to the surrounding cells in every time-step. The wording was indeed not ideal, and we thank you for pointing this out. We have revised the wording and instead write: “Populations could only disperse to surrounding sites in each time-step. We used a divergence threshold where speciation events occur after 8 myr of isolation” (Note S2 page 2). The entire island case study is now found on the supplementary Note S2 and Figure S7.

~12.1.1: Did the authors consider other dispersal thresholds?

**Response:** We explored multiple dispersal thresholds. We opted to use a Weibull distribution because it generalizes also for an exponential distribution when shape=1. We have now added an entire section on model parameter exploration to the main text and the Supporting Information, as well as a new Figure S2 showing some combinations of the explored and viable dispersal function parameters, such as Weibull shape and scale. We also added to the main text: “… we explored dispersal distributions and parameters ranging in realized mean and 95% quantiles between less than a single cell, i.e. ~50 km for a landscape in 4°, and more than the Earth’s diameter, i.e. ~12’742 km. ” (lines 433-435).

~12.2: What does 'high' and 'low' trait evolution mean?

**Response:** We defined variance in precipitation and temperature as low (i.e. 0.3°C and 30 mm/year) and high (0.7°C and 70 mm/year) in relation to the simulated climatic temporal and spatial variance in the simulation (0.5°C and 50 mm/year). We have moved this information to Note S2.

~12.3: I was surprised to see that diversity did not decrease when the hypothetical island decreased in size by erosional processes, following the species-area relationship. Do the authors have an explanation for why diversity did not decrease, which seems incongruent with empirical biodiversity patterns?

**Response:** Diversity does decrease with area (more noticeable at high trait evolution), but the response shows a lag because extinctions are stochastic and dependent on the temporal dynamics of the environment, as observed in other modelling studies. This is not incongruent with empirical and theoretical patterns on islands, as islands are originally empty. This makes the SARs during the erosion phase shallower than in the growth phase of the island, as found in previous modelling studies (Cabral et al. 2019b). Hence, an island first need to accumulate species up to its species carrying capacity (Borregaard et al. 2016; Cabral et al. 2019a,b), which in our case study depended on the relationship between trait evolution and temporal variation in the environment. Here, higher trait evolution causes the island to support fewer species in general, simply because maladaptations are possible. Note also that we did not consider habitat heterogeneity (environmental gradients) within the island. Adding within-island environmental heterogeneity can be expected to further increase the diversity hump (heterogeneity is a known factor driving SARs), as entire environments, and thus also habitat specialists, disappear with erosion. We have improved the presentation of this case study but also moved it to Note S2, given the increased emphasis on the other case study.

13.    Latitudinal biodiversity gradient case study example: Again, it was not at all clear what were the hypotheses for this case study. Which processes did the authors expect to generate latitudinal biodiversity gradients? Speciation? Extinction? Carrying capacity alone?

**Response:** We now entirely reformulated the global case study about the LDG. We provide a short introduction to the research question and better introduce the main hypotheses that have been proposed in the literature to explain the formation of the LDG. We present (lines 121-126, 347-361, Figure 3) and implement (lines 390-429, Note S1 and Table S1) three alternative hypotheses. These hypotheses are: *time for species accumulation*, variation in *diversification rates*, and variation in *ecological limits*, as categorized by Pontarp et al. (2019) regarding varying speciation and ecological processes (lines 414-429, Figure 3, Note S1, Table S1). We found that model M2 was the best match for all the empirical patterns individually, and the only model able to pass all acceptance criteria (new Table 3). We also found a strong correlation (Table S2) and significance (Table S3) of dispersal shape and carrying-capacity when reproducing the LDG. We discuss this in the revised main manuscript (lines 493-497) and Note S1.

~13.1: Saupe et al in NEE used simulations to evaluate processes that could generate latitudinal biodiversity gradients. From what I can see, the simulations are similar to those performed by Saupe and colleagues, although Saupe et al invoked fewer biotic processes (and therefore their simulations are potentially less realistic). This contribution seems relevant to cite here (line 419), in addition to reference 19, and should also be discussed more thoroughly throughout this case study. How do the findings and patterns of the authors compare to previously published simulations that used a similar simulation framework? Are the processes invoked similar? The same comment could also be made for the study by Rangel et al 2019 (Science), although this study was not global in scale: how do the findings compare to the authors' simulations, which also focused on simulating biodiversity gradients?

**Response:** Saupe et al. (2019) was already cited in another section of the manuscript, but we agree that this study is relevant in the context of the case study and have added a citation there as well. Although comparisons of mechanisms are difficult given the different spatial and temporal resolution of these studies, we were able to discuss some similarities and divergences that should be investigated further in the exploration of the parameters. A major advantage of our approach over those proposed in previous studies (e.g. Rangel et al. 2019, Saupe et al. 2019) is that the time scale of the diversification simulated in the model is realistic. Hence, the simulation model produces phylogenies of realistic scale that span the Cenozoic and can be compared with those of tetrapods. Beyond a simple comparison of species richness patterns, we propose a comparison of simulation outputs with multiple empirical patterns, which provides a greater confidence in their realism while we trade temporal resolution for temporal coverage. We now discuss this in the main text (lines 497-500 and 591-608) and on Note S1.

~13.2: The authors note they initiated the simulations from a single ancestor species over the terrestrial surface (line 347). The wording here is unclear, as is the authors' methodology.  Does this imply that the simulation started from only one grid cell on Earth (if so, which?), or that random grid cells were occupied globally (if so, which?), but that they all belonged to the same species?

**Response:** Thank you for spotting this. The simulations started with one single ancestor (one species) spread across the entire land surface of the Earth at 65 Ma. We made this clear in the revised version (lines 392-396).

~13.3: The authors note on line 382 that 'LDG emerged systematically from the underlying modelled mechanisms', but how? Why? From what mechanisms?

**Response:** We now include a more detailed exploration of the mechanisms underlying the formation of the LDG in a largely improved case study. We expect that this should alleviate confusion regarding this point. “We found that a carrying capacity linked with energy was the only model variant that could simultaneously produce a realistic LDG, species range size frequencies, and phylogenetic tree balance.” lines(42-45). Changes in dispersal and carrying capacity were strongly correlated with the formation of the LDG (lines 491-497 and Note S1).

~13.4: It wasn't clear how speciation or extinction was parameterized in these simulations. Please clarify.

**Response:** Thank you for pointing this out! We agree that speciation and extinction processes were not clearly presented in the methods of the previous manuscript version. Speciation and extinction are processes that emerge from interactions across core processes. For example, while speciation is clearly influenced by the speciation threshold and an increase in differentiation between clusters, speciation is also modulated by the ecology functions that define which part of the landscape is suitable for the species (and thus the level of fragmentation) and by the dispersal functions that ultimately connect or disconnect clusters of populations. Species extinction is directly dependent on the ecology function: when there are no suitable sites in the landscape for a species as defined by the ecology function, then the species will go extinct. In addition, this is a dynamic process and the rate of extinction will interact with the evolution function (the ability to track shifting niches) and the dispersal function (the ability to find new suitable sites across the landscape). We tried to clarify these points throughout the manuscript (lines 128-133, 210-214, 268-272, 306-307 and Figure 2 legend).

~13.5: The authors assume a carrying capacity. This is interesting and likely realistic, but won't this automatically generate more diversity at low latitudes based on the authors' assumption that carrying capacity is predicated on energy? This does not seem a true test of process, as the scientific community already knows that energy is higher at low latitudes. Thus, if you assume more energy allows for a higher carrying capacity, it will automatically allow a latitudinal biodiversity gradient to form. What would happen if the authors do not assume a carrying capacity: is a latitudinal biodiversity gradient produced from the simulations? And, what differed in the simulations that did not produce biodiversity gradients, which the authors reference occurred around 10 percent of the time? I'd like to see more interrogation of these opposing patterns.

**Response:** We agree that in the previous version of the manuscript we only considered a rather complex set of mechanisms. In order to better illustrate how the simulation model enables exploration of various mechanisms, we now implement three hypotheses: a simpler configuration where patterns should be mainly driven by landscape and niche conservatism, a second configuration implementing an effect of temperature on diversification rate, and a third configuration implementing the carrying capacity as in the previous version of the manuscript. All models reproduce LDG patterns; however, the energetic carrying capacity improves overall model prediction when two additional empirical patterns are considered, range size frequencies and phylogenetic tree imbalance. We discuss in more detail the simulations that did or did not result in biodiversity gradients, finding connections with the results of Saupe et al. (2019) and others.

~13.6: The authors note that an increase in K correlated positively with LDG slope, which is not at all surprising. My question is: what other processes were involved? Speciation, extinction, dispersal? What process was dominant - i.e., has the largest effect size?

**Response:** We now include a more complete case study and are able to better relate a variety of mechanisms to the empirical patterns (lines 471-507, Note S1 and new Table S2 and S3). In addition, we performed a more detailed sensitivity analysis. Although further research should be dedicated to the LDG, regarding effect sizes of speciation, extinction and dispersal, we provide more results related to the effects of parameters on patterns. The main objective of our case study is to illustrate the utility of gen3sis and to point out interesting avenues for future investigation.

~13.7: Apologies if I missed this, but I did not see where the authors obtained their empirical richness patterns, from which to compare to their simulations (line 385).

**Response:** This information was indeed missing from the main text. Thanks for noticing this. We added this information to the main text (lines 456-459), providing more details in Note S1. We now also added empirical phylogenies to the study, accounting for 18’978 species and thus two additional large-scale patterns besides LDG.

~13.8: The authors do not indicate how patterns change when parameters are tweaked. Are patterns similar at different thresholds (i.e., they altered scale of dispersal, rates of evolution, speciation time, etc)? What is the sensitivity of biodiversity patterns to model parameterization?

**Response:** Thank you for pointing this out. We now indicate the range of parameters explored during the model development phase and factorial exploration of parameter ranges. Moreover, we present correlations across models and landscapes for processes and patterns, which we discuss in association with previous studies such as Saupe et al. (2019).

~13.9: The climatic data used to power the latitudinal biodiversity gradient example are highly suspect (detailed in note 2S). There are many weaknesses to what the authors have done, although they have clearly put a lot of effort into the reconstructions. The key thing here is that the Koppen reconstructions are qualitative at best, with large uncertainties associated with them. A few (of many) criticisms would be:

*    They are built from the distribution of sediments, but these have large climatic ranges associated with them. For instance, bauxites show that it is seasonally wet (but how seasonal is poorly defined) and warm (above ~22C) (see Price, G., P. Valdes and B. Sellwood, Prediction of modern bauxite occurrence: Implications for climate reconstruction. Palaeogeography Palaeoclimatology Palaeoecology, 1997. 131(1-2): p. 1-13.). The temperature could be anywhere between 22C or 32C, or indeed more! This impreciseness of the indicator is true for pretty much all lithologies used. Hence, the Koppen maps are a broad-brush estimate of the climate (and this is ignoring the fact that, for some time periods, there are large data gaps so the maps will be spatially crude as well).
*    Koppen uses very broad categories. For instance, temperate climates are defined as anything between 0 and 18C! This is not very precise.

**Response:** We agree that the input landscape of the case study has uncertainty. Rather than absolute temperature, the shape and position of the temperature gradient is what matters most for creating realistic biodiversity patterns. Hence, the broad position of the Koppen bands should already capture large-scale temperature gradients along latitude. Therefore, we expect that our reconstructions should capture in essence most of those gradients for investigations of the formation of the LDG. Nevertheless, in order to better account for uncertainties associated with the landscapes, especially related to the paleoelevation and temperature reconstructions, we compiled alternative landscapes using different topographic (Saumpe 2020 and Scotese 2018) and temperature estimation from marine benthic isotopes (Westerhold 2020). We now show that the conclusion about the mechanisms (M0, M1, M2) from the simulations was the same with both of the landscape sets used (L1 and L2). Moreover, we discuss these limitations in much more detail in the revised discussion. While our case study in mainly illustrative, we argue that future studies should use improved paleo-environmental reconstructions and that this should stimulate interdisciplinary collaboration between geologists, climatologists and biologists.

As far as I understand, the authors perform a strange smoothing process (this needs to be clarified) to change these categories into something quantitative, but you could pretty much do anything here.

**Response:** We used a smoothing process to smooth the transition between Köppen zones and reduce the artefact of the border by approximating more realistic temperature transitions. We now provide more details on the input landscape in the main text and Note S1.

They don't seem to think about basic climate phenomena (such as continentality), so the resulting temperatures will not be physically realistic. Other Koppen categories are mainly moisture-based, but the same comments apply.

*    There is a recent paper that indicates temperature using present day lapse rates is extremely inaccurate.
*    Interpolating to 170 kyr is spurious. The data is at stage level (~5Ma) and anything finer has no scientific input.
*    Overall, I would be unconvinced that the reconstructions are quantitatively reliable. At best, they may reflect some very large scale (continental size) gradients, but I would have no confidence on smaller scales.

**Response:** We agree that the landscape dataset presented in the previous manuscript version had limitations and uncertainty, but the main goal of our case study is to illustrate the potential of the approach rather that to present definitive research results about the LDG.

In the revised version, we therefore clearly discuss the limitations of the present landscape and that this approach hinges on accurate paleo-environmental reconstructions. We further explain that we hope that our approach will stimulate more interdisciplinary collaborations between geology, climatology and biology (lines 604-608). In addition, to better account for uncertainty in the use of paleo-environmental reconstructions, we generated a new landscape set, where temperature and elevation data are provided at higher temporal resolution (1 myr or lower: Straume 2020, Westerhold 2020) and consider a different approach to reconstructing paleo-temperature reconstructions from isotope records. We ran the simulations on this second landscape and found that the support for the mechanisms of carrying capacity was conserved. Moreover, we now discuss differences in the light of using gen3sis on multiple reconstructions in Note S1.

We would like to point out that the advantage of running simulations that span the Cenozoic is that the outputs have an appropriate time scale. In particular, the output phylogenies are realistic in scale and their properties can be compared with empirical ones, which was not achieved in previous studies (Rangel et al. 2018, Saupe et al. 2019). Hence, while the paleo-reconstructions used in the case study might suffer from more uncertainty, previous simulations studies had a much shorter time scale, which prevented comparisons of simulation outputs, especially phylogenies, with empirical data.

We agree that we are still, unfortunately, far from having reliable paleo-reconstructions for the Cenozoic. Even if a new paleo-elevation reconstruction was recently published (Straume 2020), there remains high uncertainty regarding temperature and aridity, for example. We now discuss uncertainties related to input landscape in the main text (lines 590-608) and Note S1. Exploring further models with multiple reconstructions, such as those accounting for continentality and air moisture in lapse rate methods (Romshoo, Rafiq and Rashid 2018, Spicer 2018) is an important agenda for future studies. These limitations are better presented in the revised discussion, where we call for future reconstructions and, instead of presenting a paleo-reconstruction, we focus on presenting a simulation engine that can handle multiple reconstructions.

*    Minor note: reference 3 and 4 seem to be the same in note S2.

14.    Line 407: should read as 'flexibility'

**Response:** Thank you for spotting this typo. We corrected accordingly.

15.    The authors should examine the example code associated with the 'gen3sis' R package. The resulting map of alpha richness does not match empirical patterns very well, and the authors may want to consider a new example.

**Response:** In the vignette in the previous manuscript version, we simulated biodiversity from 150 Ma until 100 Ma. Nevertheless, your comment made us realize that this should be improved with a more intuitive example that also corresponds better with the case study presented in our manuscript. We revisited our vignette and now use a landscape for South America 1d that presents biodiversity patterns that correspond better with empirical patterns. Thank you for your constructive suggestions.

## Reviewer #2:

This study presents a new R package for conducting simulations of eco-evolutionary dynamics to explore biodiversity patterns. The authors argue that a reliance on statistical and verbal models lacking mechanism has hindered progress in biogeography and macro-ecology and that computer simulations, in which these mechanisms can be encoded, can provide new insights into the causes of variation in biodiversity over time and space. The paper describes a general simulation model aimed to address this, that includes various processes such as dispersal, allopatric speciation, trait evolution and extinction, that take place on a dynamic landscape. The paper is well explained, the structure of the model is simple to follow and overall seems sensible. Two case studies are provided to illustrate the applicability of the model. The first, demonstrates that when island area rises and falls over time, this leads to a hump shaped trend in species richness, that is sensitive to how rapidly
niche related traits evolve. The second, shows that when warm-wet environments have a higher individual carrying capacity, this leads to a latitudinal gradient in species richness.

I agree with the authors that these kinds of mechanistic simulation models are an additional useful tool in understanding biodiversity patterns. Researchers have been advocating the use of such models for many years and there have been a number of high profile studies implementing this kind of approach. I have to admit, however, that I struggled to identify in this paper what the key advance or new insight is over these earlier mechanistic modelling studies.

**Response:** First, thank you for your positive comments. We are glad to see that our efforts to explain a quite complicated model with many modules and interacting processes payed off. We do, however, regret that we did not succeed in illustrating its novelty and utility as a general “tool” for future biodiversity research. In an effort to better illustrate the advance that gen3esis could facilitate, we have made substantial changes to the manuscript. In particular, we now review several of the currently available and relevant population level, spatially explicit eco-evolutionary models. With this, we assert firmly the advances of our modelling approach, which we developed exclusively for its potential to include multiple custom processes. We argue that gen3sis facilitates scientific advancements in the field partly because it is open source and has well-documented code. Furthermore, by focusing more exclusively on the second case study, we provide new insights on the mechanism behind the LDG, as well as other large-scale biodiversity patterns. We hope that our efforts to better relate our study to the literature and to go more into depth in our case study now provide a convincing argument for the novelty of our approach.

While as a modeller, I remain hopeful and open-minded, I am also sceptical that the general approach advocated here, will address the challenges to the field that the authors highlight. The problem, is that with these increasingly complex models the number of free parameters or hard encoded processes becomes enormous.

**Response:** We fully agree that model complexity and the number of parameters is a challenge in any modelling approach. This point also relates to a continuous debate on the utility of simple vs. complex models. We do not wish to go into depth about this debate in our study but, with this in mind, we developed an engine that allows the user to define the final number of free parameters. We now discuss this better in the model description, providing examples of the simplification or complexification of processes. This allows the user to set the complexity and potentially increase it gradually as understanding progresses. We now showcase three different model configurations with different implemented parameters to illustrate how such analyses can be conducted (lines 119-126, 347-366, 390-429).

Often the models are too complex to adequately explore parameter space and to fit to empirical data. As a result, earlier mechanistic modelling studies have resorting to picking a particular set of model assumptions and simply showing that this can produce realistic looking patterns but without formal hypothesis testing or parameter estimation. For instance, in the current study, the authors show that when warm-wet environments support more individuals then a latitudinal diversity gradient emerges. This is expected and of course not a new finding (e.g. Hubbell's 2001 neutral theory shows that metacommunities with more individuals contain more species), but more
importantly, it doesn't provide much additional insight into whether this mechanism actually contributes to causing the LDG.

**Response:** Thank you, we fully agree. The main goal of the manuscript was to present the engine with a few case studies as mini examples. However, by doing this we did not fully explore or show-case the use of this engine to the community entirely. We have now expanded the LDG case study considerably to tackle this problem. This makes the main case study presented in the manuscript unique regarding not only the methods presentation but also the insights into the mechanisms behind the formation of the LDG.

The problem comes back to the fact that there are multiple explanations for why richness varies with gradients in temperature and water availability and these causal hypotheses are extremely difficult to test because they all lead to the same prediction: that richness is highest in warm-wet environments. If you look at disciplines such as climate science, which the authors point to as an example of where mechanistic models have been extremely successful, the reason these complex models (e.g. GCMs) work is because we have a very good understanding of the basic physics and chemistry. Without a more thorough understanding of the mechanisms involved in generating and maintaining biodiversity, and without independent parameter estimates for most of the key processes, it is difficult to see this approach leading to major advances. This is a general criticism of the approach, and one not limited to this specific study, but I failed to see how the authors propose to address this problem.

**Response:** While we agree with the sentiment of this comment, we do not see this as discouraging for the research programme of building mechanistic models (see also our response to a similar comment of Rev 3 below), but rather as an indication that more research and more tools are needed to overcome these challenges. This is what we propose in our manuscript and with the presented approach.

We argue that all underlying processes are also not understood exactly in climate models (e.g. cloud formation), and that we do know something about the processes that determine biodiversity dynamics. We know, for example, that there is speciation, extinction, dispersal and population dynamics, and we have a rough idea that it is more difficult to disperse from Europe to Australia than to Asia. We also have a rough idea about environmental conditions, as well as plate tectonics and the shape of continents during the time evolution took place.

The details of these processes, however, are comparatively less understood than in climatology. And this is one of the reasons why we need eco-evolutionary computer models, and why we must use them differently than in climatology. GCMs are built to PROJECT the consequences of known processes into the future, while our model is built to EXPLORE the consequences of alternative hypotheses and compare them to empirical data, to find out how biodiversity dynamics work.

We heartily agree with the reviewer that the latter will be an extremely challenging task, but on the other hand, we fail to see a better option for the field. High-level data analyses of phylogenies, for example, will not tell us much about the processes either, and we have very few possibilities to observe them live or manipulate them via experiments, so attempts to see if certain process combinations robustly excel in producing the observed patterns seems the best option to us to find out what the truth may be. We appreciate that the reviewer raises several of the main challenges that we as modellers need to consider. These issues can be discussed and debated at length, but here we will highlight three non-mutually exclusive factors (model complexity, validation, and multiple processes) and relate each of them to the utility of gen3sis.

First, as the reviewer mentioned, the issue stems partly from the degree of complicity of the models we analyse. A “simple” model may be “simple” to analyse analytically or numerically and our understanding of the behaviour of such models may be relatively robust and rigorous. However, nature is rarely simple, and to model the complex nature of, for example, the drivers and processes underpinning the LDG, we need to expand on model complexity. The outstanding question is how such mechanisms/ complicity should be formulated and implemented. The question may be impossible to answer, but a strategy that we propose is to focus on mechanisms in isolation first and then implement them in concert. In doing so we may be able to better understand the individual mechanisms, as well as the interaction between them. Gen3sis allows for such progression in complexity, as it is built in a modular way where processes (and thus complexity) can be switched on and off. We now discussion this in the manuscript (lines 100-102, 115-117, 119-126,128-136 and section ‘core functions and objects’), as well as in the new Figure 3, Note S1 and Table S1.

Second, the review rightly raises the issue associated with parameter estimation and model validation. Indeed, simple models or models of particular processes may be easier to estimate parameters for and to validate. Gen3sis does allow for such a reduction in complicity and a focus on particular processes. In doing so computational expenses are reduced and the data may be available such that fitting the model to data is possible. Again, reducing the complexity of gen3sis and fitting sub-processes to data does not necessarily help us to understand the full complexity of biodiversity and diversification processes. We thus argue that more complex models also need to be investigated. Gen3sis also accommodates such complexity. Indeed, complex models are difficult to fit, partly due to increased runtime of the model but also due to limitations in the data required for parameter estimation. However, given the development of computational power, increased data availability and novel statistical approaches may allow for model validation of complex mechanistic models in the near future.

Third, we agree that a particular pattern (e.g. the LDG) can be explained by multiple processes. This essentially follows from the fact that the biological patterns that we observe tend to be relatively simple and do not necessarily contain signals from the many complex processes that underpin the pattern. Despite this discrepancy in the complexity in patterns and the underlying processes, or maybe even because of this discrepancy, we argue that theoretical investigation of the link between processes and pattern has great value. We see value in examining such multiple explanations in theory, as they can inspire additional model development, data collection, experiments and ultimately explicit hypotheses. Gen3sis enables such an exploration of processes. By distinguishing processes that give similar patterns from processes that give contrasting patterns, we can also start to sort empirical findings into categories. Such sorting can improve our overarching understanding of the patterns that have intrigued biologists for centuries. We attempted to better demonstrate this point with our improved case study.

I think the R package that the authors have developed would be a useful tool that I am sure ecologists would employ. Whether it really provides a 'general' model as the authors claim I'm not so sure. I think every ecologist will have different opinions about how a particular process should be modelled, or requirements for what should or should not be included in the model.

**Response:** Thank you. We wish to emphasize that we claim a general model in the sense of flexibility of process implementation (this distinction is now made on lines 114-117). The different opinions about how particular processes should be modelled is the driver of making gen3sis a flexible tool. We thus claim that gen3sis allows the users to decide “what should or should not be included in the model”. The way such processes are modelled is a different issue, but we expect that gen3sis will stimulate ideas, including those on alternative ways of implementing such processes in future models, shedding light on the processes related to emerging biodiversity patterns.

For example, I was not convinced by the first case study on island dynamics presented in the paper, showing that with faster rates of niche evolution, species diversity is expected to decrease. This arises in the model because species that evolve rapidly may evolve to have niches that do not correspond to climate conditions on the island. But it is hard to see how this would actually happen in nature and it seems highly unrealistic: if the climate affects individual survival, then species would adapt to the conditions they encounter on the island and not just randomly drift through niche space. With faster rates of niche evolution species would be able to better track changing climate conditions and thus avoid extinction, leading to higher diversity.

**Response:** We agree with you that empirical parallels are harder to interpret in this theoretical case study. Therefore, we decided to move it to the Supporting Information and focus on the more realistic second case study. We agree that the evolution of maladaptations to conditions not present in the environment is unrealistic, but this emerges from the experiment’s assumption of trait evolution as a global parameter, so much so that we had to vary trait evolution relative to environmental variability. A more realistic scenario would be to make the model more complex and implement species-specific trait evolution (i.e. making trait evolution itself evolvable) and/or environment-driven trait evolution (e.g. higher at higher temperatures). Doing so, we might observe what the reviewer indicates, that species and their trait evolution parameter get selected by the environmental dynamics. This would also avoid the necessity of having to simulate scenarios of different relationships between trait evolution and environmental variability. This perspective is now included in Note S2.

One suggestion, would be for the authors to expand the scope of their experimental simulations. For instance, to simulate the evolution of biodiversity patterns under a particular set of model assumptions and parameters (stage 1). Then to employ a formal model fitting exercise where they run multiple simulations under different assumptions and parameter combinations and test the fit of these different scenarios to the patterns produced in stage 1. Testing how often the true model is recovered (type 1 and type 2 errors), how accurately and reliably the true parameter values can be estimated, and how these inferences depend on different assumptions would be an important first step to demonstrating the model is potentially useful. In the context of the current study, if the authors could demonstrate that richness correlates with energy-water availability because of differences in individual carrying capacity, rather than other prominent explanations (e.g. niche conservatism, niche partitioning etc) then this would be much more compelling.        
**Response:** This is an excellent idea and we are working on it on multiple fronts. Although such an experiment would be too big to include in this paper, we decided on a compromise where we implement and test three models. M0 acts as a baseline scenario based on recent evidence, while M1 and M2 implement alternative hypotheses for the LDG that operate over very distinct processes. In this study, we focus on presenting an engine that would allow such exploration and we now make it clear in the text what the next steps are. Thank you for your thorough and insightful review.

## Reviewer #3:

This is an interesting and well-written presentation of a new r package for ecological modelling. The goal of the paper is worthwhile - to create a general modular software framework for deeper integration of models in geographical ecology to improve theory and make it more explicit.
**Response:** Thank you.

It's a little bit hard for me to tell whether the authors actually reach this worthy goal. They argue that fields like cosmology and climatology are heavily reliant on mechanistic models, which they are, but those models seem very different. In cosmology everything is highly deterministic and fully specifiable once you know the initial conditions, a modeller's dream. And climate modelling uses a small number of huge models, greatly dwarfing ecological parallels such as the Madingley model and clearly completely different from what is offered here. The premise that cosmology and climate uses models and so should ecology thus seems superficial and in my opinion doesn't add theoretical depth.

**Response:** We appreciate the comment. We argue that even cosmology and climate simulations are far from “ideal” (practical simulations of these systems typically require numeric approximations, i.e. discretization, that leads to various issues and uncertainties). However, we agree that they are sufficiently different in their nature, making a direct analogy inappropriate.

Moreover, we did not intend to convey that the cosmology / climatology point is a crucial motivation for the research programme that we propose here. We think the motivation stands and is formulated in the literature of the field (as we already detailed in the introduction). The fact that other fields have already successfully conducted such research programmes just offers additional cause for optimism.

We see, however, that the prominent placing of this point may have given the thought undue emphasis. We have therefore shortened the comment and moved it to the conclusions / discussion, which makes it clear (in our opinion) that this is not a premise, but rather serves as secondary support for our argument that we should build mechanistic models.

My main question is what this model is for. Is it a toy program that can be used for teaching? Is it a fully finished model? The authors cast it as a "modelling engine with a modular implementation", indicating that this is something that other people can hook up to their code, but how exactly they would do that is not clear.

**Response:** No, we definitely do not see this as a toy or teaching program, but as a simulation model that can and should be used to test hypotheses about the origin of biodiversity patterns against data. We largely improved our case studies to better demonstrate how it can be used to better understand the mechanisms relating to the origin of biodiversity.

By "modelling engine with a modular implementation", we meant that the program is coded in a way that subprocesses can be changed (note that the code is open source). This is important because, although we offer many options to change the process assumptions already, it seems likely to us that researchers will want to use our platform to make specific changes to the code in the future. However, even as it is, we see gen3sis as a serious attempt (and possibly the most complete so far) to simulate the emergence of global biodiversity patterns.

We see our engine as a useful modelling framework for advancing biodiversity research. While this tool also has teaching potential, our main goal is to offer a platform that facilitates model implementation and comparison. More specifically, we see gen3sis as a tool that enables analyses of progression in model complexity and process interactions, as it is built in a modular way where processes (and thus complexity) can be switched on and off. Gen3sis allows a reduction in complexity and thus facilitates a focus on particular processes. In doing so, computational expenses are reduced and the data may be available such that fitting the model to data is possible. On the other hand, we argue that more complex models also need to be investigated. Gen3sis also allows such complexity and can thus be used to combine studies on isolated processes, as well as the interaction of processes. On a similar note, theoretical investigation of the link between processes and pattern has great value in our efforts to understand how multiple processes can lead to similar observations in data. This issue stems from the fact that the patterns that we observe in nature tend to be relatively simple compared to the cast complicity of the mechanisms and processes that underpin them. We thus see value in examining such multiple explanations in theory, as they can inspire additional model development, data collection, experiments and ultimately explicit hypotheses. Gen3sis enables such an exploration of processes and, by distinguishing processes that give similar patterns from processes that give contrasting patterns, we can also start to sort empirical findings into categories. Such sorting can improve our overarching understanding of the patterns that have intrigued biologists for centuries. We now provide more examples of how to implement specific models, and we showcase this capability with a more complete case study.

Is it really a module that can be tweaked and changed and incorporated into other people's research projects? Or really just a very customisable final model?

**Response:** We see gen3sis as a customizable modelling platform because not only parameters but also processes can be customized. Although this is a more philosophical perspective concerning boundaries between different models or different parameters, we did our best to maximize process implementations, especially when designing model core and input values. Although those are inside a framework, all currently proposed LDG hypotheses (Pontarp et. al. 2020) could be readily implemented with gen3sis.

There's a mention of the flexibility coming from functions being definable in an open way, but no example of this (that I can see). As an editorial comment, I don't think the code examples should be relegated to the supplement in a paper like this - let's see the package in use in the main text.

**Response:** Thank you for this comment. We now provide much more clarity on customization possibilities. We further opted to provide additional customization examples in the vignette of the R-package to maintain the flow of the manuscript.

This question is especially pertinent given that, as they state, "a mechanistic understanding" in ecology "is elusive". Do we really know enough about the mechanisms to say that the implementation choices taken here are sensible?

**Response:** We know the rough causal structure of the mechanisms, i.e. "a mechanistic understanding", that determine biodiversity dynamics; e.g. which types of speciation mechanisms exist. However, we do not know how to best describe these mechanisms in a formula, what factors they depend on, and what their parameters are. This is the main motivation for providing such a flexible simulation engine. In our case study, we now use and discuss the approach of a baseline scenario that we assume to underlie all further implementations. Although we are still far from achieving such an understanding, we think that this engine could be a useful contribution. One way of doing so may be to use gen3sis to contrast processes in isolation but also to combine processes. We now present this motivation more clearly in the revised manuscript (lines 41-126, case study and discussion ).

The authors state in the abstract that the model produces "realistic island biodiversity dynamics", but how do we know that the emerging dynamics are realistic? How much of the model framework is amenable to modification, and what elements are taken as assumed? I can't see that distinction made.
**Response:** Thank you for pointing out this omission. We understand the reviewer’s concerns. This case study was removed from the manuscript and improved in Note S1, so that the realism of the emergent patterns should be clearer now. We define “realistic emerging dynamics” as those that follow the predictions of the General Dynamic Model (GDM) of Island Biogeography, which itself has been largely tested, both with empirical data and with a few different mechanistic models, specifically a GDM-predicted hump-shaped diversity time-series (Whittaker et al. 2008; Borregaard et al. 2015, 2016). Further model and experiment extensions that we assume are the most interesting to modify (e.g. making trait evolution specific to individual species or environments, adding habitat heterogeneity or spatial/temporal autocorrelation) are now also indicated as future possibilities. We reformulated entirely the section ‘core functions and objects’, providing non-exhaustive customization possibility examples (lines 208-316). The main case study also serves to show the versatility of the engine (lines 119-124, 390-432, Note S1, Figure 3 and Table S1).

I have a few other implementation-based questions. It appears that "runtime critical" elements are implemented via RCpp, but looking at the github code, it's actually a fairly small part of the code that is written in C++. Is that really enough to make the model run fast?

**Response:** The balance between code speed and readability was constantly re-evaluated during the implementation phase. Most of the model was developed in R. This concerns in particular the interface, which is important to keep in R to reach a broader scientific audience, but also parts such as data transformations and certain parts of the main modelling loop, which are not runtime critical.

However, “fast” always has to be taken with a grain of salt, especially in R (see the answer after this one on runtime). That said, the clustering and distance calculations (or rather lookups of the precomputed/cached distance calculations) are central components that are used multiple times during each step and for each species.

The clustering was originally implemented in C++ when the previously used library was discovered to be buggy, and this implementation has been used ever since. Clustering is a critical operation that is used for each species in both evolution and speciation operations at every time-step. Having our own implementation also allowed us to easily implement a more randomized version of the algorithm in which each cell can have a different radius for what is considered to be in reach.

The landscape distance matrices are a trade-off between storage size and computation time. As the site to site distance is used many times for different calculations (clustering, dispersal), pre-computing them saves a significant amount of time. Unfortunately, these full distance matrices (cell x cell) scale with the power of four with respect to the input resolution, up to the point where the full input size for a 1000 time-step full world at 1-degree resolution reaches a size of 1TB. Storing only the local distances, that is the distances between each cell and the adjacent neighbours, scales linearly with the number of cells (i.e. 8*#cells), or quadratic with respect to to the input resolution. But this comes at the cost of having to recalculate the inputs during each individual simulation.

We have implemented a slightly modified variant of Dijkstra’s shortest path algorithm. In addition to finding the shortest distance to each cell, we added an early termination criterion where we only consider cells within a (configurable) maximum distance. When that distance is set to infinity we achieve a roughly similar performance to the costDistance function in the gDistance package (which uses the c++ igraph library internally). When we lower this distance we can terminate the distance calculations early and achieve speedups of up to 10x (achieved on a 1d worldwide input with a distance limit of 3’000 km).

There are many opportunities for optimizations remaining within the code, from parallelization to sparse data structure, or even switching out cell-based data structures for polygon representations (and many more). For now, though, we have focused on the readability and maintainability of the code over implementing all desirable optimizations (of course time, budget and manpower restrictions apply here as usual).

There are no runtimes given for the examples, which I guess is fair enough given the diversity of hardware, but would be nice to get an indication of how fast it is and could be made. I'm here assuming that the code does not call into any C++ libraries not included in the repo (it doesn't look like it).

**Response:** That is correct. We currently run on a small heterogeneous cluster with AMD Opteron (6134, 6238, 6274) and Intel Xeon Gold (6134, 6234) CPUs with one simulation per core. The Opterons are generally around four times slower than the Xeons. Runtimes for the simulations in the case studies run anywhere from a few minutes to two days for a single simulation. It is heavily dependent on the number of species emerging during a simulation and their geographical extent. Thus, runtime is highly dependent on the assumed model and input landscape.

Currently, the (direct) use of external cpp libraries is limited to two parts of the boost library for the distance calculations (arrays and the Fibonacci heap). The current state of optimizations is limited because no parallelization is implemented, and we have prioritized ease of maintenance and development for this initial release. The runtime is more or less evenly distributed between the different parts, with no single bottleneck dominating the runtime.

Finally it would be nice to see some more expanded documentation for using the package. There are vignettes available, but are they sufficient to run the software for other people?

**Response:** We think that the help files and the vignette are the appropriate places for the package documentation, given that the present manuscript is already quite long. It would be uncommon / not recommended for an R-package to provide extensive documentation outside the help or vignette. Thus, the question is if our current help files and vignette are sufficient for outside researchers to work with the model.

While we think that the vignettes do provide a basic level of guidance for new users, there is of course always room to improve documentation. Therefore, we revisited our entire vignette and documentation and implemented feedback from the reviewers and new gen3sis users. We are confident that the current vignette and R documentation, along with this manuscript, will provide new users with the necessary support for a “not-too-steep” learning curve.

In conclusion, this is potentially interesting, but I don't at present feel I have sufficient information to evaluate whether the authors achieve what they promise with the package.

**Response:** Thank you for your comments. We hope to have addressed your concerns in our newly revised manuscript, which includes substantial additional content.
