# Supplementary material for: gen3sis: A general engine for eco-evolutionary simulations of the processes that shape Earth’s biodiversity
Source: PLoS Biol. 2021 Jul 12;19(7):e3001340. doi: 10.1371/journal.pbio.3001340 (PMC8384074; doi:10.1371/journal.pbio.3001340)
Supplement: Attachment — Submitted filename: Response_Reviewers_gen3sis_R2.docx [file pbio.3001340.s024.docx]

## *****************************************************

## EDITOR COMMENTS:

Thank you very much for submitting a revised version of your manuscript "gen3sis: the general engine for eco-evolutionary simulations on the origins of biodiversity" for consideration as a Methods and Resources paper at PLOS Biology. This revised version of your manuscript has been evaluated by the PLOS Biology editors, the Academic Editor and the original reviewers.

You'll see that while reviewer #3 is now satisfied, reviewers #1 and #2 continue to raise some significant concerns with your treatment of the literature (and specifically the novelty of your approach with respect to other studies, such as Rangel et al, Saupe et al), with the novelty and strength of your LDG case study, including the parameters and scenarios explored. The Academic Editor asked me to emphasise the need for you to better explain how the resource builds on existing work in the field.

**Response:** Yes, we agree with your summary. We broadly separate these concerns into two areas: 1) is the engine / approach novel 2) are the case study results credible / reliable.

About the first aspect (novelty of the software): First, we would like to stress that we see this as the main point of our paper, as indicated by our title “gen3sis: the **general engine for eco-evolutionary simulations** on the origins of biodiversity”. The reviewers insist that there have been previous, similar models, and point in particular to papers by Rangel et al. However, we would respectfully point out that Thiago Rangel is a coauthor of this paper, so we are very much aware of his work (which we acknowledge duly), of his collaborators, as well as the differences between models. As the title says, gen3sis is designed as a general engine for global macroevolutionary simulations, with flexibility and modularity to be applied and modified by the community. Conversely, all papers by Rangel and his collaborators using simulations used very specific compiled software, which were never made available as an R package, and are not used by other researchers. In principle, it would be possible to re-program the software implemented by Rangel to become a widely distributable general purpose package for simulation of macroevolutionary processes, but we are the first to do so and this is the emphasis of this paper. In fact, the proposed flexibility and modularity of gen3sis allows for other models to be re-implemented within the framework of gen3sis engine. So, if the reviewers claim that gen3sis is not novel, then we would like to know of any existing platform that accommodates spatially explicit macroevolutionary simulations at the global scale, in a single, openly accessible and modifiable framework, conceived for generality and flexible modelling.

About the second point (case study): We agree that the case study must be credible, and we have made various modifications and clarifications. We added more models, all of those with and without deep-time environmental dynamics. Specifically, we added a null model as requested by reviewer 1. We added several new summary statistics, parameter explorations and selection criteria. We do, however, stress that our original intent with this case study was not to make a conclusive statement about the cause underlying the LDG, but to illustrate the potential use of the gen3sis model. Nevertheless, we did extend the case study in response to reviewer comments in the previous revision, and we addressed remaining LDG concerns (as detailed below). Our illustrative case study largely expanded in response to the reviewers’ comments, and expanding more would be beyond the scope of the paper, which was primarily to present our new method. We nevertheless propose avenues for future research targeting explicitly LDG in much detail and we showcase and better contextualize how gen3sis can be used to support such research, advancing our mechanistic understanding of biodiversity.

In light of the reviews (below), we will not be able to accept the current version of the manuscript, but we would welcome re-submission of a much-revised version that takes into account the reviewers' comments. We cannot make any decision about publication until we have seen the revised manuscript and your response to the reviewers' comments. Your revised manuscript is also likely to be sent for further evaluation by the reviewers.

**Response:** We thank the editorial board for the possibility to re-submit our revised work. We fully replied to all the comments and we are looking forward to your decision.

**IMPORTANT:** I should also say that we will be willing to consult reviewers only once more on this manuscript, and if they remain unconvinced by the merits of your study after the next resubmission we would not invite you to revise again.

We expect to receive your revised manuscript within 3 months.

**Response:** Thank you for this review and for allowing another round of interactions. We appreciate all the reviewers’ comments and that they recognized that the additional work substantially improved our initial version. We are keen to have this study published at PLOS Biology.

## *****************************************************

## REVIEWERS COMMENTS:

**Reviewer #1:**

In general, I found the manuscript much improved. The R package will be useful to the evolutionary and ecological communities, although I disagree with the authors regarding its novelty. To me, the power of the R package is providing an easy-to-use framework that is flexible with appropriate vignettes explaining the parameters. As currently written, the authors (perhaps inadvertently) downplay the novelty and significance of previous, similar platforms.

**Response:** Thank you for the positive feedback. We agree with these advantages of an R package, and we made them clearer at the main text without downplaying the novelty of previous similar platforms. Nevertheless, we are still not aware of a platform readily capable of integrating the same amount of ecological and evolutionary processes in such a flexible manner (opening up so many process interaction possibilities) that can be readily used for a variety of input landscapes. We refer to recently developed or in development models translated into an R package (lines 154). We expanded the review and made this point clearer in the revision (lines 89-93, 100-106, 111-117, 365-370).

We also cite the previous literature with similar methods including Qiao 2016, Saupe et al. 2019ab and 2021 and Rangel et al. 2018 as well as our own previous work (Leprieur et al. 2016, Descombes et al. 2018). Nevertheless, none of those previous studies proposed a flexible approach with the scope of exploration as proposed in the present study. We clarified this point in the revised text (lines 102-106).

[References]_________________________

Qiao H, Saupe EE, Soberon J, Peterson AT, Myers CE. Impacts of Niche Breadth and Dispersal Ability on Macroevolutionary Patterns. Am Nat. 2016;188(2):149-62. Epub 2016/07/16. doi: 10.1086/687201.

Saupe EE, Myers CE, Peterson AT, Soberón J, Singarayer J, Valdes P, et al. Non‐random latitudinal gradients in range size and niche breadth predicted by spatial patterns of climate. Global Ecol Biogeogr. 2019;28(7):928-42. doi: 10.1111/geb.12904.

Saupe EE, Myers CE, Townsend Peterson A, Soberon J, Singarayer J, Valdes P, et al. Spatio-temporal climate change contributes to latitudinal diversity gradients. Nature Ecology & Evolution. 2019;3(10):1419-29. Epub 2019/09/11. doi: 10.1038/s41559-019-0962-7.

Saupe EE, Qiao H, Donnadieu Y, Farnsworth A, Kennedy-Asser AT, Ladant J-B, et al. Extinction intensity during Ordovician and Cenozoic glaciations explained by cooling and palaeogeography. Nature Geoscience. 2020;13(1):65-70. doi: 10.1038/s41561-019-0504-6.

Rangel TF, Edwards NR, Holden PB, Diniz-Filho JAF, Gosling WD, Coelho MTP, et al. Modeling the ecology and evolution of biodiversity: Biogeographical cradles, museums, and graves. Science. 2018;361(6399):eaar5452. doi: 10.1126/science.aar5452.

Leprieur F, Descombes P, Gaboriau T, Cowman PF, Parravicini V, Kulbicki M, et al. Plate tectonics drive tropical reef biodiversity dynamics. Nature Communications. 2016;7:11461. doi: 10.1038/ncomms11461.

Descombes P, Gaboriau T, Albouy C, Heine C, Leprieur F, Pellissier L. Linking species diversification to palaeo-environmental changes: A process-based modelling approach. Global Ecol Biogeogr. 2018;27(2):233-44. doi: 10.1111/geb.12683.

Most of my questions regarding the framework and its flexibility were clarified by the authors. They do a nice job explaining the various parameters in Gen3sis, and I think the algorithm will be of interest to the community.

**Response:** We appreciate the encouragement and we hope that now the improved manuscript is acceptable for publication. Thank you very much for your comments.

However, and apologies if I missed this, it was still unclear to me how extinction occurred. I imagine extinction could occur via numerous routes, and this should be made clear.

**Response:** We thank the reviewer for requesting further clarification. We clarified the extinction processes in the main text (lines 128-136, 210-214, 245-248, 306-307) and in the legend of Figure 2 of the R1 version. Extinction is an emerging event by default in gen3sis, caused by the (gradual) loss of all grid cells inhabited by a species. That can happen either when grid cells become uninhabitable (i.e. matching between input landscape and the species’ environmental preferences) or the abundance is reduced to zero (e.g. due to competition or niche filtering at the ecology function). In the revised manuscript, we better explained how extinction occurs in a fully dedicated paragraph (lines 227-233).

I still found issues with the main LDG case study provided by the authors. Although I understand the authors provide the case study as an example of how the modelling framework operates, they also present the results as if the study is novel with conclusive findings. This is problematic, as the LDG study is not novel (the same question has been examined using a similar framework by numerous authors) and is missing key elements, which are discussed below. The authors would have convinced me of the power of their framework and the use of LDGs as a case study if they had evaluated more than three hypotheses and included null models. However, this was not done, even though the authors cite this functionality as one of the primary novelties of their framework (and it was requested by the reviewers previously).

**Response:** This excellent comment made us realize that there was a lack of clarity with the way we presented our case study. Thank you! In the previous revision, we added a simple model with the purpose of showcasing the flexibility and utility of our model and R-package. The reviewers also seem to recognize the utility of such a general approach. We do, however, agree that this did not correspond to a true null model. In the revised manuscript, we now include one null model and one additional alternative model, both with and without varying environmental and geomorphological dynamics, for a total of 5 models tested:

Null model: In the model **M1**, we present a simple model where all the terrestrial cells are ecologically equivalent, so that temperature and aridity do not determine the presence or abundance of the species. Hence, ecology can be considered as turned off. We explore a range of parameters of dispersal and speciation that are now used throughout all simulations.

In addition, we now run a new ecological model **M4.** In this model, we apply a carrying capacity, but the carrying capacity does not vary according to temperature and aridity. We further explore several values of carrying capacity. This corresponds to a null-model to contrast to the previous model M2 (now **M5**), where the carrying capacity varies with temperature and aridity.

Additionally, we run all the five models with and without deep-time dynamics (lines 420-422). This was done by running all models while keeping the landscape constant (i.e. no climatic/environmental dynamics and no geomorphological dynamics) with the same number of time steps as in the dynamic landscapes (L1 and L2). We show that those have a systematically lower agreement with the data (lines 551-553).

We further show that the null models do not reproduce the LDG suggesting that realistic eco-evolutionary processes are necessary for this pattern to emerge (see Figure S4 for example). We thank the reviewer for this suggestion on the null models, this helped illustrate a key message of the study with a much more robust case study.

1. Hypotheses:

a. The presentation and description of hypotheses requires further thought. The authors focus on three hypotheses: time for species accumulation, diversification rates, and ecological limits.

**Response:** Due to space limitation, we agree that the presentation of the hypotheses was too succinct and those could have been better connected with the literature. In the revised manuscript, we better explained the hypotheses (line 370-391, 430-475, Note S1).

The 'time to speciation' hypothesis is purposely vague in the literature because it is difficult to disentangle the various processes that could produce higher species richness at low latitudes using empirical data. However, a mechanistic model allows for these processes to be examined explicitly. Speciation, extinction, and dispersal are the only mechanisms that directly generate differential patterns of biodiversity across landscapes: how might these three processes contribute to greater species richness in the tropics given an older tropics, and which are supported by the M0 model? Usually, the 'tropics as older' hypothesis infers extinction rates were higher at high latitudes due to climate change (especially in the Northern Hemisphere), but all three could be involved (or not).

**Response:** The reviewer is correct, the “time for speciation” is vague in the literature and might actually not be the correct terminology for the mechanistic model. In this implemented hypothesis, the main baseline constraint is that the tropical environment is older and this could have favored both higher time for speciation and lower extinction. Hence, in response to this comment, we first renamed this hypothesis as “niche conservatism”, where the hypothesis name does not explicitly assume that it is speciation or extinction leading to the LDG. Second, with the mechanistic model, we analyzed emergent speciation and extinction rates within low latitude [23° 27' N- 23° 27' S] and high latitude [66° 33' N -23° 27' N; 23° 27' S-66° 33' S]. We show that all three processes are involved, as suggested by previous studies (lines 559-571, Figures S10, S11, S12, Table S5, S6).

b. Regarding the carrying capacity hypothesis: the authors should discuss (even briefly) why higher abundance may lead to higher species richness. I understand that higher abundance would lead to more biomass, but not necessarily how it could lead to greater species richness. It is interesting to test this hypothesis in the Gen3sis framework, but the authors need to better explain the mechanics, as this is the key contribution of their modelling framework.

**Response:** Thank you. We agree and now added a brief explanation on the main text where the process is clarified (line 461-468 and Note S1). The carrying capacity will limit the number of individuals of the entire local community in a site. The number of individuals is decreased randomly across different species until carrying capacity k is reached. Remember that species abundances are dictated by the ecology function. The number of species loss is the number of species with zero individuals at time t1- t0 (Note S1).

Again, the only processes that can produce more species in a region compared to another region are differential rates of dispersal, speciation or extinction. Thus, how might carrying capacity generate differential rates of speciation, extinction, or dispersal? Do the authors suggest that lower carrying capacity will increase extinction of incipient species at high latitudes? Or, do the authors suggest that speciation and extinction rates are constant across latitudes, and the higher carrying capacity contributes to higher diversity in the tropics via dispersal of species into the tropics? Again, you found this model to be the best supported, and some discussion of how this occurs in the simulation - the mechanisms - is warranted and would be of great interest to readers.

**Response:** Thank you for bringing the discussion to the underlying mechanisms rather than to parameters. The carrying capacity will limit the number of species that can co-exist in a given cell, which will interact with the processes mentioned by the reviewer. To tease apart how varying carrying capacity along a temperature and aridity gradient interact with dispersal, speciation and extinction to generate different pattern of species richness along latitude, we compare the simulation to a null-model with a fixed carrying capacity, which does not vary along abiotic gradients. We then analysed emergent values of speciation, extinction and immigration rate (calculated as number of events per 1 myr), and show that speciation and extinction rates were consistently higher at low latitude than at higher latitude with a similar macro-dynamic curve when no deep-time environmental dynamics is considered (Figure S10). Including a carrying capacity leads to a characteristic increase in speciation and extinction rates towards the present, which intensifies when temperature and aridity are considered as carrying capacity drivers (Figure S10, Figure S11, Figure S12). Dispersal contribution from the low latitude to the high latitude (percentage and standard deviation: M1=0.57 ± 0.19, M2=0.67 ± 0.18, M3=0.45 ± 0.17, M4=0.72 ± 0.11, M5=0.54 ± 0.21) was always superior than from high latitude to the low latitude zones (M1=0.07 ± 0.02, M2=0.1 ± 0.02, M3=0.07 ± 0.03, M4=0.05 ± 0.02, M5=0.11 ± 0.04). This contribution disparity was lower on models not considering deep-time environmental dynamics, as expected due to the climate change driven extinction events (Table S5, Table S6). We added this to the text (lines 559-571, Note S1) .

c. Assumptions are provided for each of the models/hypotheses (M0, M1 and M2) in Note S1, with the exception of Model 0. Model 0 does not seem to be a true null model, and the assumptions should be specified. Indeed, proper null simulations are not provided by the authors, which would allow for the effects of certain parameters on patterns to be examined (e.g., absence of speciation, extinction, ecology). The authors mentioned null simulations are feasible (for example, in Note S1 'mechanisms can be completely turned off. In the case of ecology, this will lead to an ecologically neutral model'), and I was therefore surprised to see they were not performed. E.g., do flat LDGs result if climate does not change? If extinction or speciation is prevented, but dispersal allowed?

**Response:** We agree that using a set of null models allowed us to better explain our results. A null model without speciation would be trivial since there would be no species emerging. We could start the simulation with many species and not speciation, but in that case, no phylogeny will be generated and therefore this model will fail since it is not fitting the data with a real diversification history. Extinction is not a process that can be controlled since it is a direct consequence of habitat change, evolution and dispersal, hence removing extinction is not possible. More about extinction mentioned previously. If one is interested in manipulating the values directly, one could use a much simpler model in which one can impose these different rate values to the tropical-temperate system (Etienne et al. 2019). Nevertheless, we added several null models/alternative in order to better evaluate the contribution of ecology/evolution and environmental dynamics in the generation of biodiversity patterns including the LDG (i.e. M1 and M4 and the removal of deep-time dynamics). Those are presented in the revised case study, and better discussed in the revised discussion (lines 430-475, 538-578).

[References]_________________________

Etienne RS, Cabral JS, Hagen O, Hartig F, Hurlbert AH, Pellissier L, et al. A minimal model for the latitudinal diversity gradient suggests a dominant role for ecological limits. The American Naturalist. 2019;194(5):E122-E33. Epub 2019/10/16. doi: 10.1086/705243.

E.g., do flat LDGs result if climate does not change? If extinction or speciation is prevented, but dispersal allowed?

**Response:** Using a new set of simulations, we showed that the LDG can rarely emerge even with static landscapes. See new Figure S4. However, the agreement of simulation with a static landscape with empirical data was lower when considering the other metrics.

2. Results:

a. How did speciation, extinction, and dispersal contribute to LDG for M2? I found the better fit of M2 interesting, but I wanted to know more. This is not discussed by the authors and seems critical to the authors' conclusions.

**Response:** We now calculated the respective rates from emergent dynamics as explained above. We also dedicate an entire new paragraph at the main text (lines 558-571).

b. The figures are very nice. What was interesting, however, was that the simulated LDG did not fit the empirical LDGs any better (or so it seemed based on Fig S6 and Fig. 4c) than the LDGs found by Rangel and colleagues and Saupe and colleagues. The perhaps poorer fit of model M2 to empirical data compared to previous models needs to be addressed, especially if the authors wish to highlight their conclusions throughout the manuscript (rather than use the 'case study' as a toy example).

**Response:** Indeed Rangel et al. found higher statistical fit between simulated and empirical patterns of species richness, but their domain of analysis is limited to South America, within a much narrower time scale. Conversely, here we aim at a much larger spatial (global) and temporal (65 millions of years) scales, which create serious difficulties to compare directly the R2 values between studies. Regarding Saupe et al. (2019), while the simulations did reproduce the LDG, the fit to the data was poorer (the strongest correlations had a R2=0.16 compared with a R2 of 0.36 (pearson cor=0.6) for Terrestrial Mammals and R2 of 0.32 (pearson cor=0.57) for Birds and Amphibians in our study), with poor reproduction of spatial patterns. In our case study, while we wanted to understand the emergence of the LDG, we made sure that our simulations had a good fit to the data. We now discuss this in the revised manuscript (lines 539-553, 631-634).

c. I was particularly intrigued that all models (M0, M1 and M2) resulted in LDGs (noted by the authors on line 473). This seems an important and interesting finding, and I would have liked to see more information on how close simulated patterns from M0 and M1 are to empirical gradients. What are the correlation coefficients between simulated and empirical curves, and what did the LDG curves look like in these cases?

**Response:** This is a great suggestion. We added this metric to the manuscript (Table 3) and calculated it as the standardized mean species numbers per latitude correlation between simulated and empirical maximal Pearson correlation. We now provide those numbers and figures also in supplementary materials. Using this metric showed an even clearer pattern to the data. For curves as requested, see new figure (Figure S5). For statistics involving the new methods see (Figure S4, S6, S13, S14).

3. Discussion:

a. The authors discuss the congruence of their patterns with Saupe et al, but they do not compare their results to Rangel et al's seminal work focused on South America, which analysed the effect of various parameters, including niche evolution (absent from Saupe et al's model) on LDGs.

**Response:** We have now included a new paragraph providing a brief comparison between our results and those found by Rangel et al. (2018) (line 580-601). We discuss the comparison of these models, current limitations of these models and of gen3sis as well as perspectives in the field, namely in the section Case Study: Synthesis (lines 579).

b. I assume the authors could not assess the effect of precipitation on speciation (found by Saupe and colleagues to be important), as the authors use only temperature as a variable to constrain niches?

**Response:** We used aridity since precipitation patterns are not available so far back in time. The explanation for aridity is explained in the lines (418-422) and (Note S1). We would like to further point out that our simulations have a much better fit to the data than the fit of the model of Saupe et al. (2019) where the support for the precipitation hypothesis is limited.

Minor:

Line 439 and 456: remove apostrophe and replace with comma

**Response:** We changed as suggested.

Line 101: perhaps not interoperability, but rather implementation of models by others

**Response:** We added this suggestion by framing implementation of models by others as generality and accessibility. We replaced ‘interoperability’ by ‘compatibility’ to convey the challenge of having multiple systems (lines 105-106).

Line 498: move the reference to the end of the clause, after 'of the LDG using computer models"

**Response:** We changed as suggested.

The references at the end of this sentence also do not examine LDGs sensu stricto, but do represent mechanistic computer models.

**Response:** We modified the sentence so that the wording referent to LDG stricto is correct. We now say that these are mechanistic computer models studies that are useful to inform on the formation of the LDG.

Lines 585-608: I think it would be useful for readers (enhance clarity) if these lines were moved to the introductory paragraph of the case study.

**Response:** We changed as suggested. In order to work the flow of the text, we moved discussion parts to a case study dedicated synthesis session. We appreciate that by doing this, we open the discussion and debate on current limitations in the field, and some big next steps in the area.

**Reviewer #2:**

I think the authors have gone to a lot of effort to address the comments from the last review and the paper is improved. I do believe that the R package that the authors have developed will be a useful tool. However, I'm afraid that I still feel that the empirical application of the model presented here is far from convincing. Given the authors claims that these kinds of complex models can provide new insights into large scale biodiversity patterns, providing a powerful example to demonstrate this is important. The authors now focus on using their model to make inferences about the causes of the latitudinal diversity gradient. They explore three different scenarios: a time for speciation model, speciation rate and carrying capacity model. The authors conclude that the carrying capacity model best fits empirical patterns in terms of matching the slope of the LDG, the slope of the range size frequency distribution and the shape of the phylogenetic tree (phylogenetic imbalance). That a model with a latitudinal gradient in carrying capacity best explain the observed LDG is a strong conclusion to make but I really don't think the application and fitting of the model is robust enough to say this.

**Response:** We realize that there was an issue with the way we present our case study. Our case study is not an explicit attempt to definitely answer the long standing questions associated with the LDG. The purpose of the case study is rather an attempt to showcase the flexibility and utility of our model and R-package and highlight challenges in the field, which we stress in the revised manuscript. The reviewers also seem to recognize the utility of such a general approach but we likely failed to communicate this scope, which is different from a full research study. Indeed, the mechanistic underpinning of the LDG is an outstanding question in biology, but the manuscript is rather aimed at presenting the gen3sis modelling engine. Because much of the space is used to present the simulation, it would be beyond the goal of the study to explore more hypotheses and more as expected from a full research paper. With this being said, we see the utility in the reviewer’s suggestion and wish to accommodate the reviewer as much as possible. We thus integrated all the propositions of improvement from the reviewer. In particular, we were now more careful with the conclusion, removing case-study statements from the abstract and modifying our conclusion to recognize that further hypotheses might be tested, while we further complemented our explorations (lines 595-599, 683-688).

One concern is the parameter values used. There seemed to be little to no justification for the choice of parameter values used and as far as I could tell the authors do not provide evidence showing that their conclusions are robust to a comprehensive exploration of parameter space. For the parameters they examine, only a handful of different values are used. I understand that with such a complex model it is very hard to comprehensively explore parameter space, but I feel this is an important weakness of the current approach being advocated here. Using slightly lower or higher values, or indeed some value intermediate to the ones you have chosen, could change the conclusion of which scenario fits best. Of course, this possibility can never be ruled out entirely, but I think just trying 3 or 4 different values for a parameter is just nowhere near sufficient. To me the current implementation of the simulation remains an illustrative, that can be used to make statements likes 'here are the kinds of patterns that can arise under different scenarios under these assumptions' but it cannot be used to robustly test hypotheses.

**Response:** We fully agree and we now do a much better exploration of parameters and show that our results are conserved. We explored a larger parameter space with 1,500 new simulations (as much as we could given the available revision time) within a continuous parameter space. We used sobol sequences, a quasi-random number generator that samples parameters evenly across the parameter space. We now provide more supplementary figures showing the resulting properties of simulations across the explored parameter space. We also acknowledge and discuss the limitations of parameter space exploration (line 593-599)

The authors only keep simulations that fit certain criteria e.g. fewer than 50,000 species. But it seems like for the scenarios they explored most simulations are retained. This suggests that a wider range of parameter values needed to be explored - ideally you want to explore the full breadth of parameter space, so that in one scenario all species go extinct before the end of the simulation while in another they all exceed some maximum imposed value. This would tell you that you are reaching the bounds of realistic parameter values and that there is no need to explore more extreme values.

**Response:** We agree and we explored broader parameter range in the revised manuscript. The parameters space used here derives from multiple sensitivity analyses. By having a broad abortion criteria, i.e. fewer than 50’000, we make sure that we consider as many simulations as possible, with almost no simulations reaching that threshold. Gen3sis flags simulations that were aborted and register which criteria was met. During the interactive modelling cycle, we explored the lowest speciation and dispersal parameters that generate a reasonable number of species and biodiversity patterns and that lay within the range of plausible parameter values (e.g. no dispersal below 1 site or no dispersal beyond biologically plausible values). Although we made the explored parameter space broader and continuous, we were limited by time and computing power allowed for the revision. We added a new paragraph discussing the need of further parameters and processes exploration and we expect this to be done in future studies with gen3sis (lines 683-688). We also reminded that the objective of this study is to illustrate the method rather than provide a full exploration of all mechanisms and associated parameter ranges.

The authors comparison of scenarios also seem incomplete. First, as far as I could tell from the notes the authors compare a model where there is a latitudinal gradient in carrying capacity (M2) to models (m0 and m1) where there is no carrying capacity. But what about the scenario where there is a carrying capacity but it doesn't vary across latitude? This scenario is needed in order to demonstrate whether it is the presence of a carrying capacity or the gradient in carrying capacity which drives differences in model fit.

**Response:** This is a great suggestion. We agree and modified the manuscript accordingly. This comment was also related to a remark from the first reviewer, who also suggested the use of null models. We now added several null models to also show the conditions necessary for the LDG to emerge. The null models, including a carrying capacity that does not vary with temperature (new M4) also produce a reasonable fit to the data compared to the model with carrying capacity varying with latitude such as temperature and aridity (old M1, new M5). This difference increases when penalizing for number of parameters accounting within spatial diversity comparisons (Figure S3). Nevertheless, M5 was still the only model passing all conditions. We further insist that while we wanted to illustrate several possible hypotheses, our case study does not cover all possible mechanisms. Further studies investigating the LDG and other large scale biodiversity patterns are needed (lines 599-602). We suggest that ecological limits could be important, but also acknowledge that we did not explore all possible mechanisms. We hope that the gen3sis engine will stimulate further studies which will tackle specific processes and mechanisms or a combination in generating global biodiversity patterns including the LDG.

I had a query about m1 and the gradient in speciation rates. As far as I could tell the gradient in time to speciation is ~ 3 (i.e. 3 times faster at the hottest sites) regardless of the parameter values chosen (e.g. 4/1.3 = 3.1 and 20/6.7 = 3). Perhaps I'm missing something here, but in that case a clearer explanation is needed.

**Response:** Correct. We kept the difference constant. We have on-going research that will exclusively explore multiple implementations for testing a variety of metabolic theories. In the present manuscript, the goal was not to perform a full exploration, but illustrate that the engine can serve to contrast different hypotheses from multiple categories (as grouped in Pontarp et al. 2019). We highlight that we only tested the shape of the slope but not the gradient (Figure S1). We now also include in the manuscript, that our case study is only illustrative and that these processes should be further explored in future research studies (lines 41, 131, 137, 208, 247, 365, 376, 384, 425, 539, 554, 597, 655, 663).

[References]_________________________

Pontarp M, Bunnefeld L, Cabral JS, Etienne RS, Fritz SA, Gillespie R, et al. The latitudinal diversity gradient: Novel understanding through mechanistic eco-evolutionary models. Trends in Ecology and Evolution. 2019;34(3):211-23. doi: 10.1016/j.tree.2018.11.009.

When comparing scenarios, the authors identify which model best matches the empirical data even though the number of parameters varies between models. Some kind of penalisation for model complexity seems necessary here.

**Response:** We agree that a model complexity penalisation can be included to further compare models. We computed the number of free parameters of each simulation and evaluated a global match of simulations to the spatial richness patterns. This metric of match was then pondered by the number of parameters using a BIC criteria (lines 521-521, 548-550) as used in previous simulation studies (Leprieur et al. 2016).

[References]_________________________

Leprieur F, Descombes P, Gaboriau T, Cowman PF, Parravicini V, Kulbicki M, et al. Plate tectonics drive tropical reef biodiversity dynamics. Nature Communications. 2016;7:11461. doi: 10.1038/ncomms11461.

Also, the authors narrow down parameter space by simulating on a coarse grid of 4 degrees, before then applying those parameters at a finer resolution. To me this doesn't seem justifiable.

**Response:** Again, here, we wanted to demonstrate the feasibility of running simulation at very high resolution, but not to provide a systematic comparison of the two resolutions. We removed this aspect in the revised manuscript. Hence, this high resolution simulation should be seen as illustrative only and the main analyses are done at coarser resolution.

The best parameters for explaining the spatial and phylogenetic patterns will almost certainly vary with the number of grid cells in the simulation. This will apply to both the absolute parameter values (e.g. a coarser grid with fewer cells will lead to lower richness) and relative parameter values (e.g. a coarser grid with fewer cells will limit the extent if variation in range size among species and thus tree imbalance).

**Response:** We agree that simulations at different resolutions cannot be compared. At the coarser resolution, all simulations have the same number of cells. We removed the systematic comparisons of the simulations at two resolutions and we better explained it in the revised manuscript (lines 505-507).

Another issue is the choice of statistics used to compare empirical and simulated patterns. In table 2, the authors present a long list of spatial and phylogenetic metrics that their model can be used to predict. Why then only use three statistics and why these three?

**Response:** Our goal was to use the LDG as an illustration and use three “families” of evaluation metrics, while of course we are aware that many more metrics exist and could be used. First, we added three more metrics to compare the simulations to empirical to discriminate among the different models tested. Second, in the discussion, we now clearly point out that our exploration of metrics is only partial and that future broader studies of the LDG should consider more hypotheses and more metrics for discrimination. The study of the LDG is decades old and we are aware that even with a new methodology, it remains a complex problem to tackle and it is not the objective to solve this old problem in our illustrative case study. We also were more careful about our conclusion of carrying capacity and suggest that further explorations using Gen3sis are necessary. We discuss in the revised discussion that further metrics could be used to include more empirical dimension for the evaluation of simulations (lines 682-687). In addition, we added the nLTT, LDG curve and alpha diversity metric as explained below.

The choice seemed arbitrary and perhaps not obvious. If the model makes predictions of richness in each cell why not compare the spatial patterns rather than just the LDG slope which throws so much information away? Why look at phylogenetic imbalance? Given that one of the hypotheses the authors are testing is whether there is a carrying capacity to diversity, the temporal dynamics of species diversification would have seemed an obvious, indeed necessary, metric to include. Metrics have been developed for matching empirical and simulated LTT plots that I think would be more informative (e.g. Janzen et al 2015). Related to this, none of the metrics capture absolute species richness or range size. So, it could be that the parameter values that give the best match according to the 3 summary statistics chosen give implausible levels of richness. The metric of Janzen et al 2015 would help to address this. In short, I would be very sceptical that the metrics chosen here can robustly discriminate between the three different scenarios. I would also be concerned about the sensitivity of some of these statistics to other choices in the model and I don't think the current study presents a strong case for understanding how these statistics depend on the different assumptions of the model or the parameter values chosen. For instance, previous studies have shown that there are many factors that can cause differences in phylogenetic tree imbalance, from the geographic mode of speciation to assumptions about how species compete. Perhaps in the simulations the authors explored, observed tree imbalance is best matched by the model with a varying carrying capacity but change the way you model speciation or competition and this may no longer be the case.

Janzen, T., Höhna, S. and Etienne, R.S. (2015), Approximate Bayesian Computation of diversification rates from molecular phylogenies: introducing a new efficient summary statistic, the nLTT. Methods Ecol Evol, 6: 566-575. https://doi.org/10.1111/2041-210X.12350

**Response:** We agree. In the revised manuscript, we have applied both correlations of alpha-diversity (while also accounting for model complexity) as well as nLTT to compare the simulations from the different models (Table 3, lines 534-536, Figure S4). Adding these statistics did not change the ranking of the models. Alpha diversity correlation was superior for models M4 and M5 and clearly better compared with simulation using static landscapes without deep-time dynamics. On the other hand, the nLTT metric did not allow strong discrimination between models (Figure S4). We found that nLTT did not clearly allow to discriminate among models, as alpha-diversity and the LDG slopes or curve fit could (Figure S4, S13 and S14).

Furthermore, we did not use absolute values of range size and absolute richness because the absolute numbers are likely to be the result of partly stochastic processes and thus very difficult to reproduce in silico. In contrast, biodiversity gradients and other general statistics are more realistic targets for simulations studies, now discussed in the revised manuscript (lines 682-687) as follows: “Moreover, modelling eco-evolutionary processes in a flexible platform, enables the exploration of biodiversity statistics in dependence on a multitude of the different model assumptions and parameter values. This approximates how biodiversity patterns relate with eco-evolutionary processes. Further studies exploring dependencies of summary statistics with model assumptions or parameters are necessary and could be readily assisted by gen3sis”. Moreover, we added an analysis investigating how the simulated LDG curves compare with those of empirical data (Table 3).

We now mention in the discussion that although we are far from a full and detailed understanding of how biodiversity patterns emerge from eco-evolutionary processes in a dynamic environment, we show that some long-standing general patterns can be reproduced. By modelling eco-evolutionary processes in a flexible platform, we enable the exploration of how general biodiversity patterns can emerge from a variety of the different model assumptions and parameter values. We mention that future research can use Gen3sis to study more hypotheses, broader range of parameters and use a broader set of patterns for the comparison with empirical data (lines 682-687), but which goes beyond the illustrative goal of the present method manuscript.

L393 -What is the justification for assuming a globally distributed species? How does this initial condition influence the relative fit of the models?

**Response:** Paleontological evidence indicates that the LDG only appeared in the late Eocene (e.g. Marcot et al 2016) and by then the main clades investigated in that study (i.e. mammals) were widespread at that time. Hence assuming widely distributed lineages at the start of the simulations is justified. We added this citation to the revised manuscript (line 436).

[References]_________________________

Marcot, J. D., Fox, D. L., & Niebuhr, S. R. (2016). Late Cenozoic onset of the latitudinal diversity gradient of North American mammals. Proceedings of the National Academy of Sciences, 113(26), 7189-7194.

L405 - it is not immediately clear how this is representing the 'time for species accumulation' model as the simulation starts with a single globally distributed species rather than one restricted to the tropics. I assume this is because at the start of the simulation most environments on Earth were hot and thus this is the ancestral niche. if that is the rationale then I think this needs to be spelt out more clearly.

**Response:** Indeed, this is the reason, we explained this better in the revised manuscript (lines 435-437).

L407 - Only temperature considered in site suitability? What about precipitation?

**Response:** We used an aridity index obtained from paleo-indicators of dry climate. We modified the text to explain this index better (lines 136, 388, 442-443, 465-467, 470).

**Reviewer #3:**

I'm happy with the edits to the manuscript - this is a great paper and package. I'll add that I did not expect this when I started the review, but the author's comments and edits really convinced me of the usefulness of the engine presented here.

**Response:** Thank you for the positive feedback. We are glad that you found our revised manuscript to make these points clear and that the paper and package will be great contributions.
