# Supplementary material for: gen3sis: A general engine for eco-evolutionary simulations of the processes that shape Earth’s biodiversity
Source: PLoS Biol. 2021 Jul 12;19(7):e3001340. doi: 10.1371/journal.pbio.3001340 (PMC8384074; doi:10.1371/journal.pbio.3001340)
Supplement: Attachment — Submitted filename: Response_Reviewers_gen3sis_R3.docx [file pbio.3001340.s025.docx]

**Reviewer #1:**

The authors have done a nice job with their revisions, and I am now mostly satisfied with the manuscript. The model is well described, and its utility is apparent. I also appreciate how the authors have made their LDG example just that: an example. The authors have downplayed the results and discussion of the LDG analyses throughout, and I think this greatly helps with clarity and alleviates the concerns that I (and the other Reviewer) had. I still find issues with some of their LDG work. However, I think these issues become less important if the authors can provide even more explicit discussion that the analyses are simply illustrating the utility of their framework, and they are not meant to make broad claims about LDG generation.

Response: We thank the reviewer for their positive feedback on the communication of our findings. In the revised version, we make it even clearer that our analyses are illustrative and not a full empirical study of the LDG. Specifically, we made the following modifications:

Changed lines 380-382:

“From these hypotheses, we illustrate five derivative models in gen3sis; a null model without ecological filtering or trait evolution (M1), a model ...”

Changed lines 554-555:

“We implemented five illustrative gen3sis models derived from hypotheses on the emergence of the LDG.”

Changed lines 557-559:

“As a simplified approach for this illustration, all simulations were initiated with one single ancestor species spread over the entire terrestrial surface of the Earth at 65 Ma [134] ...”

Changed lines 498-501:

“After parameter range exploration, we identified realistic parameters and ran a subset at 1° for high-resolution outputs for illustration (Figure 4). Parameter exploration is illustrative and could be expanded in future research applications.”

Changed lines 587-589:

“Although our case study was illustrative and we implemented only a small representative fraction of the candidate processes and parameters expected to shape biodiversity patterns, it illustrates how gen3sis can handle multiple interacting eco-evolutionary processes proposed in the literature.”

1)	I am not convinced by the author's justification for assuming a globally distributed species and how this affects the initial condition for inference of the models.

Response: In this section we explore general simulations as an illustration of the methodology, but we agree that we make some simplifications in the exploration of the parameter. In particular, assuming a globally distributed ancestor leads to a more parsimonious exploration of the parameters. Moreover, this makes the example more general and less specific to a particular group or organisms. In future case studies, the model could be used to match very specific taxonomic groups.

Added lines 429-432:

“As a simplified approach for this illustration, all simulations were initiated with one single ancestor species spread over the entire terrestrial surface of the Earth at 65 Ma [134], but initial conditions could also match the ancestral range informed by fossil records [47].”

2)	What does 'modelled process of environmental filtering' mean? (Abstract)

Response: We agree that this was unclear and broad. Thank you for spotting this. We reformulated the sentence to specify filtering abiotic constraints and biotic interactions, thereby improving clarity.

Changed lines 38-39;

“Modelled processes can include species’ abiotic tolerances, biotic interactions, dispersal, speciation and evolution of ecological traits.”

3)	The authors partition existing hypotheses for the LDG into three broad categories: (i) time for species accumulation and niche conservatism, (ii) variation in diversification rates, and (iii) variation in ecological limit. Although useful to categorize the many existing hypotheses for the LDG, the categories devised by the authors seem inaccurate. For example, time to species accumulation does not need to invoke niche conservatism. Indeed, the 'tropics as older' hypothesis is primarily about how climate in the tropics has been stable for longer, which allows more species to accumulate because of reduced extinction. Niche conservatism can play a role in this hypothesis, but it does not need to do so. The second hypothesis (variation in diversification rates) should also include discussion of the tropics as a cradle vs museum. Rates of speciation and extinction can vary across latitudes for more reasons than increased metabolic and mutation rates. Finally, the third category invoked by the authors (variation in ecological limit) is also, ultimately, about reduced extinction due to differing ecological limits. This section needs to be made clearer, or - alternatively - the authors should focus solely on their subset of hypotheses, without making claims to the broader set of existing LDG hypotheses.

Response: Thank you for this helpful comment. We realize that this comment stems from the difficulty in teasing apart processes within general claims. For example, in the case of the “tropics as older” hypothesis, the confounding effects of time might be linked with the environmental stability regime; requiring further experiments targeted at climate stability and time to accumulate species. We followed your second suggestion and modified the section to focus on the subset of hypotheses that we implemented. (Case study: context).

Moreover, we added lines 373-375:

“Among the proposed hypotheses, it has been postulated that older and more stable tropical environments have more time for cumulating species and have reduced extinctions, while niche conservatism limits the spread of lineages to more recent colder environments [59-62].”

4)	I am still concerned about the authors' protocol of removing simulations if they do not meet certain criteria. It is easy to force simulations to match empirical data if you only require the simulations to do so some of the time. This, however, does not necessarily reveal anything deep about underlying mechanisms. For example, the authors throw out any simulations that do not match the following acceptance criteria: (i) LDG between 5.4% and 1.1%, (ii) tree shape statistic, β between -1.4 and -0.3, and (iii) range size frequencies with a decrease in the number of large-range species with a tolerance of 5% (Line 533).

Response: We reformulated this section (lines 516-532). The “pattern oriented modelling” approach (Grimm & Railsback, 2012) does not involve removing simulations, but rather investigating how many simulations match the empirical data considering a confidence interval. This approach has been used in the context of many process-based modelling applications in previous studies (Gallagher et al. 2021).

[References]_________________________

Grimm, V., & Railsback, S. F. (2012). Pattern-oriented modelling: a ‘multi-scope’for predictive systems ecology. Philosophical Transactions of the Royal Society B: Biological Sciences, 367(1586), 298-310.

Gallagher, C. A., Chudzinska, M., Larsen‐Gray, A., Pollock, C. J., Sells, S. N., White, P. J., & Berger, U. (2021). From theory to practice in pattern‐oriented modelling: identifying and using empirical patterns in predictive models. Biological Reviews.

5)	One of the best models found was M4, which imposed a carrying capacity. However, this carrying capacity did not vary by latitude. I was curious, then, what caused the LDG to emerge in M4? Do the authors have insight into this?

Response: The LDG emerged because of niche conservatism, which is also included within M4. Since the tropics are older they had more time to accumulate species. However, considering an overall carrying capacity for a cell as a simple biotic interaction in M4 led to simulations that better matched the empirical data. Our results suggest that some level of saturation in species accumulation better corresponds with empirical data, as suggested by empirical analyses of phylogenies in earlier studies (Lewitus & Morlon 2016).

[References]_________________________

Lewitus, E., & Morlon, H. (2016). Natural constraints to species diversification. PLoS biology, 14(8), e1002532.

6)	The authors indicate that their results "corroborates with a contribution of both speciation [22], extinction [6] and dispersal [149] in shaping the LDG" (Line 567). However, the authors found that dispersal occurs out of the tropics, not into the tropics (Line 564). Thus, I wondered how dispersal could contribute to the LDG in this case? Dispersal in this instance seems to be occurring in the reverse direction to elevate diversity in the tropics.

Response: We found that dispersal was stronger from the tropics towards the temperate zone than vice versa. Thus, dispersal indeed was shown to be directional from low to high latitude, leading to increased diversity at higher latitudes. As a result, this process rather decreased the slope of the LDG, as found in empirical studies (Jansson et al. 2013). Because diversity was higher in the tropics, species were more likely to move from low to high latitude. This point is clearer in the revised manuscript (L. 586).

Added lines 561-562:

“Because diversity was higher in the tropics, species were more likely to move from low to high latitude, corroborating empirical observations [149].”

[References]_________________________

Jansson R, Rodriguez-Castaneda G, Harding LE. What can multiple phylogenies say about the latitudinal diversity gradient? A new look at the tropical conservatism, out of the tropics, and diversification rate hypotheses. Evolution. 2013;67(6):1741-55. Epub 2013/06/05. doi: 10.1111/evo.12089.

Line 252: population instead of populations?

Response: We modified as suggested. In addition, the final version of the manuscript was fully checked by a professional English editor.

Line 485: distribution, not distributions?

Response: We modified as suggested.

Line 580: South America, not South American

Response: We modified as suggested.

Line 596: Consider rewording to "we made sure to select parameters based on a range supported by the literature"

Response: We modified to a similar statement.

Changed lines 473-475;

“We explored parameter ranges by basing upper and lower parameter boundaries on the literature and interactive modelling explorations.”

Line 648-651: consider rewording, as this sentence is a bit confusing.

Response: The sentence was modified as follows:

Changed lines 640-643:

“The combination of exploring patterns emerging from models and qualitatively and quantitatively matching their outputs to empirical data should increase our understanding of the processes underlying global biodiversity patterns.”

**Reviewer #2:**

While I remain skeptical about the utility of these very complex models and would have expected to see a really impressive empirical demonstration first (the method would then sell itself!), I think that the computer model that the authors present will be a useful tool for others and will hopefully encourage further advances.

Response: As seen in other fields, we think that a combination of correlative approaches and more complex process-based models will drive advances. We thank the reviewer for the critical and fair feedback, which helped us to significantly improve the manuscript presenting this method.

The authors have undoubtedly improved the implementation and analysis of their simulations examining different explanations for the LDG. They have also toned down their interpretation of these results, admitting that these should be viewed as a preliminary exploration. So, I think this is now much more balanced and robust. In short, the authors have addressed my comments.

Response: We are glad that the modification of the presentation of the case study, as an illustration rather than a full empirical demonstration, made the manuscript more balanced.
